# A Multi-Strategy Crested Porcupine Optimizer for Work–Family Resource Allocation: Childcare Facility Siting and Family-Friendly Shift Scheduling

**DOI:** 10.3390/biomimetics11100720

**Published:** 2026-10-09

**Authors:** Yang Shi, Xinyu Cai

**Affiliations:** 1 School of Labor Economics, Capital University of Economics and Business, Beijing 100070, China; 2 School of Accounting, Shandong Youth University of Political Science, Jinan 250103, China; 3 College of Business, Jiaxing University, Jiaxing 314001, China

**Keywords:** crested porcupine optimizer, swarm intelligence, quasi-opposition-based learning, elite-guided search, facility-location-allocation

## Abstract

The time cost of care constrains female labour supply; two policy levers act on it: where childcare capacity is sited and how shifts are scheduled, both large combinatorial problems. The crested porcupine optimizer (CPO) initialises at random, guides exploitation by a single random or best individual and resizes its population on a fixed cycle. The proposed multi-strategy CPO (MSCPO) keeps CPO’s four defence operators and replaces those original components with Sobol quasi-opposition initialisation, elite-guided exploitation and monotone fitness-based population reduction. On the 29 CEC2017 functions at 10, 30, 50 and 100 dimensions (30 runs of 30,000 evaluations), MSCPO had the best Friedman mean rank among ten algorithms at every dimension (1.29 against 2.53 for CPO; χ^2^(9) = 878.5, *p* < 0.001), and pooled Holm tests separated it from all nine competitors (*p* ≤ 0.002). A factorial ablation attributed about 70% of the gain to elite-guided exploitation. The advantage over CPO vanished at the full budget of 10^4^*D* evaluations. In childcare siting, MSCPO ranked first, leaving the dispersion of access 28–35% below the most equal siting rule with at least 99% of the best rule’s employment; in shift scheduling, it ranked fifth, with instrumentation suggesting a limitation of large steps under a rank-key decoder.

## 1. Introduction

The comparison algorithms are selected to test whether the proposed variant improves on its parent algorithm and remains competitive with other population-based search designs. Particle swarm optimisation (PSO) [1] and the grey wolf optimizer (GWO) [2] provide established guidance rules; the whale optimisation algorithm (WOA) [3] and Harris hawks optimisation (HHO) [4] provide established exploration–exploitation designs. The dung beetle optimizer (DBO) [5], secretary bird optimisation algorithm (SBOA) [6], black-winged kite algorithm (BKA) [7] and red-billed blue magpie optimizer (RBMO) [8] extend the comparison to algorithms published in 2023–2024. These eight methods are empirical baselines. Differential evolution (DE) [9] supplies the closest precedents for the proposed component changes: success-history based parameter adaptation (SHADE) [10] adapts mutation parameters, whereas the proposed variant keeps the defence constants fixed; linear population size reduction (LPSR) [11] progressively reduces the population, the principle adopted by the proposed reduction schedule; and multi-stage parameter adaptation with diversity enhancement [12] illustrates search-state control that the proposed variant does not implement. The no-free-lunch theorem [13] motivates testing transfer to two application classes instead of inferring general superiority from benchmark ranks.

The crested porcupine optimizer (CPO) [14] supplies the four defence operators retained by the proposed variant. Sight and sound drive exploration, while odour and physical attack drive exploitation; the working population contracts and is restored on a fixed cyclic schedule. Published applications establish different uses of this search engine. CPO has been used to tune photovoltaic forecasting models [15] and wind-power forecasting models [16], optimise thermoelectric-generator design variables [17], and tune battery state-of-health prediction models [18]. These studies motivate using CPO for costly objective evaluations, but do not establish its suitability for the spatial equity and decoded-roster objectives studied here. Unmanned aerial vehicle (UAV) delivery path planning [19] provides a constrained application precedent; its different representation likewise makes transfer an empirical question.

A systematic review conducted under the PRISMA-2020 (Preferred Reporting Items for Systematic Reviews and Meta-Analyses) guideline extracts 117 improvement strategies from 43 CPO variants, with perturbation, initialisation and adaptive parameter control accounting for 38%, 27% and 23%, respectively [20]. This taxonomy motivates separating the three modifications in the proposed variant rather than treating a combined variant as a single intervention. The primary variants identify premature convergence or insufficient diversity [21,22], local-optimum trapping [23], slow convergence [24] and static parameters [25]; their specific remedies are compared with the proposed variant in Section 3.6 and Table 1. An independent photovoltaic-parameter comparison places the Growth Optimizer ahead of CPO under the Friedman ranking [26]. This result illustrates the problem dependence of CPO’s performance; it does not identify which modification will improve the present applications.

These reports agree on the symptom but say little about which operator produces it, and the remedies are correspondingly generic. Reading CPO’s update rules directly isolates three separable, independently repairable design choices. First, the initial population is drawn independently and identically distributed (i.i.d.) from a uniform distribution, leaving coverage of the feasible box to chance, although the choice of initialisation can affect final quality, to a degree that depends on the algorithm and the problem [28]. Second, the two exploitation operators make poor use of the good individuals: the third defence draws its base vector from one uniformly random individual, ignoring fitness, and the fourth pulls every individual it updates towards the single incumbent best (Equations (10) and (12) in Section 2.2, as specified in [14]). The third operator has no fitness preference when selecting its guide, whereas the fourth uses the incumbent best as a shared guide. This concerns guide selection; fitness-dependent terms and greedy acceptance still operate in CPO. Two methodological precedents motivate testing multiple guides. Comprehensive-learning PSO uses other particles’ personal-best exemplars [29], while JADE, an adaptive DE algorithm, samples a guide from a top-ranked fraction in current-to-pbest mutation [30]. The proposed elite-guidance strategy adopts the latter sampling principle inside CPO’s exploitation equations, without adopting either algorithm’s update rule. These precedents motivate the modification; its effect in CPO is tested in Section 4.3 and Section 4.5. Third, the population schedule of Equation (2) is periodic: it contracts the working population and then restores it on a fixed cycle, handing evaluations back to breadth late in the run, whatever the function, the dimension or the state of the search, and the individuals that are left out are chosen by their position in the array, not by fitness. This reading is a conjecture, not a result: premature convergence is a diversity-loss phenomenon that must be measured, not assumed [31], so it is instrumented with dimension-wise diversity decompositions [32,33] (Section 4.5) and tested by a full factorial ablation (Section 4.3). The ablation supports the second point strongly and the first only weakly; for the third, an additional variant that keeps CPO’s floor of 0.8N shows that the gain comes from the smaller population reached late in the run rather than from removing the periodicity.

We therefore propose the multi-strategy CPO (MSCPO), which retains the structure of the four defence operators and their constants while changing three design choices. Sobol quasi-opposition initialisation (SQI) combines low-discrepancy sampling, used in a recent swarm optimiser [34], with quasi-opposition-based learning (QOBL). Opposition-based learning (OBL) supplies exact opposite candidates [35,36], whereas QOBL samples between the midpoint and the opposite point [37]; its use in a recent marine-predator variant [38] supports the relevance of complementary initial candidates, without transferring that variant’s Q-learning mechanism. SQI evaluates the pooled 2N candidates and retains the N fittest. Elite-guided exploitation (EGE) samples from the fittest fraction, following the guide-selection principle of JADE [30], and applies it to both CPO exploitation operators. Adaptive population reduction (APR) follows the monotone reduction principle of LPSR [11], with fitness-based truncation and a larger terminal size. The factorial ablation distinguishes the contribution of each change.

A second weakness of this literature is its validation portfolio. New algorithms and reworked update strategies are certified almost without exception on the IEEE Congress on Evolutionary Computation (CEC) suites and the same small set of constrained engineering design problems, and the applications that follow cluster in the same engineering and energy domains, as CPO’s record illustrates. Benchmark suites also carry known biases, such as optima clustered near the centre of the search space [39], so structurally similar problems cannot discriminate between algorithms that will and will not transfer.

Work–family resource allocation provides a test of transfer to spatial planning and decoded scheduling objectives. The binding constraint on female labour supply is time: care must be supplied by someone, at particular hours and in a particular place, and two policy levers act on it—where care capacity is sited, and when employees are required to work. Child penalties in employment are modest at low development levels but become the dominant source of gender inequality as economies industrialise [40], the long-run earnings penalty reaching 52 log points in Italy over 1985–2018, mainly through reduced weeks worked [41]. In China, these objectives are coupled: the motherhood penalty is implicated in continued low fertility [42] and in the urban gender wage gap [43], and it is cushioned by grandparental care, grandmothers reducing their own labour supply to provide it [44], and liberalising the birth quota without relieving the care burden produced the outcome a planner would most fear—female labour force participation fell by 4.7 percentage points after the universal two-child policy, the slack absorbed by grandparents, not public provision [45]. Provision for the under-threes is itself decentralised and uneven across provinces [46].

Both levers have been measured precisely, but only ex post. The maternal participation response is 0.2 percentage points per point of coverage in Germany [47] and 0.27 in Japan [48], reaches 12 points in a randomised expansion in Nicaragua, where it measures the effect of attending a centre rather than of coverage [49], is positive in four of seven harmonised European settings and null in the rest [50], and is sharply non-linear in hours, part-time entitlement being nearly inert where full-day entitlement binds [51]. These estimates come from different countries, periods and designs, and they agree in sign. A 2023 review of child penalties discusses childcare access as a policy channel, with effects depending on existing provision and mothers’ labour-market attachment [52], although harmonised European estimates find no effect where even mothers of older children seldom work [50]. A 2023 review of lower- and middle-income countries finds positive effects in almost every causal study, while stressing variation in programme design and affected subpopulations [53] (Section 5.1.1). Schedule instability damages well-being largely through work–family conflict [54], predicts turnover, partly through the same channel [55], and workers would give up about 20% of wages to avoid employer-set, short-notice schedules [56]; meta-analytic evidence shows that longer working hours are associated with more work-to-family conflict across countries [57], while a 2024 meta-analysis supports distinguishing time-, strain- and behaviour-based conflict when designing its burden measure [58]. Yet schedule instability can be reduced without operational loss: more predictable schedules raised productivity by 5.1% while cutting hours [59]. Such designs are ex post by construction: they identify the average effect of a reform that was enacted, not the best configuration affordable under a budget. The cost is measurable: siting rules targeting neighbourhood income or excess demand achieve roughly 60% of the maternal-employment gains of optimal placement [60]—two fifths of the return lost to placement, not to budget size. The figures for the long-run earnings penalty [41], for the value workers place on predictable schedules and the productivity gain from them [56,59], for the return to optimal placement [60] and for the fall in participation after the universal two-child policy [45] each come from a single study in one country and period. They are quoted as orders of magnitude that motivate the models and do not enter either objective function: the only behavioural parameter of the two models is the response slope of the childcare model, which is calibrated in Section 5.1.2 and varied in Section 5.1.4, and the weights of the scheduling model are set by the planner (Section 5.2.2). Elasticities and valuations of this kind are the coefficients an ex ante design model needs inside its objective function. What is missing is the model.

The two applications require distinct operational-research formulations. Accessibility-based covering models [61,62] motivate graded spatial coverage in the childcare model; joint location-and-sizing formulations [63,64] motivate choosing both sites and capacities; and fairness-oriented covering models [65,66] motivate measuring the distribution of access across zones. Related facility-location problems admit both exact and heuristic approaches. Exact formulations, branch-and-cut, branch-and-price and variable neighbourhood search have been developed for a robust capacitated p-median problem [67], while binary metaheuristics have been compared for uncapacitated facility location [68]. These are computational precedents, not scalability results for the childcare model. For the scheduling model, hidden-Markov hyper-heuristics [69] motivate selection among rostering moves, and co-evolutionary non-dominated sorting genetic algorithm II (NSGA-II) [70] provides a multi-objective workforce-scheduling precedent. Fairness-oriented crew rostering [71] motivates equity within a roster, and fairness over time [72] identifies an extension beyond the single horizon used here. Fatigue-aware scheduling [73] motivates including rest-related penalties; personalised and evolutionary work–life-balance formulations [74,75] motivate employee-specific burden terms. The decoded objectives contain discontinuities and rank-dependent terms, making direct gradient-based search unsuitable without reformulation. The comparison tests continuous population methods on these objectives; no exact solver or problem-specific rostering method is benchmarked here.

The contributions of the paper are as follows.

(1) A structural diagnosis of CPO and a matched remedy: We conjecture that CPO’s documented premature convergence and stagnation problems originate in three design choices—uniform initialisation, single-source exploitative draws and a periodic population schedule that drops individuals by array position—and propose MSCPO, whose SQI, EGE and APR strategies address them one for one, leaving the defence mechanisms and constants untouched.

(2) A benchmark evaluation designed to be falsifiable: MSCPO is assessed on the full CEC2017 suite at 10, 30, 50 and 100 dimensions over 30 independent runs against CPO and eight comparators. The evaluation includes component ablation and fair FE accounting, following methodological guidance for bio-inspired comparisons [76], and applies non-parametric Friedman, post hoc and Wilcoxon procedures [77]. Performance is reported at both 30,000 FEs and the full CEC2017 budget in Section 4.7, following recommendations to compare algorithms across FE budgets [78]. The full-budget experiment bounds the claims drawn from the main comparison.

(3) Two new work–family optimisation benchmarks: We formalise two problems, childcare facility-location-capacity (CFLC) planning and family-friendly shift scheduling (FFSS), the former with a Gaussian two-step floating-catchment area (G2SFCA) accessibility statistic and a calibrated maternal labour-supply response, giving eight instances spanning 20 to 630 decision variables; Section 5 links the two.

(4) A test of application transfer: The two models link spatial childcare capacity and employee scheduling to explicit work–family objectives. MSCPO leads the CFLC comparison but ranks fifth on FFSS, so the application study identifies both a useful setting and a representation-dependent limitation of the proposed search.

The remainder of the paper is organised as follows. Section 2 reviews CPO and its improvements. Section 3 develops MSCPO. Section 4 reports the CEC2017 study. Section 5 formulates and solves the two allocation problems. Section 6 compares the findings with related work and sets out directions for further research, and Section 7 concludes.

## 2. The Crested Porcupine Optimizer

CPO [14] is the reference search engine for this study. Its four defence behaviours define the operators modified in Section 3, and its cyclic population schedule defines the alternative to APR. Unlike LPSR [11], which reduces population size monotonically and removes the worst individuals, CPO contracts and restores its working population on a fixed cycle. Equations (1)–(14) specify the baseline used here; the population-floor convention of the present implementation is stated in Section 2.2.

The relevant CPO variants are those that change the same components as MSCPO. SDHCPO [21] combines Sobol-opposition initialisation with parameter control and crossover, while HCPO [22] combines Sobol-Logistic initialisation with cellular neighbourhoods. They are the nearest initialisation and information-sharing precedents, respectively. CAPCPO [23], ECPO [24] and the targeted-defence variant [25] modify or augment defence updates, providing a contrast to changing only the guides while retaining the update structure. BTCPO [27] adds butterfly-search and triangular-walk steps, another operator-augmentation design. ICPO [19] removes cyclic reduction, providing a population-management contrast rather than the monotone fitness-truncation rule used by APR. Multi-objective CPO formulations use non-dominated selection [79] or reference-point guidance [80]; they are relevant to the future Pareto extension in Section 6.4, while MSCPO here remains single-objective. Table 1 compares these component choices, and Section 6.1 relates them to the present ablation and its limits.

The volume of this repair work reflects limitations that the primary sources state explicitly. Premature convergence is reported independently by three variant papers: SDHCPO is motivated by the observation that the original CPO is prone to premature convergence and insufficient population diversity [21], HCPO adds limited solution accuracy to the same two defects [22], and the targeted-defence variant [25] lists premature convergence alongside poor initialisation and static parameters that cannot adapt to the phases of the search; the earliest multi-strategy variant records that CPO falls into local optima [23]; slow convergence, limited accuracy and susceptibility to local optima are reported again in motivating ECPO [24]; and the systematic review names persisting stagnation in high-dimensional problems, together with the absence of theoretical convergence analysis, as defects that the field has not resolved [20]. In the multi-objective setting, the same difficulty reappears as an unresolved tension between convergence and diversity [80]. Independent evidence points the same way: in a third-party comparison of ten recent metaheuristics on photovoltaic-parameter estimation, CPO did not rank first under the Friedman test [26], suggesting that its advantage is problem-dependent rather than universal. The formal description of CPO on which the proposed modifications build is given next.

### 2.1. Biological Inspiration

The crested porcupine (Hystrix cristata) does not meet a threat with a single response. It escalates. A distant or hesitant predator is met with a visual display: the animal erects and fans its quills so that its apparent size doubles. If the predator persists, sound is added; the hollow rattle quills at the tail are shaken to produce a warning rattle. If that fails, a pungent secretion is released from a gland beneath the tail. Only when the first three signals have all been ignored does the porcupine turn and charge backwards, driving its quills into the attacker. The sequence is economical: cheap, wide-reaching signals are tried first, and the costly, close-range response is kept for last.

**Figure 1:**
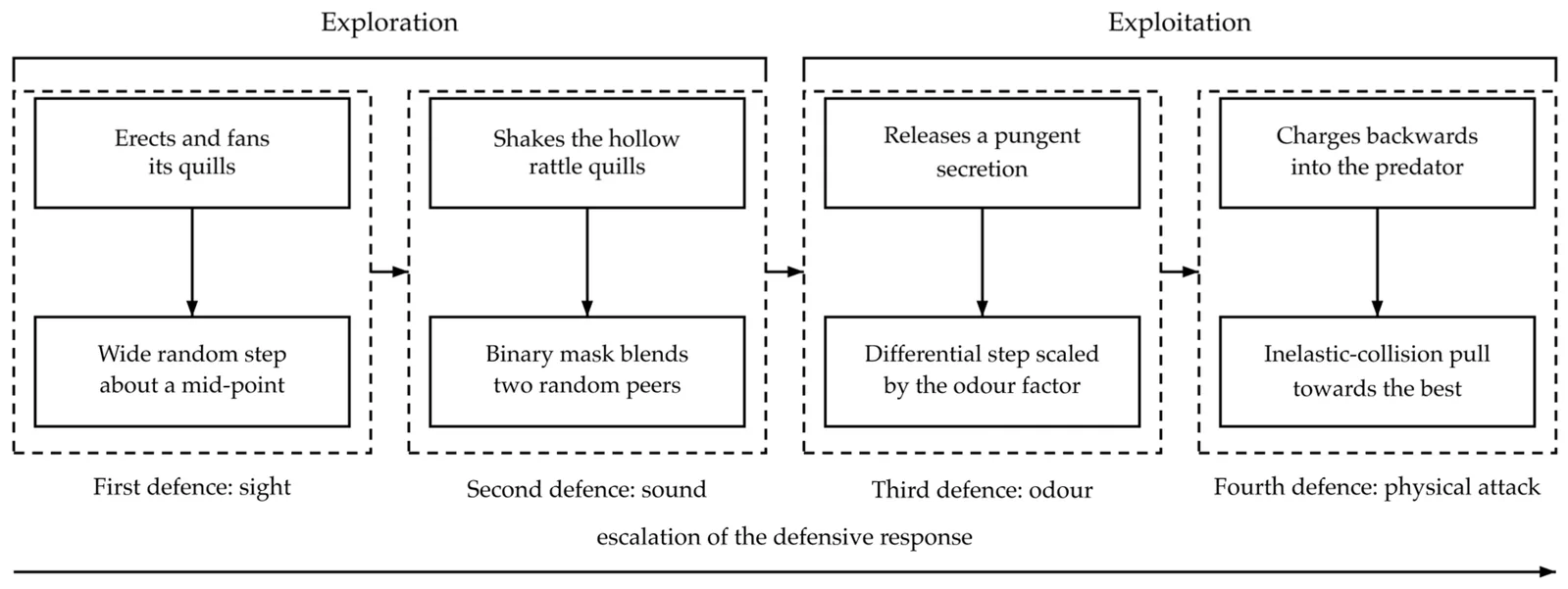
Mapping of the four escalating defence behaviours of the crested porcupine onto the search operators of CPO.

CPO turns that escalation into a search schedule [14]. The first two defences are modelled as exploration operators, because a visual or acoustic signal acts at a distance and therefore corresponds to a large, weakly directed step in the search space. The last two are modelled as exploitation operators, because an odour gradient and a physical charge act locally and are strongly directed towards the source of the threat. Figure 1 summarises the correspondence. A second element of the metaphor, the fact that only a fraction of the colony is under threat at any moment, motivates a cyclic population reduction rule that lets the number of individuals actually updated in an iteration rise and fall during the run.

### 2.2. Mathematical Model

Consider the minimisation of a D-dimensional objective f(x) over a box [lb,ub]. CPO maintains a population of N candidate solutions that is initialised uniformly at random, as given by Equation (1).

(1) $$x_{i}^{0}=lb+r⊙(ub-lb),\;i=1,2,\ldots ,N$$

where r is a vector of independent uniform variates on [0,1] and ⊙ denotes the elementwise product. Throughout, t counts the consumed function evaluations (FEs), T is the evaluation budget, $x_{CP}^{t}$ is the best solution found so far and $x_{r_{1}}^{t},x_{r_{2}}^{t},x_{r_{3}}^{t}$ are individuals drawn uniformly from the current population.

Cyclic population reduction: Rather than updating all N individuals in every iteration, CPO updates only the first $N_{t}$ of them, where $N_{t}$ follows the periodic schedule of Equation (2).

(2) $$N_{t}=N_{min}+\left(N-N_{min}\right)\left(1-\frac{tbmod(T/T_{c})}{T/T_{c}}\right)$$

with $T_{c}=2$ cycles. The reference implementation hard-codes an absolute floor of $N_{min}=120$, a value that is a reduction only for the much larger default population of the original experiments; with the population of thirty used here and throughout the comparable literature, an absolute floor of 120 would turn the reduction into growth, so in this study the floor is expressed as a proportion of the population, $N_{min}=0.8N$. Either way the schedule is periodic, and the working subset is the first $N_{t}$ individuals of the array, whatever their fitness.

First defence: Sight: The individual retreats from an imagined mid-point between itself and a randomly chosen conspecific, scaled by the distance to the incumbent best, as given by Equations (3) and (4).

(3) $$y_{i}^{t}=\frac{x_{i}^{t}+x_{r}^{t}}{2}$$

(4) $$x_{i}^{t+1}=x_{i}^{t}+\tau_{1}⊙\left|2\tau_{2}x_{CP}^{t}-y_{i}^{t}\right|$$

where $\tau_{1}$ is a standard normal vector and $\tau_{2}∼U(0,1)$. The normal multiplier gives the operator an unbounded step length, which is what makes it the most exploratory of the four.

Second defence: Sound: A binary mask decides, dimension by dimension, whether a coordinate is kept or replaced by a differential recombination of two random individuals, as given by Equations (5) and (6).

(5) $$U_{1,j}=\left\{\begin{array}{c} 1,\;r_{j}>r^{\prime }\;0, \end{array}\right.\;\mathrm{otherwise}\;j=1,2,\ldots ,D$$

(6) $$x_{i}^{t+1}=U_{1}⊙x_{i}^{t}+(1-U_{1})⊙\left[y_{i}^{t}+\tau_{3}\left(x_{r_{1}}^{t}-x_{r_{2}}^{t}\right)\right]$$

in which $r_{j}$ and $r^{\prime }$ are independent uniform variates and $\tau_{3}∼U(0,1)$. Because the mask is redrawn every iteration, the operator diversifies along a random subset of coordinates while leaving the remainder untouched.

Defence factor and odour diffusion: The two exploitation operators share a contraction schedule $\gamma_{t}$, a random sign vector $U_{2}$ and an individual-specific diffusion factor $S_{i}^{t}$, defined by Equations (7) and (9).

(7) $$\gamma_{t}=2\tau_{4}{\left(1-\frac{t}{T}\right)}^{t/T}$$

(8) $$U_{2,j}=\{+1,\;r_{j}<0.5-1,\;\mathrm{otherwise}$$

(9) $$S_{i}^{t}=exp\left(\frac{f(x_{i}^{t})}{\sum_{k=1}^{N_{t}}f(x_{k}^{t})+\varepsilon }\right)$$

where $\tau_{4}∼U(0,1)$ and ε is the machine epsilon. $\gamma_{t}$ decays from roughly 2 to 0 over the run, so the two exploitation operators contract as the budget is consumed, while $S_{i}^{t}$ scales the step by the individual’s share of the population’s total fitness. Note that the ordering this induces depends on the sign of the objective: when f is positive, as it is throughout the CEC2017 suite, poor individuals take longer steps than good ones, whereas on an objective that can take negative values—the childcare problem of Section 5.1 is one—the ordering reverses. The behaviour is inherited from the original algorithm and is left unchanged here so that the ablation of Section 4.3 isolates the three proposed strategies.

Third defence: Odour: The masked update of Equation (10) places a differential step around a randomly chosen base individual and subtracts the contracting term.

(10) $$x_{i}^{t+1}=(1-U_{1})⊙x_{i}^{t}+U_{1}⊙\left[x_{r_{1}}^{t}+S_{i}^{t}\left(x_{r_{2}}^{t}-x_{r_{3}}^{t}\right)-\tau_{3}U_{2}\gamma_{t}S_{i}^{t}\right]$$

Fourth defence: Physical attack: The charge is modelled as an inelastic collision between the porcupine and the predator. The collision force of Equation (11) enters the position update of Equation (12).

(11) $$F_{i}^{t}=\tau_{6}⊙S_{i}^{t}\left(x_{r_{1}}^{t}-x_{i}^{t}\right)$$

(12) $$x_{i}^{t+1}=x_{CP}^{t}+\left[\alpha (1-\tau_{5})+\tau_{5}\right]\left(U_{2}⊙x_{CP}^{t}-x_{i}^{t}\right)-\tau_{7}U_{2}\gamma_{t}F_{i}^{t}$$

where α=0.2 is the convergence-rate constant and $\tau_{5},\tau_{7}∼U(0,1)$. Every individual updated by this operator is pulled towards the single incumbent best $x_{CP}^{t}$.

Operator selection and acceptance: Which of the four operators an individual uses is decided by two nested coin flips, as in Equation (13), and the resulting candidate is accepted only if it does not worsen the individual, as in Equation (14).

(13) $$operator(i)=\left\{\begin{array}{c} \mathrm{sight},\;\;r_{a}<r_{b}\;\mathrm{and}\;r_{c}<r_{d}\\ \mathrm{sound},\;\;r_{a}<r_{b}\;\mathrm{and}\;r_{c}\geq r_{d}\\ \mathrm{odour},\;\;r_{a}\geq r_{b\;}\mathrm{and}\;r_{e}<T_{f}\\ \mathrm{attack},\;\;r_{a}\geq r_{b}\;\mathrm{and}\;r_{e}\geq T_{f} \end{array}\right.$$

(14) $$x_{i}^{t+1}=\left\{\begin{array}{c} x_{i}^{t+1},\;\;f(x_{i}^{t+1})\leq f(x_{i}^{t})\\ x_{i}^{t},\;\;\mathrm{otherwise} \end{array}\right.$$

with $r_{a},\ldots ,r_{e}∼U(0,1)$ and $T_{f}=0.8$. In expectation, the two exploration operators are applied to a quarter of the population each, the odour operator to two fifths and the attack operator to one tenth; these proportions are fixed for the whole run and do not respond to the landscape.

## 3. The Proposed Multi-Strategy Crested Porcupine Optimizer

Three properties of CPO limit it on the problems studied here. First, the initial population is drawn i.i.d. uniformly, so in the dimensions that matter for the applications of Section 5—between 20 and 630—a population of thirty points leaves large regions of the box unsampled purely by chance, and the quality of the whole run is hostage to that draw. Second, neither exploitation operator makes use of the fit individuals other than the best: the odour operator recombines around an individual drawn without regard to fitness, so selecting the guide has no fitness preference, while the attack operator pulls every individual it touches towards the one incumbent best, a single attractor that in PSO and DE has been associated with loss of diversity and premature convergence [29,30]. Third, the population schedule of Equation (2) is periodic, and it selects the working subset by array position rather than by fitness, so the evaluation budget is not preferentially spent on the individuals that are worth refining.

MSCPO addresses these three points with one strategy each, leaving the four defence mechanisms, the constants α=0.2 and $T_{f}=0.8$ and the greedy acceptance rule of Equation (14) exactly as published. Keeping the operator set fixed is deliberate: it makes the ablation of Section 4.3 a clean attribution of the gain to the three strategies rather than to an incidental reparameterisation of CPO.

### 3.1. Sobol Quasi-Opposition Initialisation

The comparative study of initialisation methods [28] motivates checking whether the starting design affects final quality for the algorithm and problem at hand. Sobol sampling has been used for initialisation in a recent snake optimiser [34] and in CPO variants [21,22,24]; these are precedents for coverage, not guarantees of improvement in high dimensions. OBL [35,36] contributes exact opposite candidates, while QOBL [37,38] contributes candidates between the midpoint and the opposite point. The targeted-defence CPO variant [25] also combines a structured initial sample with opposition. SQI differs by using quasi-opposites, applying them to all initial points and selecting the fittest population from the pooled candidates. Its evaluations are budgeted explicitly, and Section 4.3 tests whether this initialisation contributes after EGE and APR are present.

MSCPO combines the two ideas. A scrambled Sobol sequence $\{s_{1},\ldots ,s_{N}\}\subset [0,1{)}^{D}$ is generated and mapped into the search box by Equation (15).

(15) $$x_{i}=lb+s_{i}⊙(ub-lb),\;i=1,2,\ldots ,N$$

**Figure 2:**
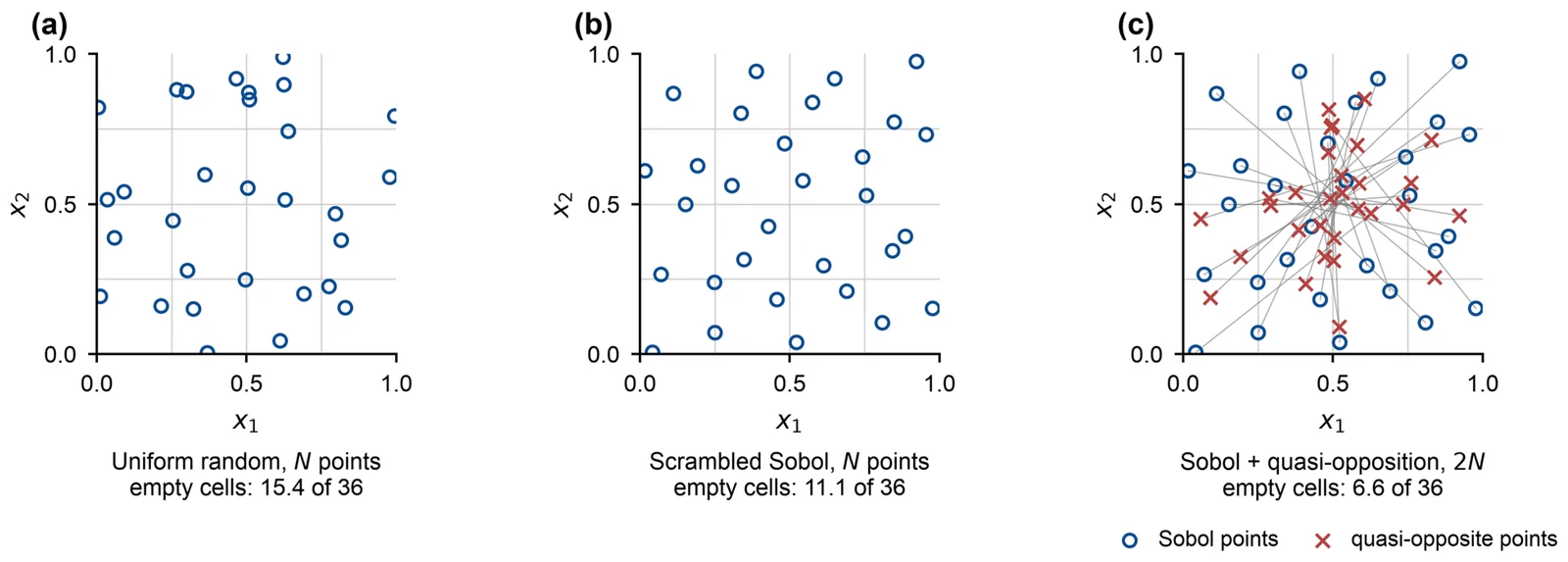
Effect of the initialisation strategy (N=30, D=2): (**a**) uniform sampling; (**b**) a scrambled Sobol sequence; (**c**) Sobol points with their quasi-opposites.

The low-discrepancy construction motivates more even initial coverage, particularly in low-dimensional projections. Scrambling permits independent repetitions. It does not guarantee a smaller covering radius or a better initial objective value for every finite sample, especially at high dimension; Figure 2 illustrates coverage in two dimensions, and Section 4.3 measures the contribution to final performance.

Each Sobol point is then paired with its quasi-opposite point. Writing M=(lb+ub)/2 for the centre of the box, the quasi-opposite of $x_{ij}$ is drawn uniformly on the segment joining the centre to the exact opposite point $lb_{j}+ub_{j}-x_{ij}$, as given by Equation (16).

(16) $${\overset{ˇ}{x}}_{ij}=M_{j}+r_{ij}\left(M_{j}-x_{ij}\right),\;r_{ij}\sim U(0,1)$$

Sampling between the centre and the opposite point rather than taking the opposite point itself is what distinguishes quasi-opposition from plain opposition; it keeps the extra sample away from the boundary, where the opposite of a boundary point would fall. The 2N candidates are evaluated once, and the initial population is formed by Equation (17).

(17) $$X^{0}=\underset{\left|S|=N,S\subseteq \{x_{i}\}\cup \{{\overset{ˇ}{x}}_{i}\right\}}{\arg\;\min}\sum_{x\in S}f(x)$$

that is, the N fittest of the 2N candidates. The strategy costs N additional FEs, which are charged against the same budget as every other evaluation, so the comparison with CPO remains exactly budget-matched. Figure 2 shows the effect in two dimensions. The counts under the panels are empty cells of a 6×6 grid averaged over 200 repetitions; a uniform sample paired with quasi-opposite points leaves 9.4 of them, so both components of the strategy contribute.

### 3.2. Elite-Guided Exploitation

EGE changes how CPO selects the guides in its exploitation phase [14]. The odour operator chooses a base individual uniformly, while the attack operator uses the incumbent best. Reports of premature convergence in SDHCPO [21] and HCPO [22] motivate investigating these choices, but do not identify their causal effect in CPO. Recent related methods address guidance in different ways. Diversity-guided PSO combines attraction and repulsion with fitness-dependent local learning [81]; diversity-enhanced DE uses diversity control and restart mechanisms [82]; and reference-point-guided multi-objective CPO uses archive-based guidance to balance multiple objectives [80]. MSCPO adopts none of these additional control mechanisms. It instead samples an elite guide within the existing single-objective exploitation equations, allowing the effect of guide selection to be isolated by ablation.

The closest source for the sampling rule is JADE [30], which selects the guide in current-to-pbest mutation from a top-ranked fraction of the population. EGE transfers this rule to CPO: it replaces the uniformly sampled base in the odour operator and the incumbent-best guide in the attack operator. It does not import DE’s difference-vector mutation, parameter adaptation or external archive. The contribution is therefore the placement and evaluation of elite-pool sampling within CPO, rather than the invention of elite sampling itself. Section 4.3 measures its effect while holding the defence constants and operator-selection rule fixed.

Let $X^{t}$ be the working population at iteration t, sorted by fitness. The elite pool is the fittest fraction p of it, as defined in Equation (18), and each individual draws its own guide uniformly from that pool, as in Equation (19).

(18) $$P^{t}=\left\{x_{\left(1\right)}^{t},x_{\left(2\right)}^{t},\ldots ,x_{\left(\lceil pN_{t}\rceil \right)}^{t}\right\},\;f\left(x_{\left(1\right)}^{t}\right)\leq \cdots \leq f\left(x_{\left(N_{t}\right)}^{t}\right)$$

(19) $$x_{pbest,i}^{t}∼U\left(P^{t}\right),\;i=1,2,\ldots ,N_{t}$$

The odour operator of Equation (10) then places its differential step around that guide instead of an arbitrary individual, giving Equation (20), and the attack operator of Equation (12) replaces the single incumbent best with the same guide, giving Equation (21).

(20) $$x_{i}^{t+1}=(1-U_{1})⊙x_{i}^{t}+U_{1}⊙\left[x_{pbest,i}^{t}+S_{i}^{t}\left(x_{r_{2}}^{t}-x_{r_{3}}^{t}\right)-\tau_{3}U_{2}\gamma_{t}S_{i}^{t}\right]$$

(21) $$x_{i}^{t+1}=x_{pbest,i}^{t}+\left[\alpha (1-\tau_{5})+\tau_{5}\right]\left(U_{2}⊙x_{pbest,i}^{t}-x_{i}^{t}\right)-\tau_{7}U_{2}\gamma_{t}F_{i}^{t}$$

The elite rate p interpolates between two extremes. As p falls, the pool shrinks towards its floor of two individuals, which is as close to CPO’s single attractor as the implementation allows; as p→1 the pool becomes the whole population, the selection pressure disappears, and the attack operator degenerates into an undirected recombination. Section 4.2 reports how the performance of MSCPO varies over the interval; all experiments in this paper use the default p=0.20.

### 3.3. Adaptive Population Reduction

Population size determines how a fixed FE budget is divided between more individuals and more generations. LPSR [11] provides the direct precedent for a monotone reduction with removal by fitness; CPO [14] instead repeatedly contracts and restores its working population. Recent DE research further distinguishes schedule shape from survivor selection. Archive-based DE combines nonlinear population reduction with adaptive archive use and rank-based selective pressure [83], whereas APR uses a linear schedule driven only by the consumed budget and fitness only to choose survivors. APR is consequently not a search-state feedback controller, unlike stage-dependent adaptation with diversity control [12]. These distinctions motivate testing the population floor separately in Section 4.3, rather than attributing any gain to monotonicity alone.

MSCPO replaces the periodic schedule of Equation (2) with the monotone linear rule of Equation (22).

(22) $$N_{t}=\left\lfloor N-\left(N-N_{min}\right)\frac{t}{T}\right\rceil ,\;N_{min}=\lceil \rho N\rceil$$

Whenever $N_{t}$ falls below the current population size, the surplus is removed by fitness, as in Equation (23), so the individuals that survive into the exploitation phase are the ones that have already proved useful.

(23) $$X^{t}\leftarrow \left\{x_{\left(1\right)}^{t},\ldots ,x_{\left(N_{t}\right)}^{t}\right\}$$

Two properties distinguish Equation (22) from Equation (2). It is monotone, so the average number of evaluations spent per surviving individual grows steadily and the search shifts from breadth to depth exactly once rather than twice; and it is fitness-aware, so the reduction is a selection step rather than an arbitrary slice of the array. The reduction ratio $\rho =N_{min}/N$ is the second free parameter of MSCPO; Section 4.2 reports its sensitivity, and all experiments in this paper use the default ρ=0.50. Like Equation (2), Equation (22) is a fixed function of the consumed budget and uses no feedback from the search; ‘adaptive’ refers to the adaptation of the population size to the stage of the run, and the two schedules differ in that Equation (22) is monotone and removes individuals by fitness. Section 4.3 shows, however, that at CPO’s floor of 0.8N these two properties bring no measurable gain; the contribution of APR comes from its lower floor, which is why the reduction ratio ρ matters (Section 4.2).

### 3.4. Implementation of MSCPO

**Figure 3:**
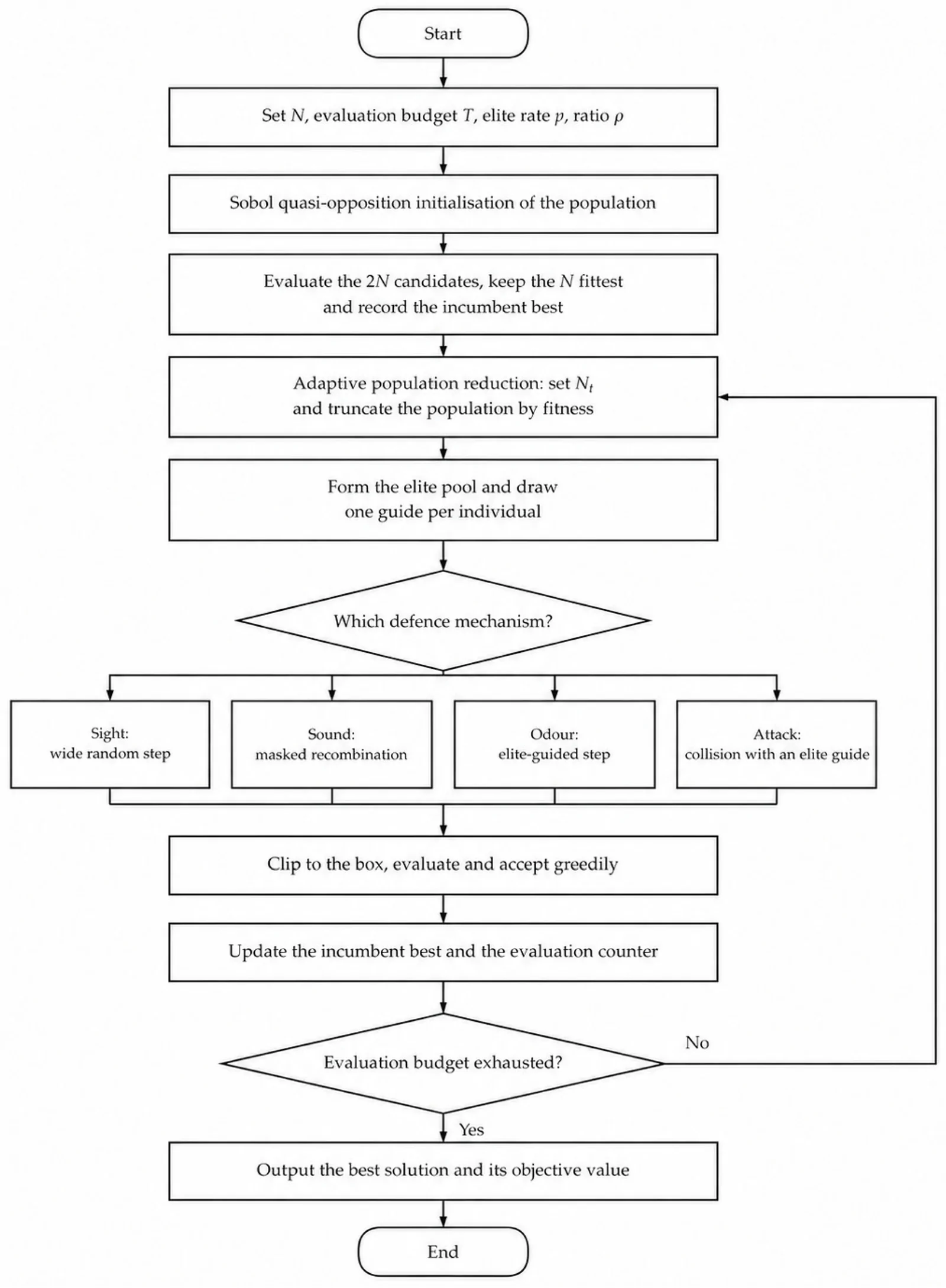
Flowchart of the proposed MSCPO.

Figure 3 gives the flow of MSCPO, and Algorithm 1 states it step by step. The algorithm has three free parameters beyond those inherited from CPO: the population size N, the elite rate p and the reduction ratio ρ.


**Algorithm 1:** The proposed MSCPO
1:**Input:** objective f, bounds lb,ub, population size N, evaluation budget T, elite rate p, reduction ratio ρ2:**Output:** best solution $x_{CP}$ and its value $f(x_{CP})$3:Generate N scrambled Sobol points and map them onto the box by Equation (15)4:Build the N quasi-opposite points by Equation (16)5:Evaluate the 2N candidates and keep the N fittest by Equation (17)//SQI, Section 3.16:Record the incumbent best $x_{CP}$ and set t←2N7:**while** t<T **do**8:     Set the working population size $N_{t}$ by Equation (22) and truncate by fitness with Equation (23)//APR, Section 3.39:     Compute $\gamma_{t}$, $U_{1}$, $U_{2}$ and $S_{i}^{t}$ by Equations (5) and (7)–(9)10:   Form the elite pool $P^{t}$ by Equation (18) and draw one guide $x_{pbest,i}^{t}$ per individual by Equation (19)//EGE, Section 3.211:   **for** i=1 **to** $N_{t}$ **do**12:   Select a defence mechanism for individual i by Equation (13)13:   **if** sight **then** generate the candidate by Equations (3) and (4)14:   **else if** sound **then** generate the candidate by Equations (5) and (6)15:   **else if** odour **then** generate the candidate by Equation (20)16:   **else** generate the candidate by Equation (21) with the collision force of Equation (11)17:   **end if**18:   Clip the candidate to [lb,ub], evaluate it and accept it by Equation (14)19:  **end for**20:  Update $x_{CP}$ and set $t\leftarrow t+N_{t}$21:
**end while**
22:**return** $x_{CP}$ and $f(x_{CP})$



### 3.5. Computational Complexity

Let N be the population size, D the dimension and T the evaluation budget, so that the number of iterations is $T/\bar{N}$ with $\bar{N}$ the mean working population size. CPO spends O(N) on initialisation, O(ND) per iteration on the position updates, and O(N) per iteration on the greedy acceptance, giving the total of Equation (24).

(24) $$O_{CPO}=O\left(N+\frac{T}{\bar{N}}ND\right)=O\left(TD\right)$$

MSCPO adds three terms. Generating a D-dimensional Sobol sequence of length N costs O(ND), and the quasi-opposite points cost a further O(ND), both once. Selecting the N fittest of 2N candidates costs O(NlogN) once. The elite pool and the truncation each require the population to be ordered, which costs O(NlogN) per iteration. The total is therefore given by Equation (25).

(25) $$O_{MSCPO}=O\left(ND+NlogN+\frac{T}{\bar{N}}\left(ND+NlogN\right)\right)=O\left(TD+TlogN\right)$$

Since logN≪D for every problem considered in this study, the asymptotic cost is O(TD), the same order as CPO. The additional ordering is performed on at most thirty elements per iteration and is negligible besides a single-objective evaluation in either of the two applications of Section 5. Moreover, because $N_{t}$ decreases monotonically under Equation (22), $\bar{N}<N$, so MSCPO performs more iterations than CPO for the same evaluation budget.

### 3.6. Design Rationale and Relation to Existing CPO Variants

The three strategies are not an arbitrary bundle. Each acts on a different, separable part of the search, which is what allows them to be combined without re-tuning one another and to be evaluated by a clean factorial design: SQI acts only on the information available before the first iteration, EGE only on the point towards which the two exploitation operators move an individual, and APR only on how the evaluation budget is divided between breadth and depth over the run. None of them alters the step-size logic of the four defence operators, the operator-selection rule of Equation (13) or the greedy acceptance of Equation (14).

The rationale for SQI is complementary coverage: Sobol sampling [34] and quasi-opposition [37] motivate the starting design, without guaranteeing better objective values. EGE introduces a fitness preference in selecting the odour guide and allows several elite individuals to replace the common attack guide. JADE [30] is the direct top-fraction sampling precedent; comprehensive-learning PSO [29] is a related use of multiple personal-best exemplars, while the recent diversity-guided PSO [81] also changes attraction, repulsion and local learning. MSCPO changes neither of those PSO update equations. APR follows the population-reduction principle of LPSR [11], but uses the larger floor specified in Section 3.3. Section 4.3 shows that retaining CPO’s floor gives no measurable improvement from the monotone, fitness-truncated schedule. The observed benefit is associated with the lower terminal population; the separate contribution of fitness truncation at that lower floor was not isolated.

The strategies are also coupled in one useful way. Because the elite pool is a fixed fraction of the current population, APR shrinks the pool as the run proceeds, from six guides at N=30 to three at $N_{min}=15$ with the default p=0.20, so the number of potential exploitation guides decreases with the consumed budget: early in the run guidance is spread over several good regions; late in the run it concentrates on the few individuals that survived truncation. SQI supplies the well-spread population on which this schedule starts. The coupling is weak, however, and the ablation of Section 4.3 shows that the combination is additive rather than synergistic.

Table 1 sets MSCPO beside the base algorithm and the enhanced CPO variants for which a published description was available. Three differences stand out. First, MSCPO introduces no new search operator: all four defence equations are retained and only the guide of Equations (10) and (12) and the population schedule change, whereas CAPCPO, ECPO, SDHCPO and the targeted-defence variant [25] replace or augment two or three of the defence equations with Cauchy, Levy, differential or crossover steps, and BTCPO [27] adds a further update step before every CPO iteration. Second, none of the variants whose update rules could be read guides exploitation by an elite pool: SDHCPO and BTCPO keep the incumbent best in the attack operator, CAPCPO and ECPO extend its pull to further operators, and the targeted-defence variant [25] replaces it with a random peer; the abstract of HCPO describes a cellular-automata neighbourhood that regulates the diffusion of elite information [22], the closest idea, but its form could not be checked. Third, the variants either keep the cyclic population reduction [21,24,25,27], delete it [19,20], replace it with a switch between N and 0.5N triggered by the diversity of the fitness values [23], or, in two variants listed by the systematic review, adjust the population size in a form the review does not describe [20]. We found no report of a monotone, fitness-truncated schedule of the LPSR type in CPO; the targeted-defence study [25] proposes one as future work after suggesting the cyclic schedule as a plausible cause of their variant’s weaker results at D=100. The initialisation, by contrast, is not a point of difference: Sobol and opposition-based starts are already used in [21,22,24,25]. SQI differs from them in drawing quasi-opposite rather than opposite partners, in applying opposition to every point rather than to the worse half [21], and in keeping the N fittest of the pooled 2N candidates rather than the better of each pair [21,25]. It is retained because it completes a design in which each stage of the search is addressed once, although Section 4.3 shows that it is the weakest of the three strategies. Finally, the label ‘multi-strategy CPO’ is generic and has been used for several unrelated variants, including the targeted-defence variant [25] and others listed in the systematic review [20]; the algorithm proposed here is the one defined by Section 3.1, Section 3.2 and Section 3.3 and Algorithm 1.

## 4. Numerical Experiments on the CEC2017 Suite

This section establishes that the three strategies of Section 3 do what they are supposed to do. Section 4.1 fixes the protocol, Section 4.2 examines the sensitivity to the two free parameters, Section 4.3 isolates the contribution of each strategy by a full factorial ablation, Section 4.4 compares MSCPO with nine competing algorithms, and Section 4.5 examines the exploration–exploitation behaviour that the comparison leaves implicit. Section 4.6 reports the computational cost, Section 4.7 repeats the comparison at the full CEC2017 budget, and Section 4.8 compares the robustness of the ten algorithms.

### 4.1. Experimental Setting

All experiments were run on a workstation with 16 CPU cores and 64 GB of memory under macOS 15.8, in Python 3.13.15 with NumPy 2.5.3 and SciPy 1.18.1. The CEC2017 benchmark suite is used in full: 29 functions, of which F1 and F3 are unimodal, F4–F10 are simple multimodal, F11–F20 are hybrid and F21–F30 are composition functions; the withdrawn F2 is excluded, as is now standard. The search domain is $[-100,100{]}^{D}$ and the dimension is set to 10, 30, 50 and 100, the four dimensions defined for the CEC2017 suite. Together they cover low-, medium- and high-dimensional search: the childcare instances of Section 5, with 20 to 100 decision variables, lie inside this range, whereas three of the four scheduling instances, with 224 to 630 variables, extend beyond it, which is one reason why the scheduling results are reported in their own right rather than inferred from the benchmark. Every algorithm uses a population of N=30 and the same budget of 30,000 FEs, which corresponds to 1000 iterations of an algorithm that evaluates its population once per iteration. The budget, not the iteration count, is the stopping criterion: a run terminates when exactly 30,000 FEs have been consumed; no tolerance-based or stagnation-based termination is used, and the evaluations spent on initialisation, including the *N* extra evaluations of SQI, are charged to the same budget. Algorithms that evaluate their population twice per iteration (SBOA, BKA and RBMO) or a variable number of times (HHO) therefore complete fewer iterations, and every time-dependent parameter is driven by the consumed share of the budget. Each algorithm is run 30 times independently on every function and dimension, each run with its own random seed. Thirty runs is the sample size used by the CPO variants compared in Section 3.6 [21,23,24,25], whereas the CEC2017 competition prescribes 51; a simulation with normally distributed errors shows that, with 30 runs per sample, the two-sided Wilcoxon rank-sum test used below detects a shift of 0.8 standard deviations at the 0.05 level with a probability of 0.84, while the Friedman tests use the functions, not the runs, as blocks. The reported quantity is the error $f(x_{best})-f^{*}$ with respect to the known optimum of the function. Every algorithm is driven through the same evaluation wrapper, which counts evaluations, applies the same box clipping and stops the run at exactly the same budget, so no algorithm can gain an advantage from a different accounting of its evaluations. Across all 34,800 runs, no non-finite result occurred.

Apart from the number of independent runs (30 here against 51, discussed above), two aspects of the protocol depart from the CEC2017 technical report and are stated here rather than buried. The first is the budget. The report specifies 10,000×D evaluations, which at D=100 is a million; the protocol used here is close to that of the CPO variant literature, a population of thirty and 500 to 1000 generations [21,23,24,25], and it is 30 per cent of the standard budget at D=10 and 3 per cent of it at D=100. The choice is deliberate: it targets the regime in which evaluations are scarce, such as siting problems at city scale, rosters for large workforces and policy analyses that re-solve a model many times, as the recalibration and the budget sweep of Section 5.1.4 do. The instances of Section 5 are small enough for the full budget to be affordable, so they illustrate this regime rather than require it. It is, however, a short-budget regime, and short budgets favour algorithms that converge quickly. Section 4.7 therefore repeats the comparison at the full 10,000×D budget and reports what changes. The second is the implementation of the suite itself: the widely used reference implementation of CEC2017 in the opfunu package transcribes the Modified Schwefel component of the official C source with two sign errors, which allow every function containing that component—twelve of the twenty-nine—to be driven arbitrarily far below its declared optimum, and it returns NaN for F18 and F30 at D=10 because a one-dimensional index group divides by zero. Both are corrected here against the official source; the corrected implementation is available from the corresponding author on request, together with a script that verifies agreement with opfunu to $10^{-9}$ on every other function and dimension. All ten algorithms are run on the corrected suite, so the comparison is internally consistent, but absolute error values are not comparable with papers that use the uncorrected package on those twelve functions.

MSCPO is compared with its own base algorithm CPO and with eight further population-based optimisers introduced in Section 1: SBOA, BKA, RBMO, DBO, HHO, WOA, GWO and PSO. The set spans three decades of the field and four levels of maturity, from the canonical PSO to three optimisers published in 2024. Every control parameter is taken from the source publication without retuning, as listed in Table 2; only the shared population size and evaluation budget are standardised. Table 2 also gives the population size, the initialisation procedure and the stopping criterion of every algorithm, together with the settings common to all of them.

The statistical analysis follows established non-parametric procedures for comparing evolutionary and swarm algorithms [77] and uses a significance level of α=0.05 throughout. Differences among k algorithms or variants over n blocks, a block being one function at one dimension (n=29 per dimension, n=116 when the four dimensions are pooled), are first tested with the Friedman test on the mean errors. Its statistic $\chi_{F}^{2}$ has k−1 degrees of freedom, and the Iman–Davenport statistic $F_{ID}$ derived from it has (k−1,(k−1)(n−1)) degrees of freedom. When equivalence is rejected, two post hoc procedures follow. The Nemenyi test compares all pairs of algorithms and declares two of them different when their mean ranks differ by more than the critical difference $CD=q_{\alpha }\sqrt{k(k+1)/(6n)}$; Holm’s step-down procedure compares every algorithm with MSCPO as the control, using $z=({\bar{R}}_{j}-{\bar{R}}_{MSCPO})/\sqrt{k(k+1)/(6n)}$ and adjusting the two-sided *p*-values for the number of comparisons. Differences on individual functions are tested with the two-sided Wilcoxon rank-sum test on the 30 final errors. These function-level p-values are not adjusted for multiplicity; Section 4.4 reports how a Holm adjustment changes the comparison with CPO. The degrees of freedom of every Friedman test are given in the note to the corresponding table.

### 4.2. Parameter Sensitivity Analysis

**Figure 4:**
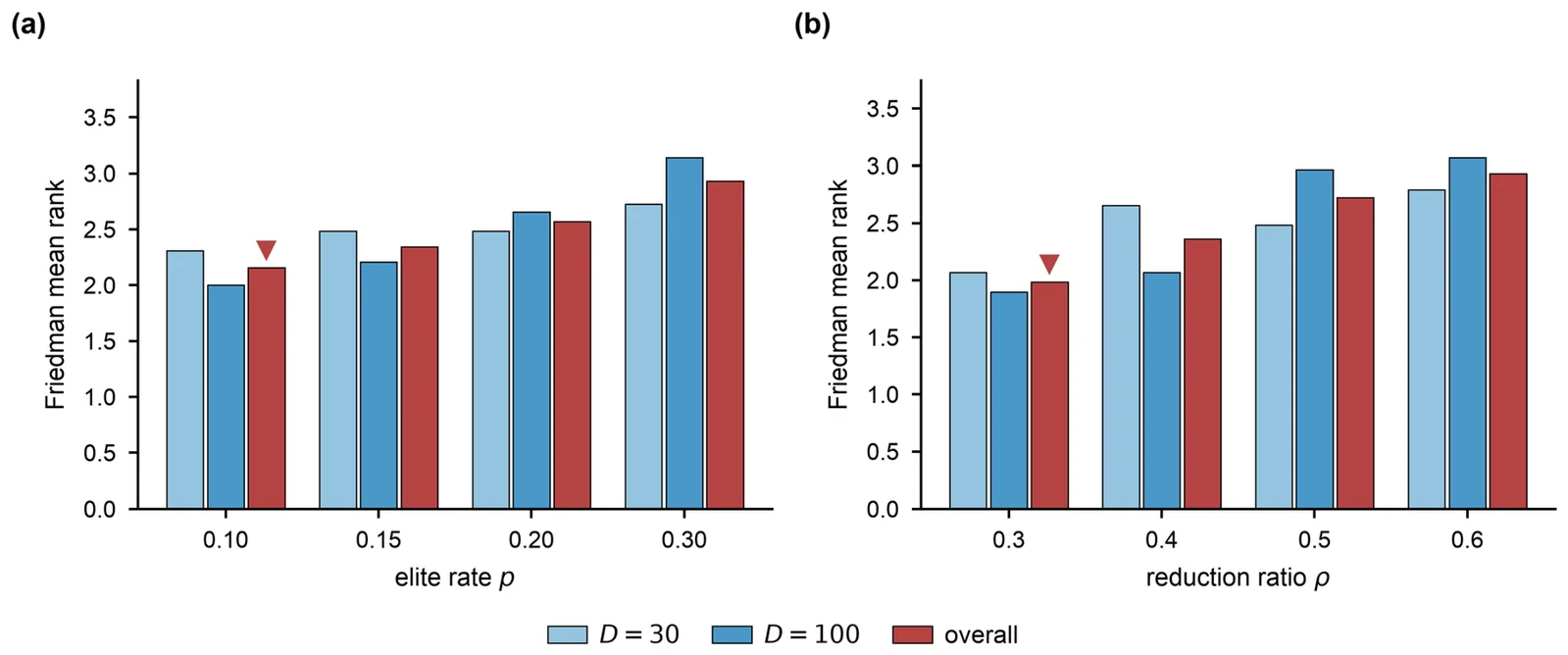
Friedman mean ranks of MSCPO on the CEC2017 suite (α=0.05): (**a**) elite rate p; (**b**) reduction ratio ρ. The inverted red triangles mark the setting with the lowest overall Friedman mean rank (p=0.10 in (**a**); ρ=0.3 in (**b**)); they are not significance markers.

MSCPO adds two free parameters to CPO: the elite rate p of Equation (18) and the reduction ratio $\rho =N_{min}/N$ of Equation (22). Each is varied over four values while the other is held at its default, on the complete suite at D=30 and D=100 with 30 independent runs, and the settings are ranked by the Friedman test at the 0.05 level. Throughout, a lower Friedman mean rank denotes better performance. Table 3 and Table 4 report the mean ranks, and Figure 4 summarises both grids.

The elite rate governs how much selection pressure the two exploitation operators carry. Very small values reproduce CPO’s single-attractor behaviour and very large values dissolve the pressure altogether. Over the grid tested, the mean rank rises monotonically with p, from 2.16 at p=0.10 to 2.93 at the largest value, and the Friedman test over the four settings is significant ($\chi_{F}^{2}(3)=11.61$, p = 0.009). The reduction ratio governs how fast the evaluation budget is concentrated on the survivors; here too the ordering is monotone, from 1.98 at ρ=0.3 to 2.93 at the largest value ($\chi_{F}^{2}(3)=18.19$, p < 0.001).

Two things should be said about these grids. The first is that neither profile is steep. The spread between the best and the worst setting is 0.78 rank units for p and 0.95 for ρ on a four-point scale, against a spread of 8.58 units between the best and the worst algorithm on the ten-point scale of Section 4.4, so the performance of MSCPO is governed by the three strategies rather than by the calibration of their parameters. The second is that every experiment reported in this paper—the ablation of Section 4.3, the comparison of Section 4.4 and both applications of Section 5—uses the default setting p=0.20 and ρ=0.50, which is not the optimum of either grid. Tuning the proposed algorithm on the same suite that is then used to evaluate it would inflate the comparison; keeping the defaults makes every number reported below a conservative estimate of what MSCPO can do. Practitioners with a specific problem class should of course tune, and Table 3 and Table 4 indicate the direction in which to do so.

### 4.3. Ablation Study

**Figure 5:**
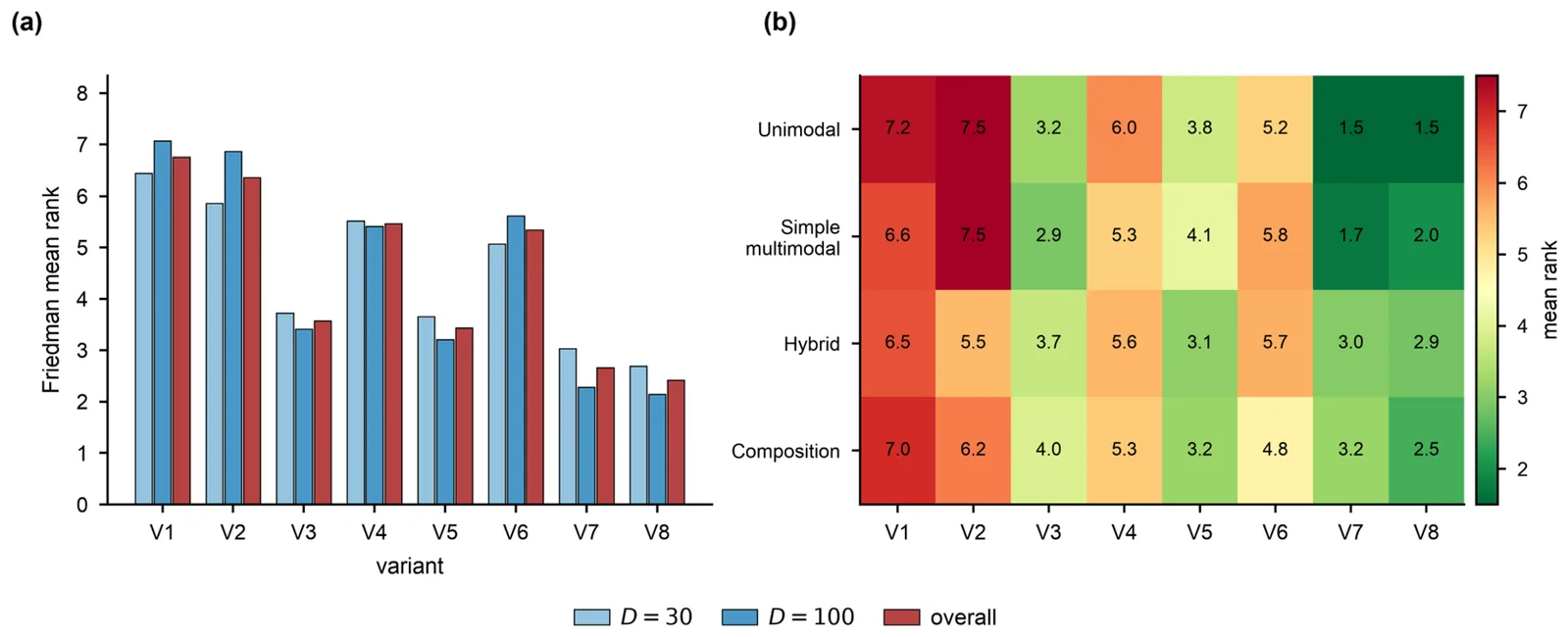
Friedman mean ranks of the ablation variants on the CEC2017 suite (α=0.05): (**a**) by dimension and overall; (**b**) heatmap by function group.

Because MSCPO leaves CPO’s operators untouched, the three strategies can be switched on and off independently, and the eight resulting variants of Table 5 form a complete $2^{3}$ factorial design. V1 is CPO with the harness of this study; V8 is MSCPO. Table 6 gives the Friedman ranks and Figure 5 shows them graphically.

Because the design is a complete factorial, the contribution of each strategy can be read off as a main effect rather than inferred from a ranking. On the mean Friedman rank, where a negative effect is an improvement, the main effects are −2.97 for EGE, −1.06 for APR and −0.22 for SQI. Every strategy helps; every single-strategy variant ranks ahead of V1 (6.36, 3.57 and 5.47 against 6.76), and the full combination V8 attains 2.41, the best of the eight, although the post hoc tests do not separate it from V7 (Holm-adjusted p=0.60, note to Table 6), so SQI adds no significant improvement once EGE and APR are present. But the three contributions are of very different sizes: EGE accounts for about 70 per cent of the total main effect, APR for 25 per cent and SQI for only 5 per cent. The diagnosis of Section 3 is therefore confirmed strongly for the exploitation phase and only weakly for the initialisation, and for the population schedule only in the narrower form established below: among the three interventions tested, elite-pool guidance has the largest main effect under the present benchmark protocol. This does not compare EGE with untested operator replacements.

The factorial also shows what the three strategies do not do, which is interact. All three two-factor interactions and the three-way interaction are at most 0.09 rank units, an order of magnitude below the EGE main effect and smaller than the run-to-run noise in the ranks. The effects are therefore additive: combining the strategies is worthwhile because their benefits sum, not because they reinforce one another. That is a weaker claim than synergy, and it is the one the data support. It also implies that a practitioner who can afford only one modification should implement EGE, and that SQI—which costs N extra evaluations for a main effect of 0.22 rank units—is the first thing to drop on a problem where evaluations are expensive. The Friedman test over the eight variants rejects equivalence ($\chi_{F}^{2}(7)=193.14$, p < 0.001).

Because APR changes both the shape of the schedule and its floor, from 0.8N in CPO to 0.5N, a further variant separates the two: APR with CPO’s floor, ρ=0.8, run under the same protocol (30 runs at D=30 and D=100). Its Friedman mean rank is 2.31, against 2.14 for V1 and 1.55 for V4 ($\chi_{F}^{2}(2)=18.34$, p<0.001, n=58 blocks). Holm’s procedure with V1 as the control separates V4 (adjusted p=0.003) but not the variant with CPO’s floor (p=0.35), and the rank-sum tests give that variant 2 wins, 54 ties and two losses against V1 over the 58 function–dimension pairs; at D=30 none of the three differs significantly. The monotone, fitness-truncated form of the schedule therefore brings no measurable gain on its own, and the contribution of APR comes from the smaller population it reaches late in the run, which buys more iterations for the survivors and agrees with Table 4, where the overall mean rank improves monotonically as the floor is lowered. The third point of the diagnosis of Section 1 is thus confirmed only in this narrower form; whether truncation by fitness matters once the floor is low was not tested.

### 4.4. Comparison with State-of-the-Art Algorithms

The mean and standard deviation of the error of every algorithm on every function at each of the four dimensions are reported in Appendix A as Table A1, Table A2, Table A3 and Table A4. MSCPO attains the lowest mean error on 85 of the 116 function–dimension pairs, ahead of CPO with 19.

**Figure 6:**
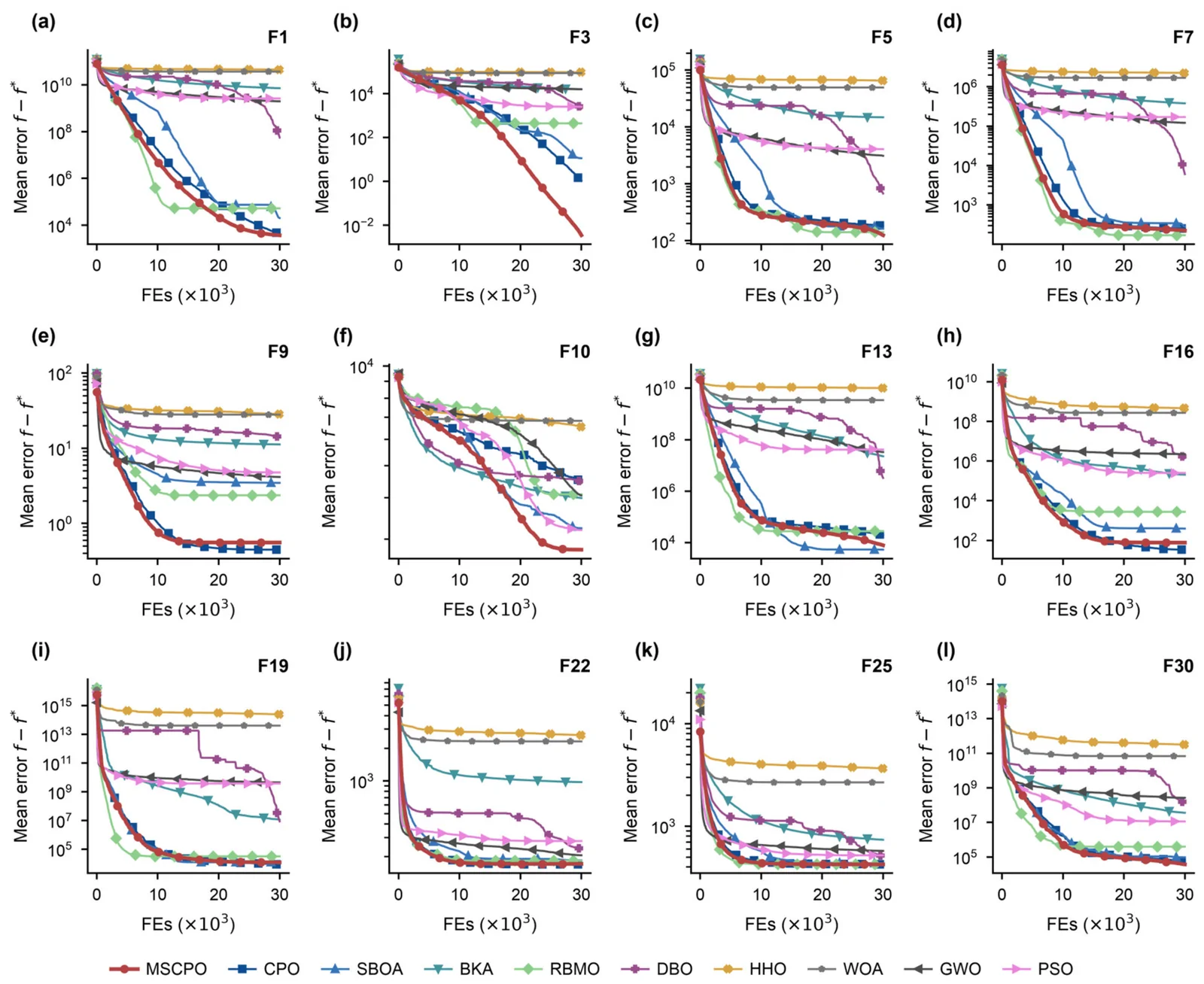
Convergence curves of MSCPO and the comparison algorithms on twelve representative CEC2017 functions (D=30): (**a**) F1; (**b**) F3; (**c**) F5; (**d**) F7; (**e**) F9; (**f**) F10; (**g**) F13; (**h**) F16; (**i**) F19; (**j**) F22; (**k**) F25; (**l**) F30. The superscript asterisk in $f^{*}$ denotes the known optimal objective value; star-shaped curve markers identify WOA.

**Figure 7:**
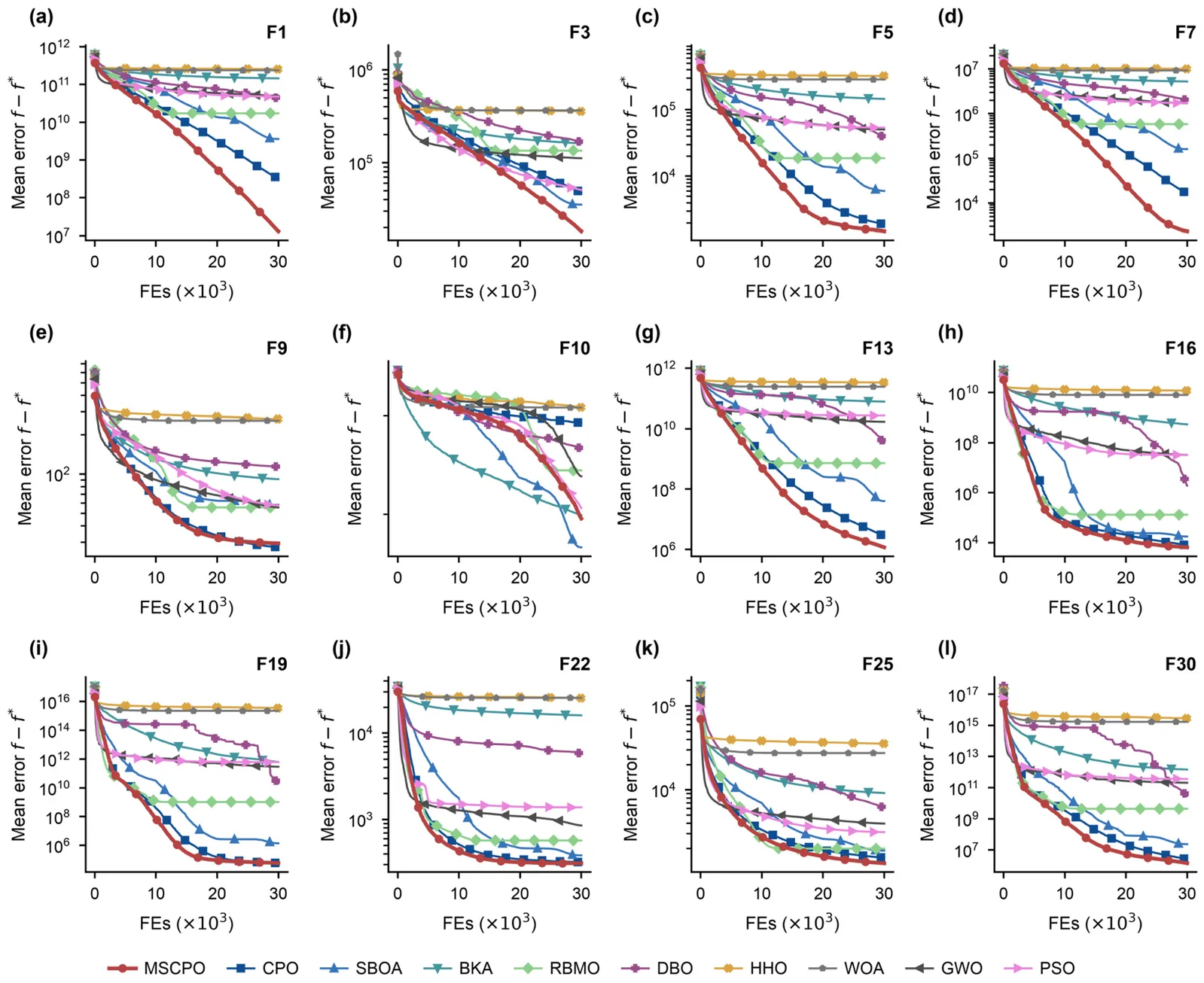
Convergence curves of MSCPO and the comparison algorithms on the same twelve CEC2017 functions (D=100): (**a**) F1; (**b**) F3; (**c**) F5; (**d**) F7; (**e**) F9; (**f**) F10; (**g**) F13; (**h**) F16; (**i**) F19; (**j**) F22; (**k**) F25; (**l**) F30. The superscript asterisk in $f^{*}$ denotes the known optimal objective value; star-shaped curve markers identify WOA.

**Figure 8:**
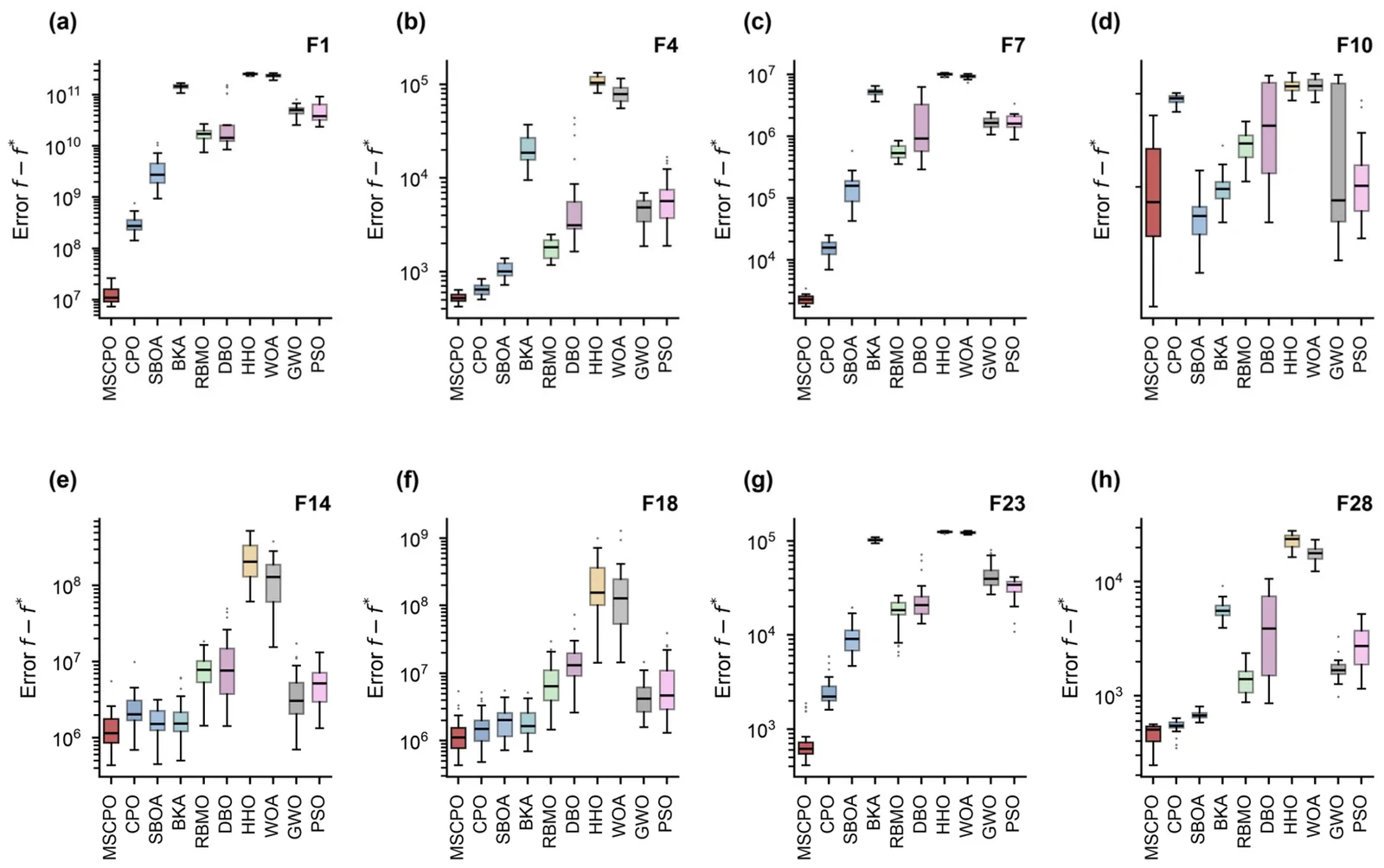
Distribution of the final error of MSCPO and the comparison algorithms on eight representative CEC2017 functions (D=100): (**a**) F1; (**b**) F4; (**c**) F7; (**d**) F10; (**e**) F14; (**f**) F18; (**g**) F23; (**h**) F28. The superscript asterisk in $f^{*}$ denotes the known optimal objective value; outlying runs are shown as dots.

**Figure 9:**
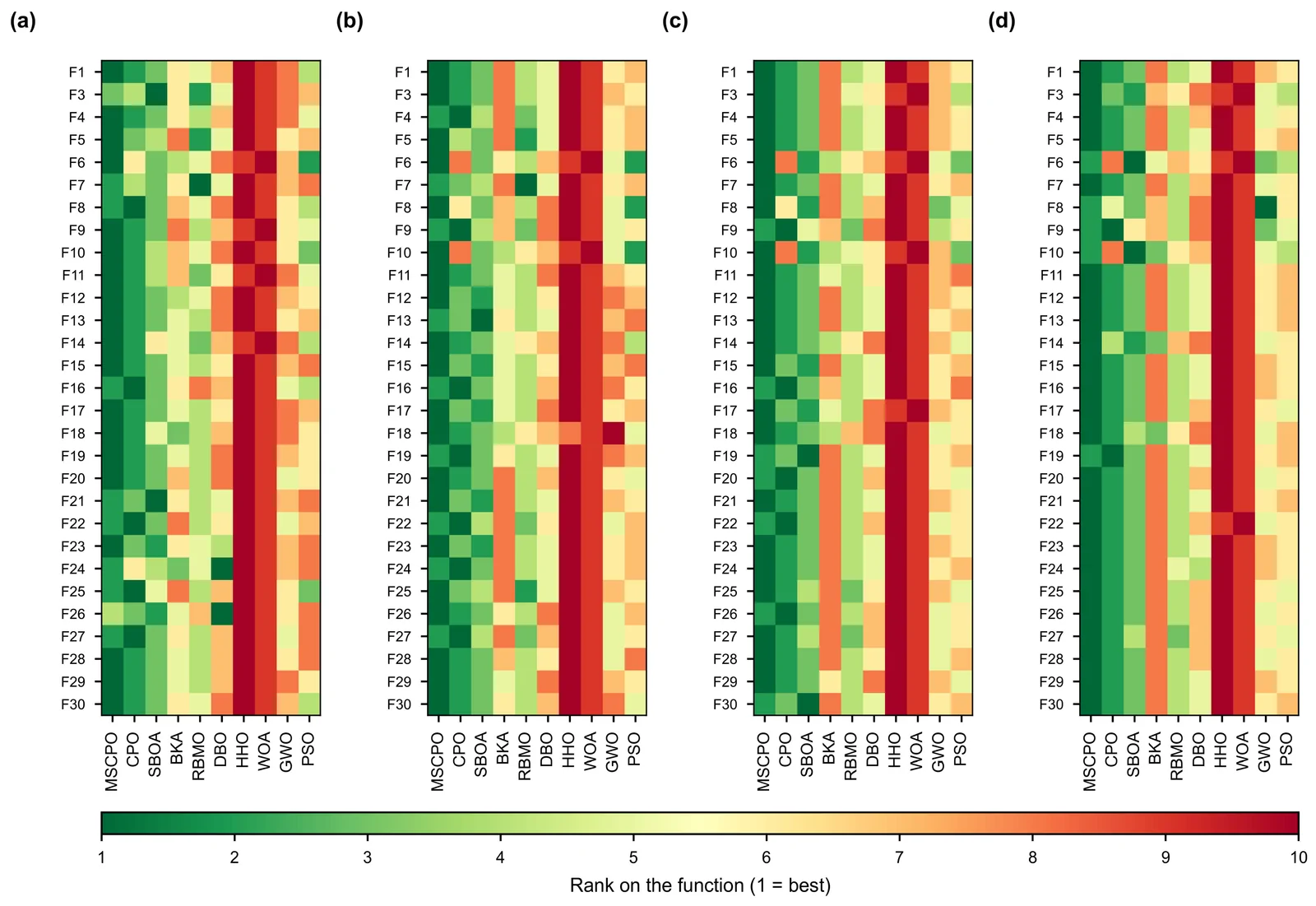
Ranking heatmaps of MSCPO and the comparison algorithms on the CEC2017 suite: (**a**) D=10; (**b**) D=30; (**c**) D=50; (**d**) D=100.

The behaviour behind those numbers is shown in Figure 6 and Figure 7, which give the mean convergence curves at D=30 and D=100; in Figure 8, which gives the dispersion over the thirty runs; and in Figure 9, which gives the rank of every algorithm on every function. The functions shown cover the four CEC2017 groups: Figure 6 and Figure 7 show two unimodal, four simple multimodal, three hybrid and three composition functions (F1, F3; F5, F7, F9, F10; F13, F16, F19; F22, F25, F30) and Figure 8 shows one, three, two and two (F1; F4, F7, F10; F14, F18; F23, F28). The selection does not favour MSCPO: at D=30 it finishes first on six of the twelve functions of Figure 6, a smaller share than on the full suite, where it has the lowest mean error on 20 of the 29. The boxes of Figure 8 give the interquartile range, the whiskers reach the most extreme run within 1.5 times that range, and the dots are the runs beyond them.

The margin widens with dimension. MSCPO attains the lowest mean error on 19 of the 29 functions at D=10 and on 24 of them at D=100, and the median ratio of CPO’s mean error to MSCPO’s rises from 1.27 at D=10 to 1.49 at D=100; the geometric mean of that ratio at D=100 is 2.00. That direction is the opposite of what a purely exploitative modification would produce, and it is the behaviour the three strategies were designed for: the value of a well-covered initial sample, of a pool of guides rather than a single one, and of concentrating the budget on survivors all grow with the volume of the search space. MSCPO also leads in every function group, with mean ranks of 1.25 on the unimodal functions, 1.36 on the simple multimodal ones, 1.20 on the hybrid ones and 1.35 on the composition ones, against 2.38, 3.86, 2.07 and 2.08 for CPO. The largest margin over CPO is on the simple multimodal group, where the gap is 2.50 rank units against 0.87 on the hybrid group and 0.73 on the composition group. That is where a population collapsed onto a single attractor is punished hardest: a simple multimodal landscape has many separated basins of comparable quality, so an algorithm that keeps several good guides alive finds a better one, whereas the hybrid and composition landscapes are hard for every algorithm and compress the differences between them. The convergence curves of Figure 6 and Figure 7, read below, show when this advantage arises. The dispersion of the results tells the same story: the median ratio of MSCPO’s standard deviation to CPO’s over all function–dimension pairs is 0.82, so the proposed algorithm is not only better on average but more reliable from run to run, which Figure 8 confirms.

Figure 6 and Figure 7 show how the final ranks are reached. At D=30 (Figure 6), MSCPO finishes first on six of the twelve functions shown (F1, F3, F5, F10, F25 and F30) and second on the other six, behind CPO on F9, F16, F19 and F22, RBMO on F7 and SBOA on F13. On all twelve, its mean error is already below CPO’s after a fifth of the budget, by factors between 1.03 and 2.41. What happens afterwards depends on the function. On F3 and F10 the gap keeps widening to the end, most dramatically on F3, where the ratio of CPO’s mean error to MSCPO’s grows from 1.3 at a fifth of the budget to about 300. On F9, F16, F19 and, marginally, F22, by contrast, CPO overtakes MSCPO around or after the middle of the run, at about 46, 62, 45 and 51 per cent of the budget respectively, and the ratio falls from 1.26, 2.41, 2.13 and 1.04 at a fifth of the budget to 0.80, 0.44, 0.73 and 0.99 at the end. These four functions show in miniature the short-budget character of MSCPO that Section 4.7 establishes on the whole suite: the smaller late population to which Section 4.3 traces part of MSCPO’s lead may leave it less to work with late in the run. At D=100 (Figure 7), the lead is both larger and more durable. MSCPO finishes first on nine of the twelve functions, and second on the remaining three (F9 and F19 behind CPO, F10 behind SBOA), and on seven of them (F1, F5, F7, F13, F16, F22 and F30) it becomes the best of the ten algorithms within the first fifth of the budget and remains so to the end. On the unimodal F1 and the simple multimodal F7, the final mean errors of CPO are 24 and 7 times those of MSCPO, whereas on the composition functions F22 and F25 all curves flatten within the first half of the budget and MSCPO ends only 3 and 14 per cent below CPO, which is why the composition group compresses the ranking by function group.

Figure 8 turns from averages to distributions at D=100. MSCPO has the lowest median error on seven of the eight functions shown; the exception is F10, the shifted and rotated Schwefel function, on which SBOA has the lower median and also the lower mean (Table A4). On F1, F7 and F23, the whole interquartile box of MSCPO lies below the boxes of all nine competitors, showing separation of the central 50% of the run distributions. This does not establish dominance in every individual run. On F4 and F28, its box overlaps that of CPO, and on F14 and F18 it overlaps those of CPO, SBOA and BKA, so MSCPO is better on average but not in every run. Its interquartile range is narrower than CPO’s on six of the eight functions; it produces at most five outlying runs on any function, and none on four of them, and its thirty final errors span less than one order of magnitude on six functions and 1.1 orders on F14 and F18. F10 is the function on which MSCPO is clearly less consistent than CPO (on F28 its box is also about twice as wide as CPO’s): its interquartile range is about nine times CPO’s, because some of its runs reach a much better basin than others, whereas CPO’s runs cluster tightly at a higher error.

Figure 9 shows that the lead of MSCPO is broad rather than carried by a few functions. MSCPO ranks first on 19, 20, 22 and 24 of the 29 functions at D=10, 30, 50 and 100 and second on eight, nine, seven and five; it falls below second place only twice, both at D=10 (third on F3 and fourth on F26). Of the 31 function–dimension pairs on which it is not first, the winner is CPO on 19, SBOA on seven, DBO and RBMO on two each and GWO on one. The bottom of every panel is equally stable: HHO and WOA occupy the last two ranks on every function at every dimension, with a single exception at D=30, where GWO displaces HHO from the last two. The middle of the field is where dimension matters. GWO improves with dimension, above all on the simple multimodal functions (mean rank 6.57 at D=10 against 4.00 at D=100), whereas BKA deteriorates (mean rank over all functions 5.79 at D=10 against 7.28 at D=100), which is why the two exchange positions in Table 7.

**Figure 10:**
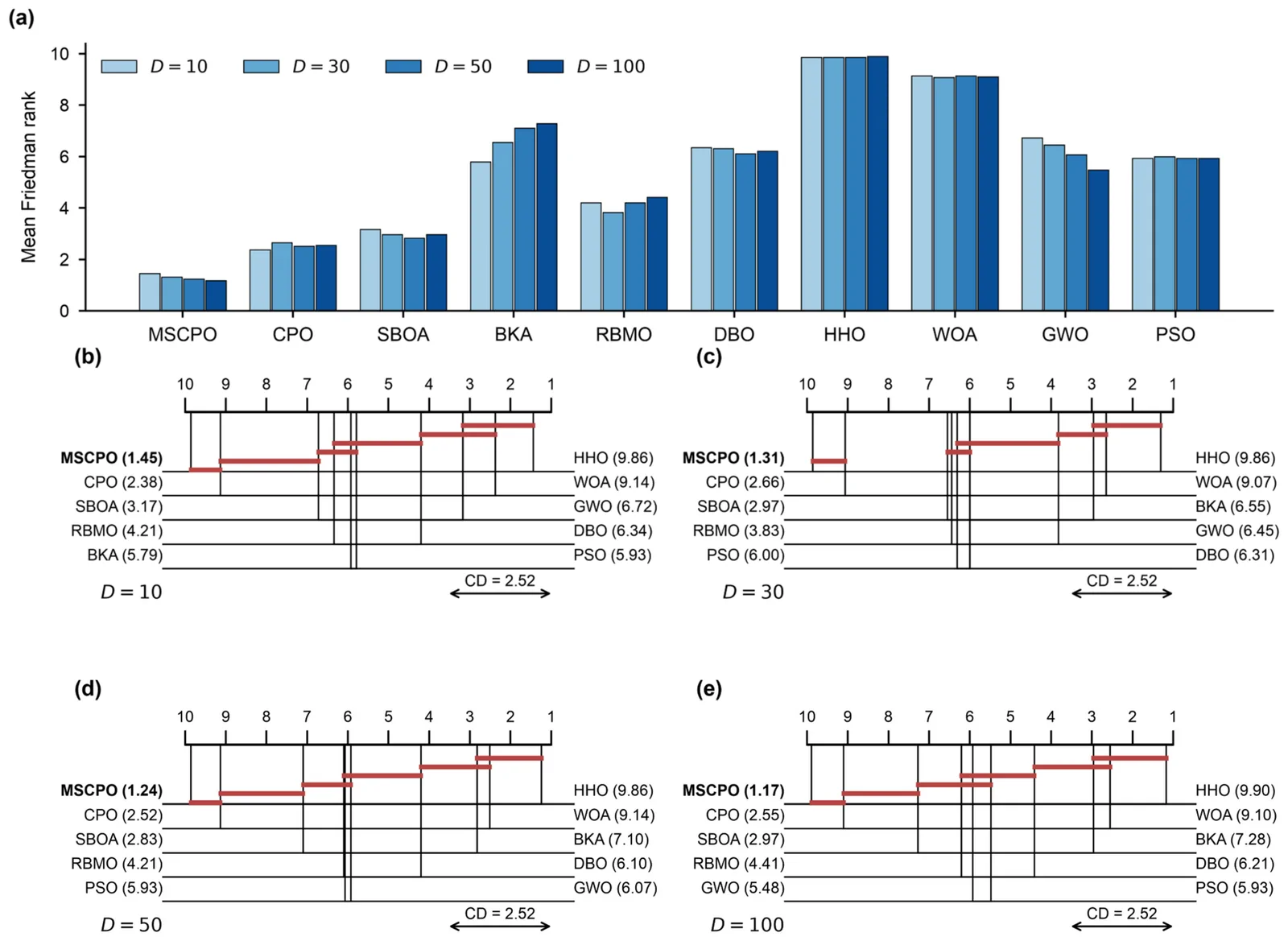
Friedman ranks and Nemenyi post hoc test of MSCPO and the comparison algorithms (α=0.05): (**a**) mean ranks by dimension; (**b**) D=10; (**c**) D=30; (**d**) D=50; (**e**) D=100. Numbers 1–10 on the rank axes are mean-rank positions (lower is better); red horizontal segments connect algorithms whose mean ranks are not separated by the Nemenyi critical difference at α=0.05.

The Friedman test rejects the hypothesis that the ten algorithms are equivalent at every dimension and on the pooled data. Pooled over the 116 function–dimension blocks, $\chi_{F}^{2}(9)=878.54$ and the Iman–Davenport statistic $F_{ID}(9,1035)=610.60$, both with p<0.001; at each single dimension, with 29 blocks, $\chi_{F}^{2}(9)=215.10$, 220.01, 226.37 and 225.18 and $F_{ID}(9,252)=131.21$, 150.30, 183.03 and 176.02 for D=10, 30, 50 and 100, all with p<0.001. MSCPO has the lowest mean rank at every dimension, and the rank falls monotonically from 1.45 at D=10 to 1.17 at D=100. Table 7 collects the mean ranks, Table 8 breaks them down by function group, and Figure 10 shows them together with the Nemenyi diagrams; the statistics for each dimension and the post hoc comparisons are given in Table 9. Algorithms joined by a bar in a Nemenyi diagram are not significantly different at the 0.05 level.

**Table 7:** Friedman mean ranks of MSCPO and the comparison algorithms on the CEC2017 suite (α=0.05).

Algorithm	D = 10	D = 30	D = 50	D = 100	Overall	Final Rank
**MSCPO**	**1.45**	**1.31**	**1.24**	**1.17**	**1.29**	**1**
CPO	2.38	2.66	2.52	2.55	2.53	2
SBOA	3.17	2.97	2.83	2.97	2.98	3
BKA	5.79	6.55	7.10	7.28	6.68	8
RBMO	4.21	3.83	4.21	4.41	4.16	4
DBO	6.34	6.31	6.10	6.21	6.24	7
HHO	9.86	9.86	9.86	9.90	9.87	10
WOA	9.14	9.07	9.14	9.10	9.11	9
GWO	6.72	6.45	6.07	5.48	6.18	6
PSO	5.93	6.00	5.93	5.93	5.95	5

Friedman test over all functions and dimensions (n=116 blocks, k=10 algorithms): $\chi_{F}^{2}(9)=878.54$, p<0.001; Iman–Davenport $F_{ID}(9,1035)=610.60$, p<0.001. Nemenyi critical difference at the 0.05 level: 1.258 (2.516 at a single dimension, n=29). The statistics for each dimension and the post hoc comparisons are given in Table 9. The row of the best algorithm is in bold.

**Table 8:** Friedman mean ranks of MSCPO and the comparison algorithms within the four CEC2017 function groups.

Algorithm	Unimodal	Simple Multimodal	Hybrid	Composition
MSCPO	**1.25**	**1.36**	**1.20**	**1.35**
CPO	2.38	3.86	2.07	2.08
SBOA	2.62	3.11	2.92	3.02
BKA	7.38	6.68	6.03	7.20
RBMO	4.25	4.00	4.42	4.00
DBO	5.75	6.57	6.47	5.88
HHO	9.75	9.75	9.88	9.97
WOA	9.25	9.25	9.07	9.03
GWO	6.88	5.39	6.65	6.12
PSO	5.50	5.04	6.28	6.35

The best mean rank in each function group is in bold.

Two post hoc procedures are reported because they answer different questions, and the honest reading of both requires care about what is being pooled. The Nemenyi test compares all forty-five pairs simultaneously and is correspondingly conservative. At the pooled CD of 1.26 rank units, it places MSCPO ahead of every algorithm except CPO, from which it is separated by 1.23 units; in the single-dimension diagrams of Figure 10 (CD = 2.52), it separates MSCPO from neither CPO nor SBOA (Table 9). A Holm-corrected sequence of comparisons with MSCPO as the control is more powerful and is the appropriate procedure when one algorithm is the object of the study. Applied to the pooled 116 function–dimension blocks, it rejects equality against all nine comparison algorithms, including CPO (z=3.10, adjusted p = 0.002), the largest adjusted p-value over the nine being 0.00193. Table 9 reports both procedures in full for each dimension and for the pooled data: the z statistic of every comparison with MSCPO, its Holm-adjusted *p*-value and whether the Nemenyi test separates the pair.

Applied one dimension at a time, with only 29 blocks each, the same procedure separates MSCPO from seven of the nine at D=10, 7 at D=30, 7 at D=50 and 8 at D=100, but it does not separate MSCPO from CPO at any single dimension (adjusted p = 0.24, 0.09, 0.11 and 0.08 respectively). This should be stated plainly: the significance of the MSCPO-CPO difference rests on pooling the four dimensions, and pooling treats the same function at four dimensions as four blocks, which are not independent of one another. The function-level evidence reported next is what carries the comparison against CPO, and it does not depend on pooling: on 81 of the 116 function–dimension pairs the distributions of the thirty runs differ significantly in MSCPO’s favour and on one in CPO’s at the unadjusted 0.05 level; after a Holm adjustment over the 116 pairs MSCPO still wins 57 and loses none, and under the Benjamini–Hochberg procedure it wins 79 and loses one.

**Table 9:** Friedman omnibus statistics and post hoc comparisons of the nine algorithms with MSCPO as the control on the CEC2017 suite (α=0.05).

Statistic	D = 10	D = 30	D = 50	D = 100	Pooled
Blocks n/algorithms k	29/10	29/10	29/10	29/10	116/10
Friedman $\chi_{F}^{2}$ (df = 9)	215.10	220.01	226.37	225.18	878.54
*p*-value	<0.001	<0.001	<0.001	<0.001	<0.001
Iman–Davenport $F_{ID}$	131.21	150.30	183.03	176.02	610.60
df of $F_{ID}$; *p*-value	(9, 252); <0.001	(9, 252); <0.001	(9, 252); <0.001	(9, 252); <0.001	(9, 1035); <0.001
Nemenyi CD	2.516	2.516	2.516	2.516	1.258
CPO vs. MSCPO	1.17 (0.242) ^†^	1.69 (0.091) ^†^	1.60 (0.109) ^†^	1.73 (0.083) ^†^	**3.10 (0.002)** ^†^
SBOA vs. MSCPO	2.17 (0.060) ^†^	2.08 (0.075) ^†^	1.99 (0.092) ^†^	**2.26 (0.048)** ^†^	**4.25 (<0.001)**
BKA vs. MSCPO	**5.46 (<0.001)**	**6.59 (<0.001)**	**7.37 (<0.001)**	**7.68 (<0.001)**	**13.55 (<0.001)**
RBMO vs. MSCPO	**3.47 (0.002)**	**3.17 (0.005)**	**3.73 (<0.001)**	**4.08 (<0.001)**	**7.22 (<0.001)**
DBO vs. MSCPO	**6.16 (<0.001)**	**6.29 (<0.001)**	**6.12 (<0.001)**	**6.33 (<0.001)**	**12.45 (<0.001)**
HHO vs. MSCPO	**10.58 (<0.001)**	**10.76 (<0.001)**	**10.84 (<0.001)**	**10.97 (<0.001)**	**21.58 (<0.001)**
WOA vs. MSCPO	**9.67 (<0.001)**	**9.76 (<0.001)**	**9.93 (<0.001)**	**9.97 (<0.001)**	**19.67 (<0.001)**
GWO vs. MSCPO	**6.64 (<0.001)**	**6.46 (<0.001)**	**6.07 (<0.001)**	**5.42 (<0.001)**	**12.30 (<0.001)**
PSO vs. MSCPO	**5.64 (<0.001)**	**5.90 (<0.001)**	**5.90 (<0.001)**	**5.98 (<0.001)**	**11.71 (<0.001)**

The first six rows give the omnibus tests; the last nine rows give z (Holm-adjusted two-sided *p*-value) for the comparison of the algorithm in the row with MSCPO as the control, with $z=({\bar{R}}_{j}-{\bar{R}}_{MSCPO})/\sqrt{k(k+1)/(6n)}$, so that a positive z means that MSCPO has the better mean rank. Adjusted *p*-values below 0.05 are in bold; ^†^ marks pairs that the Nemenyi test does not separate (mean-rank difference below the CD).

**Figure 11:**
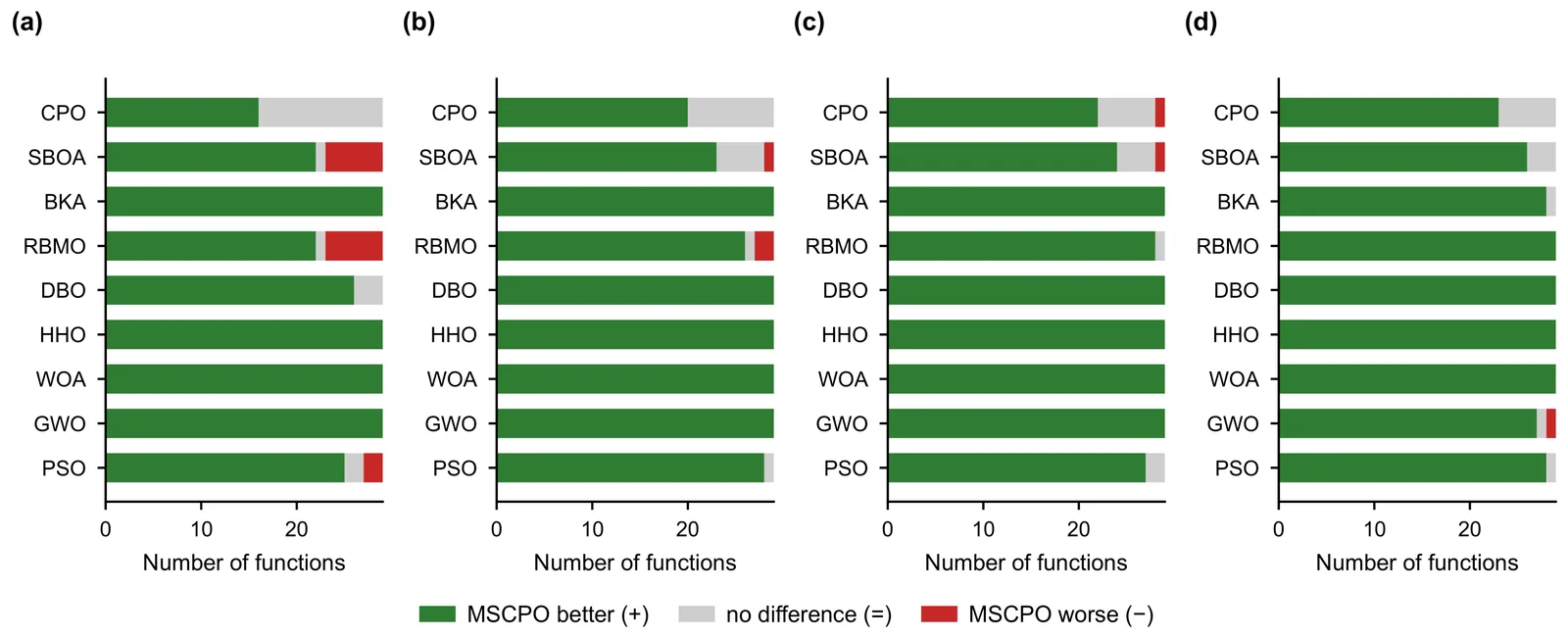
Wilcoxon rank-sum outcomes of MSCPO versus the comparison algorithms (α=0.05): (**a**) D=10; (**b**) D=30; (**c**) D=50; (**d**) D=100.

The Wilcoxon rank-sum tests of Table 10, shown graphically in Figure 11, take the same conclusion down to the level of individual functions. Over the 116 function–dimension pairs, MSCPO is significantly better than CPO on 81, indistinguishable on 34 and significantly worse on one; against HHO and WOA it wins every pair. The single loss against CPO and the 16 losses against SBOA and RBMO are concentrated at low dimension: 12 of the 17 occur at D=10, three at D=30, two at D=50 and zero at D=100. That is the pattern the design predicts. A population of thirty individuals covers a ten-dimensional box far more completely, relative to its volume, than a hundred-dimensional one, so neither a low-discrepancy start nor a fitness-truncated population buys much there; the value of all three strategies grows with the volume of the space they have to cover.

### 4.5. Exploration, Exploitation and Population Diversity

**Figure 12:**
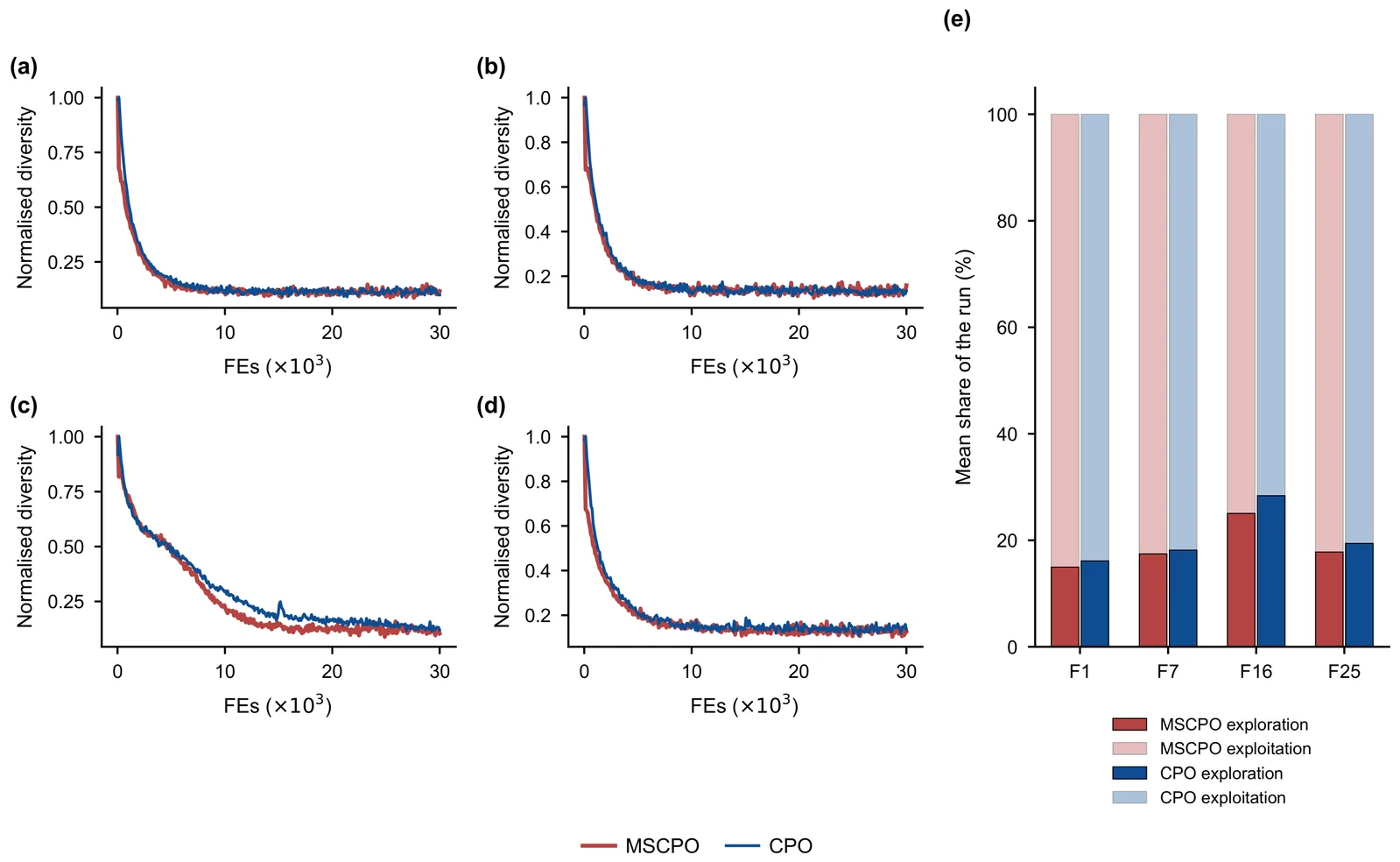
Search behaviour of MSCPO and CPO (D=30): normalised diversity on (**a**) F1, (**b**) F7, (**c**) F16, (**d**) F25; (**e**) exploration–exploitation shares.

Rank tables show which algorithm wins; they do not show why. This section measures the behaviour that the three strategies are supposed to change. Following the standard construction, population diversity at iteration t is the mean absolute deviation of the population from its dimension-wise median, normalised by the range of the search box, as in Equation (26), and the exploration and exploitation percentages are derived from it by Equation (27).

(26) $$\mathrm{Div}(t)=\frac{1}{N_{t}D}\sum_{i=1}^{N_{t}}\sum_{j=1}^{D}\frac{\left|x_{ij}^{t}-{med}_{j}\left(X^{t}\right)\right|}{ub_{j}-lb_{j}}$$

(27) $$XPL\%(t)=100\frac{\mathrm{Div}(t)}{{\mathrm{Div}}_{max}},\;XPT\%(t)=100\frac{\left|\mathrm{Div}(t)-{\mathrm{Div}}_{max}\right|}{{\mathrm{Div}}_{max}}$$

with ${\mathrm{Div}}_{max}={max}_{t}\mathrm{Div}(t)$. Both quantities were recorded for MSCPO and CPO on four functions, one from each CEC2017 group, over 15 independent runs at D=30; Figure 12 shows the result.

The first reading of Figure 12 is a negative one, and it matters. MSCPO does not maintain more population diversity than CPO. Both collapse to roughly a tenth of their peak within the first tenth of the budget and stay there: the mean diversity at the end of the run is 12.6 per cent of the peak for MSCPO and 12.2 per cent for CPO, and over the whole run MSCPO spends 18.8 per cent of the budget in the exploration regime against 20.5 per cent for CPO, so if anything it exploits slightly more. Whatever produces the improvement in Section 4.4, it is not extra exploration.

What does differ is how productive the late search is. Counting every improvement of the incumbent best in the second half of the evaluation budget, MSCPO records 80 on average against 67 for CPO, and its longest stagnation—the largest gap between two consecutive improvements—is 1853 evaluations against 2061. MSCPO therefore keeps finding better solutions in a region of the space that is no larger than the one CPO searches. That is exactly the signature of the elite-guided operators of Section 3.2: drawing the guide from a pool of the fittest individuals rather than from the single incumbent gives the exploitation phase several distinct directions to work in without requiring the population to spread out, and the ablation of Section 4.3, where EGE is the largest single contributor, points to the same mechanism. One panel qualifies the picture: on the hybrid function F16 CPO both keeps more diversity and improves marginally more often, and it is on the hybrid group that the two algorithms are closest at low dimension.

### 4.6. Computational Cost

Mean wall-clock time per run, averaged over all functions and dimensions, is 1.40 s for MSCPO against 1.14 s for CPO, an overhead of 22 per cent, and ranges from 0.60 s for PSO to 1.40 s for MSCPO. The overhead comes from the Sobol generator, which is paid once, and from the per-iteration ordering of at most thirty individuals required by the elite pool and the truncation, which is the O(TlogN) term of Equation (25). Since every algorithm is given the same number of FEs rather than the same wall-clock time, and since in both applications of Section 5 one objective evaluation costs orders of magnitude more than one iteration of bookkeeping, the overhead is irrelevant in the setting the algorithm is meant for.

### 4.7. Behaviour at the Full CEC2017 Evaluation Budget

The protocol of Section 4.1 gives every algorithm 30,000 evaluations at every dimension. The CEC2017 technical report specifies $10^{4}D$, which is ten times that at D=30 and more than thirty times at D=100. Because a short budget rewards fast convergence, and because two of the three strategies of Section 3 are explicitly about how quickly the search concentrates, the comparison has to be repeated at the full budget before any claim about MSCPO can be regarded as settled. Table 11 does that for the four strongest algorithms of Table 7—MSCPO, CPO, SBOA and RBMO—on the complete suite at D=30 and D=100, with 10 independent runs, 300,000 and 1,000,000 evaluations respectively; in practice the latter budget is 999,990 evaluations, the largest multiple of N=30 that does not exceed $10^{6}$.

The result is a clear qualification of Section 4.4, and it should be stated without hedging. Against SBOA and RBMO, the advantage of MSCPO survives the change in budget, although it narrows: in Table 11, its lead in mean rank over SBOA falls from 1.55 to 0.90 at D=30 and from 1.69 to 1.24 at D=100, and its lead over RBMO falls from 2.21 to 1.35 and from 2.69 to 1.45. At D=100 it wins 17 of the 29 functions against SBOA and 15 against RBMO, with Holm-adjusted p-values of 0.0005 and 0.0001. Against its own base algorithm, it does not. At D=30 the mean ranks reverse—CPO 1.66 against MSCPO 2.03, where at the short budget they were 2.31 and 1.31—and the Wilcoxon tests give MSCPO three wins, 20 ties and six losses. At D=100 MSCPO is still nominally ahead (1.76 against 2.03, 5/23/1), but the difference is not significant (adjusted p = 0.42). The honest summary is that the improvement of MSCPO over CPO is a short-budget phenomenon: it is large and significant when each algorithm is given of the order of $10^{3}D$ evaluations and it disappears when each is given $10^{4}D$.

Two things follow. The first is a boundary on the contribution: if the objective is cheap enough that a million evaluations is affordable, the three strategies buy nothing over CPO, and on a ten-times-longer run at D=30 they cost a little. The mechanism is suggested by Section 4.3 and Section 4.5 rather than established: the contribution of APR comes from the smaller population it reaches late in the run, and MSCPO spends slightly less of its budget in the exploration regime than CPO (18.8 against 20.5 per cent), which is an advantage while evaluations are scarce and may become a liability once they are not, because the truncated population has less left to explore with. The second is that the regime the paper cares about is the short one. Each evaluation of the childcare objective of Section 5.1 requires a G2SFCA computation over every zone and site, and each evaluation of the scheduling objective of Section 5.2 requires decoding and scoring a complete roster; a budget of $10^{4}D$ evaluations is not available for either at realistic sizes, and it is not available for the many applied problems that resemble them. The claim this paper can defend is therefore narrower than the CEC2017 rank table alone would suggest, and it is stated that way in the abstract and the conclusions. Such budget dependence is not peculiar to MSCPO: large-scale comparisons show that the ranking of metaheuristics can change with the number of FEs allowed, and recommend reporting rankings separately for each budget [78].

### 4.8. Robustness Analysis

Robustness is understood here in three senses, all measured on the data of Section 4.4: stability from run to run, protection against bad outcomes and consistency across problems. For every function–dimension pair, the ten algorithms are ranked by the standard deviation and by the worst of their thirty final errors, and Table 12 reports the mean of these ranks over the 116 pairs, the number of pairs on which each algorithm attains the smallest value, and two indicators of consistency across problems computed from the per-pair ranks of the mean error: their standard deviation and their worst value. No enhanced CPO variant is included. The variants of Table 1 were published under iteration budgets, each with its own operators and constants, and several are only partly described in the sources we could read, so converting them to the common FE budget would test our reading of them rather than the published algorithms; the nine comparison algorithms raise the same issue to a smaller degree, since they are established methods with widely used reference implementations. Their design and evaluation protocols are compared in Section 6.1, and a common, fully budgeted comparison is listed as further research in Section 6.4.

MSCPO is the most robust of the ten algorithms in all three senses. Its thirty runs are the least dispersed: the mean rank of its standard deviation is 1.76, against 2.19 for CPO and 3.17 for SBOA, and it has the smallest standard deviation on 63 of the 116 pairs, CPO on 38 and no other algorithm on more than nine. Its worst error is the smallest of the ten on 80 pairs, with a mean rank of 1.38 against 2.25 for CPO; it is below CPO’s on 89 pairs and below that of every other competitor on at least 106. Across problems, it never ranks below fourth on any pair and is in the top three on 115 of the 116, whereas CPO falls as low as eighth and is in the top three on 101; the standard deviation of its per-pair rank, 0.53, is the smallest among the algorithms in the upper half of the field, HHO and WOA having smaller values only because they are almost always last. The differences are significant on both dispersion measures (Friedman tests in the note to Table 12).

Two further forms of robustness were examined above and complete the picture. MSCPO is only moderately sensitive to its own parameters: the differences between settings are significant (Table 3 and Table 4), but its overall mean rank varies by at most 0.95 units on a four-point scale (at most 1.17 at D=100), against 8.58 units between the best and the worst algorithm on the ten-point scale of Section 4.4. Its advantage over SBOA and RBMO, the strongest competitors after CPO, also survives, although it narrows, a ten-fold (D=30) and a thirty-three-fold (D=100) increase in the evaluation budget, whereas its advantage over CPO does not (Section 4.7), so its robustness with respect to the budget is the one respect in which it is not better than its base algorithm. The applications of Section 5 repeat the benchmark pattern only in part: MSCPO has the smallest standard deviation on three of the four childcare instances (Section 5.1.4), but on the scheduling instances (Section 5.2.4) its dispersion is close to CPO’s and, on the largest instance, larger than that of SBOA, RBMO and PSO.

## 5. Application to Work–Family Resource Allocation

Benchmark suites establish that an optimiser searches well; they do not establish that it is useful. The applications reported in the bio-inspired optimisation literature are overwhelmingly drawn from a small set of mechanical design problems—the welded beam, the pressure vessel, the three-bar truss—that have been solved thousands of times and whose optima are known to four significant figures. This section takes MSCPO somewhere else: to the allocation of the resources that determine how much of women’s labour reaches the market.

**Figure 13:**
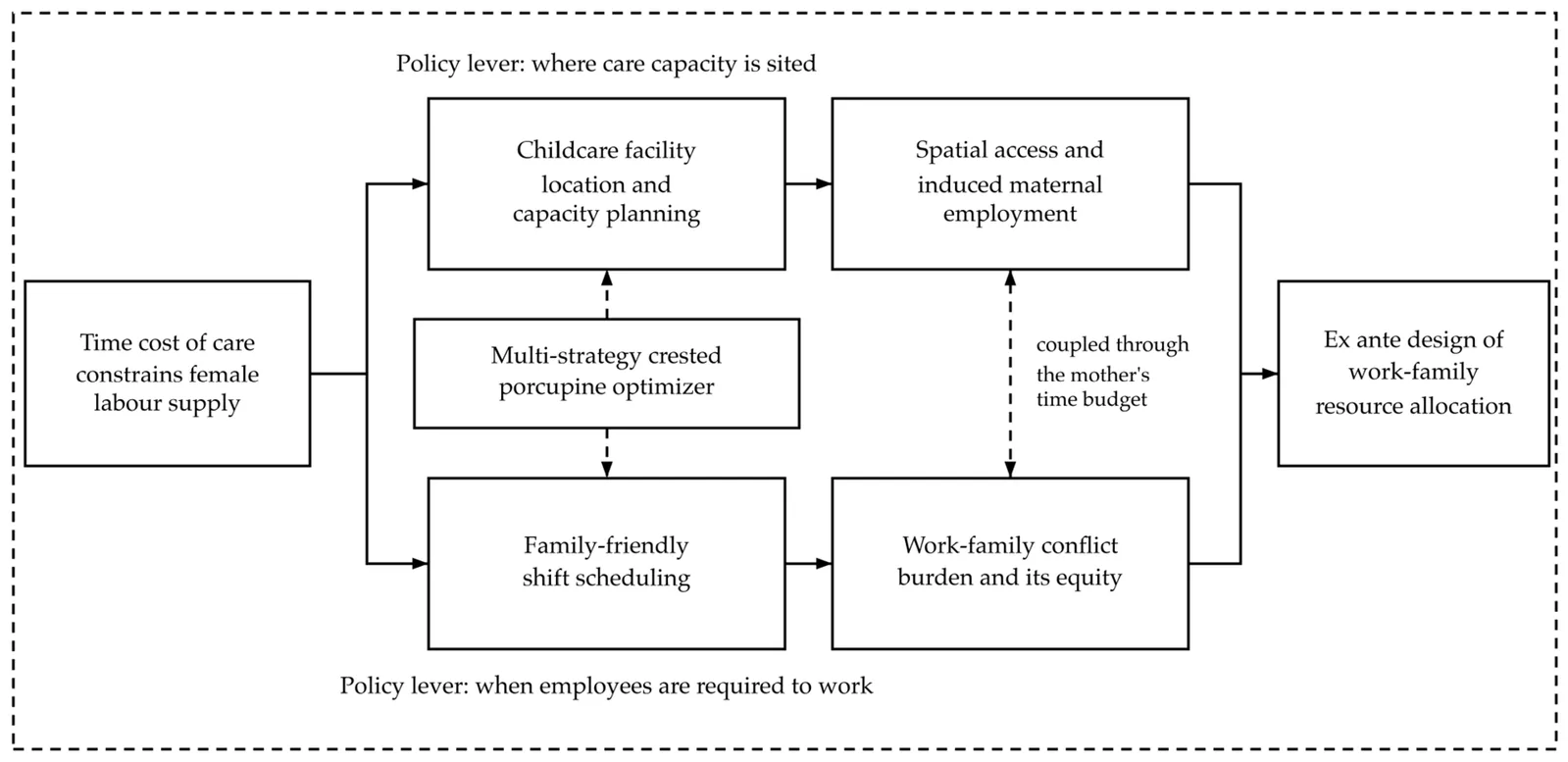
Conceptual framework of the two work–family resource-allocation problems addressed by MSCPO.

The logic of the two problems is set out in Figure 13. Mothers of young children face a time cost of care that has to be paid before any market work is possible. Two policy levers act on that cost. The first is spatial: where public childcare capacity is placed, and how much of it, determines how far a mother must travel and whether a place is available at all; it works on the extensive margin, that is, on whether she participates. The second is temporal: how a firm distributes shifts across its employees determines how much of the resulting job conflicts with care obligations; it works on the intensive margin and on retention. Both levers are exercised under a hard constraint—a public budget in the first case, a staffing requirement in the second—and both produce objective functions that are non-convex, non-differentiable and discontinuous in their decision variables. Neither is amenable to the reduced-form methods with which the effects of these policies are usually estimated; both are natural targets for a population-based optimiser.

In both applications, the ten algorithms are run with exactly the parameter settings of Table 2 and exactly the protocol of Section 4.1, so that the comparison carries over from the benchmark study without modification. All problem instances, their generators and the raw results are available from the corresponding author on request.

### 5.1. Childcare Facility Location and Capacity Optimisation

#### 5.1.1. Economic Background

The time cost of caring for a child under three is the most immediate constraint on maternal labour supply, and formal childcare is the instrument that relaxes it most directly. The price channel is genuine: married US mothers who move to a state with more expensive childcare have about 18% lower odds of employment than comparable movers to a cheaper state [84], and in China doubling childcare-centre tuition reduces a mother’s market work by 1.3 hours per week [85]. Availability acts on a different margin of the same time budget: in the same study, access to a local childcare centre cuts a mother’s own childcare time by 13.3 hours per week [85]. The two figures are not directly comparable: one concerns market work and the other care time, and in that study access did not measurably change the mother’s time outside childcare [85]. Together they locate the constraint, the block of care hours that a nursery place can take over. Where provision is rationed rather than priced, the binding constraint is a queue and a travel distance, and subsidised families travel further to childcare than the distribution of capacity requires [86]. Chinese evidence points the same way: public child supervision raises maternal employment through the supply side, substituting for maternal care [87].

Quasi-experimental work has put magnitudes on this margin. A one-percentage-point increase in childcare slots for under-threes raises mothers’ labour force participation by 0.2 percentage points in Germany [47]; the East Asian counterpart is 0.27 points per percentage point of capacity for ages 0–5 in Japan [48]. A larger effect, measured on a different scale, comes from a setting with very low baseline provision: attending public childcare, rolled out by neighbourhood-level randomisation in poor urban Nicaragua, raised mothers’ work by 12 percentage points [49]. Two findings discipline a naive dose–response reading. The hours dimension is sharply non-linear: in England free part-time childcare barely moved participation, whereas the full school day produced significant participation and employment gains [51]. And the response depends on who is treated: a Ugandan trial found a childcare subsidy raised single mothers’ labour supply and earnings but not partnered mothers’ [88].

Heterogeneity of this kind is not a nuisance; it is the reason siting matters. Harmonised estimates across seven countries find significant effects in Austria, the Czech Republic, Hungary and the Slovak Republic but none in France, Greece and Italy, with effectiveness tracking the gap between the participation rate of mothers of older children and under-three coverage [50]. Six decades of Austrian parental-leave and childcare expansion, including nursery expansions from a low baseline, produced virtually no gender convergence in earnings, which the authors attribute to formal care crowding out informal care and to persistent norms of maternal care [89], and cross-country child penalties vary sharply with development and labour-market structure [40]. If the marginal return to a nursery place is largest where baseline provision is lowest relative to mothers’ attachment to the labour market, then where a fixed budget is placed, not merely how many slots it buys, determines the aggregate response. Brazilian evidence makes this quantitative: siting rules targeting neighbourhood income or excess demand achieve only about 60% of the maternal-employment gains attainable under the optimal allocation of new centres [60]. The residual is an optimisation problem, the one set out in Figure 13 and addressed here.

Table 13 shows that these estimates are neither isolated nor confined to one period. It lists the principal experimental, quasi-experimental and observational estimates quoted in this subsection, with the setting, the period of the policy variation or data, the design and the estimate as reported. The evidence spans three decades and four continents, and the designs range from randomised trials to regression discontinuities and event studies; because the units differ, the magnitudes cannot be pooled, but the signs agree. None of the studies finds that more available or cheaper childcare reduces maternal labour supply, and the null results are concentrated in settings for which the original studies offer an explanation: coverage that is already high (France), low participation even among mothers of older children (Greece, Italy), an entitlement too short to fit a working day (the English part-time offer), freed time absorbed by another household member (partnered mothers in Uganda), or formal care displacing informal care under strong norms of maternal care (Austria). The recent reviews provide broader context without supplying a common response coefficient. A review of child penalties discusses childcare policy among the mechanisms behind these penalties [52]. A review of 22 causal studies in lower- and middle-income countries finds some positive maternal labour-market effect in all but one study, with differences in programme hours, eligibility and affected subpopulations [53]. Together, these reviews support treating the slope of the present childcare model as uncertain rather than universally transferable. The response is therefore positive where childcare is the binding constraint and heterogeneous in size in a predictable way: larger where under-three provision is low relative to mothers’ attachment to the labour market, and small or absent where coverage is already high or where even mothers of older children rarely work. The model of Section 5.1.2 captures this heterogeneity only in part: the logit response of Equation (33) has diminishing returns as coverage rises, but its marginal effect at baseline, $\beta_{A}e_{i}^{0}(1-e_{i}^{0})$, is largest at a participation rate of one half rather than where participation is lowest (Section 5.1.4), which is one reason why the calibrated slope is varied in Section 5.1.4 rather than treated as known.

The Chinese setting sharpens it. Inclusive 0–3 nursery provision is decentralised and uneven across the 31 mainland provinces [46], so no single national parameter describes the constraint. The sequence from one child to two and then three [90] raised the expected care burden without a commensurate expansion of formal supply: after the universal two-child policy, women’s labour force participation fell by 4.7 percentage points while elderly participation in informal work rose by 2.6 points [45]. The motherhood penalty is well documented, although its size and trend depend on the margin and the period: about a third of working women leave the labour market in the year of birth, and the employment penalty grew between 2000 and 2015 [42]; the overall labour-market effect is smaller and recovers faster than in other countries but has increased over the decades, with grandmothers cutting their own labour supply to provide care [44]; and the motherhood wage penalty in urban China narrowed between 2002 and 2018 [43]. The penalty operates through multi-generational rather than nuclear household structure [91], and is reinforced by discrimination based on perceived fertility intentions [92]. Grandparental care is an imperfect substitute: it lifts mothers’ labour supply at the extensive but not the intensive margin [93], contracts when older women’s own employment is extended [94], and does not reach households without a nearby grandparent. Chinese households also do not treat cash allowances and in-kind services as interchangeable [95], so service siting, not a transfer, is the decision variable here.

How accessibility is measured determines what is optimised. A count of centres within an administrative boundary ignores competition for places and distance decay, and mobility data show that nominal proximity does not equalise actual utilisation [96]; behaviour-based supply–demand matching within the fifteen-minute community life circle is the appropriate Chinese-city framing [97]. Floating-catchment statistics, which weight supply by distance-decayed demand and demand by distance-decayed supply, serve as objective and as ex post metric for location-allocation plans in Chinese cities [98], and such indicators can replace a binary service radius inside a covering objective [61]. Equity belongs in that objective rather than in a later audit: pure coverage maximisation is inequitable [65] and leaves assignment-side inequity unaddressed [66], so accessibility and equity are best treated jointly and stated explicitly in the formulation, whether as an envy-based objective alongside cost and accessibility [99], as one of several equity measures compared within a robust p-median model [100], or through the fairness formulations surveyed in [101]. For Beijing education facilities, precisely this pairing decides whether an allocation is an improvement [102].

The planning question is therefore joint: which sites to open, and how large to make each, under a budget. Joint location and sizing separates capacity planning from pure siting [63]; the resulting capacitated covering problem with graded coverage is a recognised metaheuristic target [62,64], whose closest applied analogue is hierarchical location-allocation for public family-oriented care [103]. Related capacitated location models admit exact and heuristic solvers [67], whose scalability on CFLC remains untested. The present objective is neither convex nor differentiable. The CFLC problem is accordingly formulated below as a capacitated location-capacity model over the candidate sites and residential zones of Section 5.1.3, and solved by MSCPO.

#### 5.1.2. Model Formulation

A city is partitioned into I residential zones and offers J candidate sites for public nursery centres serving children aged 0–3. Zone i contains $m_{i}$ children in that age band; since the model counts one such child per mother, $m_{i}$ is also the number of mothers of a child aged 0–3 in the zone, of whom a fraction $e_{i}^{0}$ is currently employed. A mother who enters employment in zone i earns $w_{i}$ per year, and the travel time from zone i to site j is $d_{ij}$. Opening site j costs $c_{j}^{f}$ per year, and each subsidised place costs $c^{v}$ per year; site j can hold at most $q_{j}^{max}$ places, and a centre is not viable below $q^{min}$ places. The planner has an annual budget B.

The decision vector is $z\in [0,1{]}^{J}$, one continuous key per candidate site, decoded by Equations (28) and (29).

(28) $$y_{j}=\left\{\begin{array}{ll} 1, & z_{j}\geq 0.5\\ 0, & \mathrm{otherwise} \end{array}\right.$$

(29) $$q_{j}=y_{j}\left[q^{min}+\frac{z_{j}-0.5}{0.5}\left(q_{j}^{max}-q^{min}\right)\right]$$

so that z simultaneously selects the sites and sizes them; capacities are rounded to the nearest tens place. The search dimension is therefore D=J.

Spatial accessibility is measured with the G2SFCA statistic. The distance-decay weight of Equation (30) is zero beyond a catchment radius $d_{0}$ and falls smoothly to zero at that radius.

(30) $$G\left(d\right)=\left\{\begin{array}{ll} \frac{\exp\left(-\frac{1}{2}{\left(d/d_{0}\right)}^{2}\right)-\exp\left(-\frac{1}{2}\right)}{1-\exp\left(-\frac{1}{2}\right)}, & d\leq d_{0}\\ 0, & d>d_{0} \end{array}\right.$$

In the first step, each site’s provision-to-demand ratio is computed over the population it can reach, as in Equation (31); in the second, each zone sums the ratios of the sites it can reach, as in Equation (32).

(31) $$R_{j}(z)=\frac{q_{j}+q_{j}^{ex}}{\sum_{i=1}^{I}m_{i}G(d_{ij})}$$

(32) $$A_{i}(z)=\sum_{j=1}^{J}R_{j}(z)G(d_{ij})$$

where $q_{j}^{ex}$ is the capacity already in place at site j. Existing places count towards accessibility whether or not the site is selected. Selecting a site that already holds places ($y_{j}=1$) means expanding it: the $q_{j}$ new places, bounded by $q_{j}^{max}$, are added to the $q_{j}^{ex}$ already there, and the full fixed cost $c_{j}^{f}$ of Equation (35) is incurred. The statistic has a direct interpretation: it is a local childcare coverage rate, places per child, weighted by travel-time accessibility, and its population-weighted mean over the city equals total places divided by total children, so the measure conserves supply.

Maternal employment responds to local coverage through the logit model of Equation (33), whose intercept is calibrated zone by zone so that the model reproduces the observed baseline $e_{i}^{0}$ at the baseline accessibility $a_{i}^{0}$.

(33) $$e_{i}(z)=min\left\{\sigma \left(\sigma^{-1}\left(e_{i}^{0}\right)+\beta_{A}\left[A_{i}(z)-a_{i}^{0}\right]\right),\bar{e}\right\},\;\sigma (u)=\frac{1}{1+e^{-u}}$$

The slope $\beta_{A}$ is the only behavioural parameter of the model. It is set so that the marginal effect of local coverage on maternal employment, $\partial e_{i}/\partial A_{i}=\beta_{A}e_{i}^{0}(1-e_{i}^{0})$, equals 0.35 at the mean baseline participation rate: a ten-percentage-point increase in local coverage raises maternal employment by three and a half percentage points. That figure lies above both estimates available in the same unit, 0.20 for West Germany [47] and 0.27 for Japan [48]; the larger Nicaraguan effect [49] is an effect of attending a centre, not a slope per point of coverage, and cannot serve as an upper bound, so the value is an optimistic rather than a central choice. We found no estimate in this unit for China, so the slope is transferred from West German (2007–2014) and Japanese (2015–2017) data (Table 13), whose stability over time has not been established. For both reasons, Section 5.1.4 reports how the optimised programme changes when the slope is set to each of the two lower values instead. The cap $\bar{e}$ prevents the model from extrapolating beyond the highest participation rates observed in the data. Because σ is concave above its inflexion point, the response exhibits diminishing returns in coverage, so concentrating all places in one district is penalised automatically.

The gross social value of the programme is the market value of the additional maternal labour it induces, Equation (34); its cost is the sum of fixed and per-place costs, Equation (35).

(34) $$V(z)=\eta \sum_{i=1}^{I}m_{i}max\left\{e_{i}(z)-e_{i}^{0},0\right\}w_{i}$$

(35) $$C(z)=\sum_{j=1}^{J}\left(y_{j}c_{j}^{f}+c^{v}q_{j}\right)$$

in which η is an earnings realisation factor that accounts for the induced entrants who work less than a full year.

A planner who maximised V−C alone would place capacity where earnings are highest, which in a monocentric city means the centre. Spatial equity therefore enters the objective through the population-weighted coefficient of variation (CV) of accessibility, Equation (36), and the budget through a relative penalty. The problem solved by the optimiser is the minimisation of Equation (37).

(36) $$CV(z)=\frac{1}{\bar{A}}\sqrt{\frac{\sum_{i}m_{i}{\left[A_{i}(z)-\bar{A}\right]}^{2}}{\sum_{i}m_{i}}},\bar{A}=\frac{\sum_{i}m_{i}A_{i}(z)}{\sum_{i}m_{i}}$$

(37) $${min}_{z\in [0,1{]}^{J}}F_{CFLC}(z)=-\frac{V(z)-C(z)}{\kappa }+\mu CV(z)+Pmax\left\{0,\frac{C(z)-B}{B}\right\}$$

where κ is a monetary scaling constant, μ is the weight on spatial inequality, and P is the budget-violation penalty. The objective is discontinuous at every $z_{j}=0.5$, because a site opens there and the fixed cost $c_{j}^{f}$ jumps; it is piecewise smooth but non-convex between those points; and the number of sign patterns of y is $2^{J}$. No gradient is available, and enumeration is infeasible beyond the smallest instance.

Table 14 collects the elements of the model in one place. The decision variables are the J site keys; the objective is Equation (37); the capacity, minimum-size and integrality constraints hold by construction of the decoder, so every decoded solution is feasible except possibly with respect to the budget, which is a soft constraint enforced by the relative penalty P; and equity is measured by the CV of Equation (36), which is zero when every zone has the same accessibility and grows with the dispersion of accessibility around its population-weighted mean.

#### 5.1.3. Instances

Four instances of increasing size are generated by the calibrated instance generator, which is available from the corresponding author on request. The city is monocentric: the density of children aged 0–3 decays exponentially with distance from the centre, baseline maternal employment falls with distance, potential earnings fall with distance, and land prices—and therefore the fixed cost of a centre—rise towards the centre. Existing provision is placed at a quarter of the candidate sites, drawn at random, which reproduces the patchy sub-national pattern documented in Section 5.1.1. Travel times are Euclidean distances inflated by a network detour factor of 1.30 and converted at a door-to-door speed of 22 km/h. The annual budget is set at 2600 Chinese yuan (CNY) per child aged 0–3, which, at the calibrated unit costs, buys places for roughly one seventh of the age group. Quantitatively, the city is a disc of radius $R_{c}=18$ km. Zone centroids are placed at radius $R_{c}u^{0.425}$ and candidate sites at $R_{c}u^{0.375}$, with u uniform on [0,1] and uniform angles. The number of mothers of a child aged 0–3 in a zone at distance r from the centre is proportional to $exp(-r/(0.55R_{c}))$, scaled to an average of 260 per zone, multiplied by log-normal noise with σ=0.28 and bounded below by 30. Baseline maternal employment is $0.72-0.20r/R_{c}$ plus normal noise with standard deviation 0.035, truncated to [0.30,0.88], and potential annual earnings are $108,000exp(-0.30r/R_{c})$ CNY with log-normal noise (σ=0.22). The annualised fixed cost of a centre is 750,000 CNY multiplied by $1+0.55exp(-r/(0.5R_{c}))$ and by log-normal noise (σ=0.12); the ceiling on the new places at each candidate site is drawn uniformly from 90, 120, 150, 180 and 210; and each existing centre holds between 40 and 109 places, to which new places are added if its site is selected. Table 15 summarises the four instances.

**Table 15:** The four CFLC instances.

Instance	I	J	D	Children Aged 0–3	Existing Places	Budget (Million CNY)	Mean Baseline Coverage
CFLC-1	40	20	20	10,312	367	26.8	3.6 per 100
CFLC-2	80	40	40	20,676	722	53.8	3.5 per 100
CFLC-3	150	60	60	41,902	1159	109.0	2.8 per 100
CFLC-4	250	100	100	66,418	1849	172.7	2.8 per 100

I is the number of residential zones, J the number of candidate sites, and D=J the search dimension. Calibration constants, identical across the four instances: catchment radius $d_{0}=30$ min; logit slope $\beta_{A}=1.45$; employment cap $\bar{e}=0.92$; annual public cost per place $c^{v}=16,000$ CNY; annualised fixed cost of a centre $c^{f}=750,000$ CNY scaled by local land price; minimum viable centre $q^{min}=30$ places; earnings realisation factor η=0.85; equity weight μ=4.0; objective scale $\kappa =10^{7}$ CNY; budget-violation penalty P=50. Travel times use a network detour factor of 1.30 and a door-to-door speed of 22 km/h.

#### 5.1.4. Results

**Figure 14:**
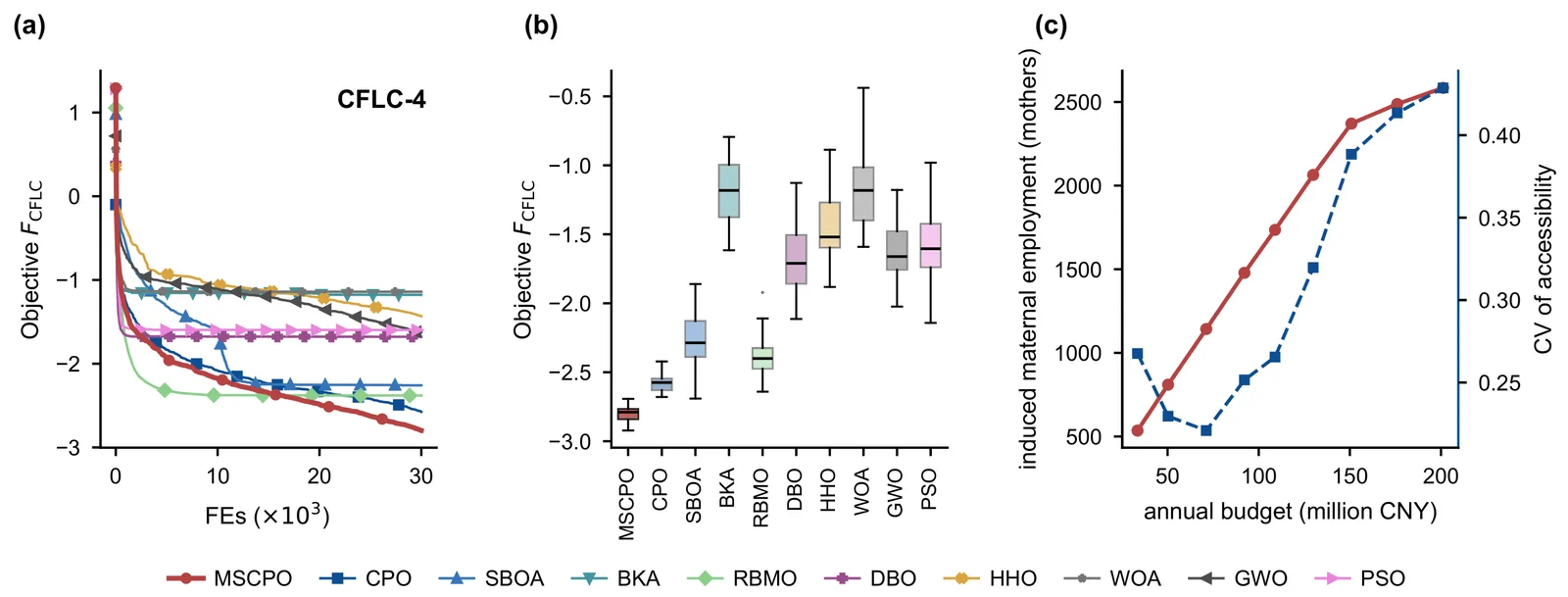
Performance on the CFLC problem: (**a**) convergence curves (CFLC-4); (**b**) objective distribution (CFLC-4); (**c**) optimised programme against the budget (CFLC-3). In (**c**), solid red circles show induced maternal employment (left axis) and dashed blue squares show the coefficient of variation (CV) of accessibility (right axis).

Table 16 reports the objective values, and Figure 14 reports the convergence curves and their dispersion. MSCPO attains the best mean objective on three of the four instances and the best mean rank overall (1.25 against 1.75 for the best competitor), and it is ahead of all nine comparison algorithms. The Friedman test over the four instances rejects equivalence ($\chi_{F}^{2}(9)=35.07$, p < 0.001), and the Wilcoxon rank-sum tests give MSCPO two wins, two ties and zero losses against CPO over the four instances. The ordering of the field is close to the ordering on CEC2017, which is the first piece of evidence that benchmark behaviour transfers to this class of problems; the exception is that the gap between MSCPO and the weaker algorithms is much larger here than on the benchmark, because the objective of Equation (37) is discontinuous at every site-opening threshold and an algorithm that cannot maintain a diverse population across those discontinuities never finds the right subset of sites.

One label needs qualifying before these numbers are read as welfare statements. The ratio reported below and in the tables that follow is the gross annual earnings of the induced entrants divided by the annual public subsidy. It is an output-to-subsidy ratio, not a social benefit–cost ratio: the numerator ignores the value of the parental time that market work displaces and any crowding out of informal or private care, and the denominator ignores the marginal cost of public funds, the fees parents pay and the tax revenue the additional earnings generate. Several of these push in opposite directions—the deadweight loss of taxation raises the true cost, the recovered tax revenue lowers it—and a proper welfare evaluation would need all of them. What the ratio does measure, consistently across allocations, is how much market output a given public outlay buys, which is the quantity the optimiser is comparing across sites. It is used here as such and the term ‘benefit–cost ratio’ is avoided.

**Figure 15:**
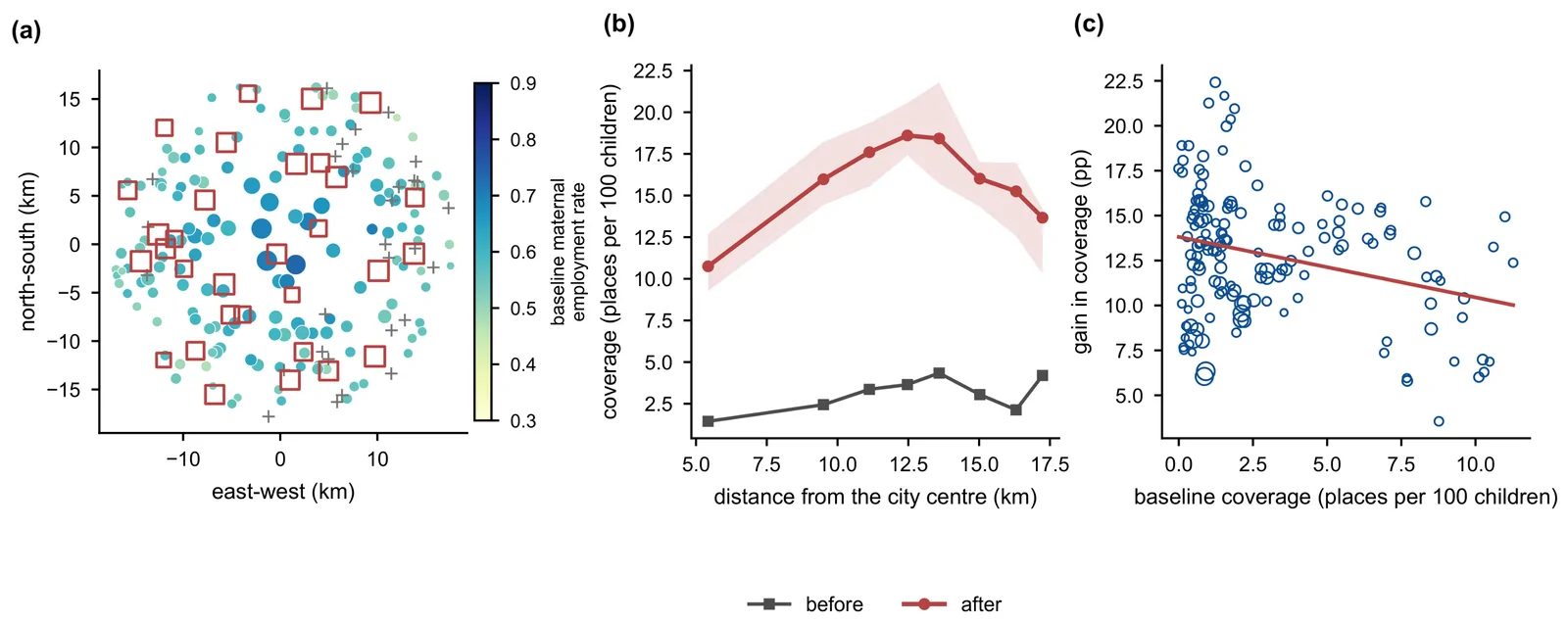
Optimised childcare programme on CFLC-3: (**a**) zones and opened centres; (**b**) coverage profile before and after; (**c**) gain against baseline coverage. In (**a**), circle size increases with child population and colour denotes baseline maternal employment; red outlined squares mark opened centres, with size increasing with assigned capacity. In (**c**), the red line is the least-squares regression of coverage gain on baseline coverage; circle size increases with child population.

The difference between the two optimisers is set out in Table 17, and it is small in absolute terms. On the largest instance, the MSCPO programme opens 49 centres with 8160 places for 172.5 million CNY a year and induces 2744 additional employed mothers; the CPO programme opens the same number of centres, spends 1.5 million CNY less and induces 2715. The marginal 1.5 million CNY that MSCPO spends therefore buys 29 jobs at about 52,884 CNY each, below the average annual earnings of an induced entrant; but it would be an overstatement to present this as a large economic difference. Where the two allocations differ materially is in the spatial distribution of access: the CV of accessibility is 0.232 for MSCPO against 0.250 for CPO in this instance. The direction is not uniform across the four instances—the two allocations are identical on CFLC-1 and CFLC-2, and on CFLC-3 the CPO solution is marginally the more equal of the two—so the honest summary is that a rank-table improvement of the size reported in Section 4 changes the shape of the allocation more than its size, and that the two optimisers are close on this problem. The optimised programme itself is mapped in Figure 15. The map sizes each zone by its number of mothers of a child aged 0–3, marks the unused candidate sites with crosses and draws the opened centres as squares scaled by capacity. The comparison that matters for a planner is with the rules a planner would otherwise use.

Everything in this subsection is conditional on the one behavioural parameter of the model, and it is worth showing how much Table 18 re-solves CFLC-4 with the logit slope recalibrated so that the marginal effect of coverage on maternal employment equals 0.20, 0.27 and 0.35 percentage points per point of coverage, the first two being the German and Japanese estimates quoted in Section 5.1.1 and the third the value used above. The optimised programme is highly sensitive to the choice. At 0.35 it opens 50 centres, spends 99 per cent of the budget and induces 2735 mothers into employment at an output-to-subsidy ratio of 1.23; at 0.27 it opens 12 and induces 597 at a ratio of 1.00; at 0.20 it opens only seven, spends 13 per cent of the budget and induces 207 at a ratio of 0.71. The model is behaving correctly—a planner who believes the low estimate should not build the programme, and the optimiser says so by declining to spend the budget—but the implication for the reader is that the magnitudes reported above are statements about a calibration, not measurements. What survives the recalibration is the shape of the solution rather than its size: the negative relation between the gain in coverage and baseline provision holds at every slope, and the sites opened at 0.27 are a subset of those opened at 0.35. What does not survive is the case for spending the whole budget. None of this affects the comparison between algorithms, which face the same objective whatever its calibration.

That observation raises the question a policy reader will ask next, and it is not a question about optimisers: is optimising the siting worth doing at all, against the rules a planning department would actually use? Table 19 answers it by constructing four such rules—open the sites with the largest catchment population first, the largest unmet-need gap first, the cheapest first, the highest reachable earnings first—and a fifth that spreads the budget thinly over as many small centres as it will pay for, evaluating each with exactly the objective of Equation (37), and comparing with the best solution MSCPO found. The answer has two halves. On induced employment, the sensible rules do almost as well as the optimum: on the largest instance they capture between 93 and 100 per cent of the attainable gain, and on one instance the cheapest-site rule slightly exceeds it. Aggregate maternal employment is, in this calibration, robust to how a given budget is spread, provided it is not spread too thinly: the uniform rule captures only 62 per cent, because the fixed cost of a centre is wasted when each one is too small to matter.

The other half is where the optimisation earns its keep. The CV of accessibility under the four sensible rules ranges from 0.358 to 0.632 on the largest instance, against 0.232 under the optimum: the cheapest-site rule matches the optimum’s employment while leaving the dispersion of access 2.7 times as large. The optimised programme therefore buys a large equity gain at a negligible cost in employment. That is exactly what the objective of Equation (37) was asked to do, since it carries the inequality term explicitly, so the finding is not a surprise; what is informative is the price. A planner who cares only about the headline employment number can use a simple rule and lose little; a planner who also cares about where access ends up cannot, and the cost of caring turns out to be close to zero. This is the practical case for putting an optimiser between the budget and the map, and it is a case that only appears once equity is inside the objective rather than in a post hoc audit.

Table 19 shows three further regularities. First, the rules and the optimum spend the budget differently. On the two largest instances, the four sensible rules open more centres than the optimum (33 against 31 on CFLC-3, and 54 or 55 against 49 on CFLC-4) yet provide the same number of places or fewer, because every additional centre carries a fixed cost; the optimum buys capacity rather than addresses. The uniform rule is the extreme case: it opens every candidate site, or all but one on CFLC-2, at the minimum size, which is why it captures only 47 to 71 per cent of the attainable employment and has the lowest output-to-subsidy ratio on every instance. Second, no rule is uniformly best on employment: the largest-catchment rule leads the rules on CFLC-1 and the cheapest-site rule on the other three, and the share of the optimum captured by the four sensible rules ranges from 92.6 to 100.9 per cent. Third, the rules differ far more on equity than on employment, in a pattern that matches the optimised solutions. The cheapest-site rule, which buys the most places of any rule on every instance and the most employment of the four sensible rules on three of them, produces the most unequal access of all five rules on every instance (CV between 0.632 and 1.621), because in these instances the cheapest sites lie on the periphery, where land costs less, so capacity accumulates where land is cheap rather than where provision is lacking. The provision-gap rule, which, as the next paragraph shows, is closest in spirit to what the optimiser does, gives the most equal access of the four sensible rules on the two largest instances (0.383 and 0.358). Even the most equal rule on each instance, however, leaves the CV 39 to 55 per cent above that of the optimum, so the equity gain of optimisation is not reproduced by any simple ordering of sites.

Two regularities emerge from the optimised solutions, and neither is imposed by the model. The first is that the optimiser targets under-provision rather than low employment. Regressing the gain in local coverage on the zone’s baseline coverage, as plotted in Figure 15c, gives a negative slope on all four instances, significant at the 5 per cent level on three of them and reaching −0.54 percentage points of gain per point of baseline coverage on the largest (p < 0.001): capacity flows to the zones that have least. The corresponding regression on baseline maternal employment is not significant in any instance (p between 0.13 and 0.53) and changes sign across them, so the optimiser does not, in this calibration, favour zones where fewer mothers work. That is worth stating precisely because a naive reading of the heterogeneity results of Section 5.1.1 would direct places to the zones where fewest mothers work, whereas those results point to provision that is low relative to mothers’ attachment to the labour market. The reason is visible in Equation (33): the marginal response to coverage is $\beta_{A}e_{i}^{0}(1-e_{i}^{0})$, which is largest at a participation rate of one half and falls away on both sides, so a zone with very low baseline employment is not automatically the most responsive one. What the optimiser does respond to is a low starting level of provision: accessibility rises linearly with capacity, the logit response flattens as participation rises above one half, and the equity term of Equation (37) rewards raising the lowest accessibilities. A planner reading this result should target provision gaps, not employment gaps.

The second regularity concerns scale. Figure 14c traces the optimised programme on CFLC-3 as the annual budget is raised from 34 to 201 million CNY (the best of 10 independent MSCPO runs at each of nine budget levels). Induced employment rises throughout, from 536 to 2584 mothers, with clearly diminishing returns: the first 92 million CNY actually spent buys 1479 of those jobs and the remaining 72 million buys 1106 more. Two features of the top of the range should be read carefully. First, the optimiser stops spending: at the two largest budgets it uses only 90 and 82 per cent of the money it is given, because the marginal place no longer pays for itself, and the horizontal axis of the figure is therefore the budget offered rather than the budget used. Second, this is not a capacity ceiling: even at the largest budget the programme occupies only about four fifths of the capacity the candidate sites could hold.

The gross output-to-subsidy ratio stays above one throughout but drifts from 1.28 to 1.24, and the inequality of accessibility is U-shaped: the CV falls to a minimum of 0.221 at a budget of 71 million CNY and then rises to 0.429, because beyond the minimum the optimiser has exhausted the under-provided periphery and begins adding capacity where provision is already good. A planner who maximises output per yuan and one who maximises employment subject to an equity ceiling would therefore choose different budgets, and the curve locates the disagreement.

### 5.2. Family-Friendly Shift Scheduling

#### 5.2.1. Economic Background

The second application moves from the spatial to the temporal margin of the care constraint. Where the childcare problem asks where capacity should sit, shift scheduling asks when people are required to be present at work, and the evidence is clear that this second question is not reducible to the first, nor to the total number of hours contracted. Work–family conflict is generated by the timing, the variability and above all the predictability of hours. Dimension-based meta-analytic evidence separates time-based from strain- and behaviour-based conflict and grounds the time-based dimension explicitly in resource-allocation theory [58], while episode-level and diary studies show that conflict is triggered by particular schedule events rather than being a stable individual trait [104,105]. A roster is therefore not a neutral administrative artefact: it is the instrument through which an employer distributes work–family conflict across a workforce.

The consequences are documented on both sides of the employment relationship. In survey data on hourly service-sector workers in the United States, routine work-schedule instability is associated with psychological distress, poor sleep quality and unhappiness more strongly than low wages are, and a much larger share of that association is mediated by work–life conflict than by household economic insecurity [54]. Panel evidence on 1827 hourly retail and food-service employees shows schedule instability robustly predicting job turnover among the roughly one third of workers with relatively unstable schedules, with slightly under half of the relationship mediated by job satisfaction and about a further quarter by work–family conflict [55]. Field evidence from the same sector indicates that flexibility organised around fluctuating demand is asymmetric by default, serving the employer unless worker preferences are represented explicitly in the allocation rule [106]. These single-sector findings agree with syntheses of a much wider literature. In a meta-analysis of 332 studies from 58 countries, working hours correlate with work-to-family conflict (corrected ρ=0.27, based on 136 studies from 51 countries) [57]; a meta-analysis of dimension-based work–family conflict [58] supports distinguishing time-, strain- and behaviour-based conflict. This distinction motivates reporting separate burden components in Equation (41), but neither synthesis supplies the cardinal weights used in the scheduling objective.

That workers value predictability and control is not merely inferred from well-being correlations; it is priced. A discrete-choice experiment embedded in a national call-centre hiring process finds that the average worker gives up 20% of wages to avoid an employer-set, short-notice schedule, and 8% for the option to work from home; most workers will not pay for scheduling flexibility, but a high-valuation tail is large enough to sustain sizeable compensating differentials [56]. Analytical work on workforce scheduling with heterogeneous time preferences explains why allocation policies that cannot express that heterogeneity depress both effective wages and worker supply [107]. Crucially, the resulting objective is not purely redistributive. A randomised experiment in 28 retail stores over nine months found that schedules redesigned for consistency, predictability, adequacy and employee input raised productivity by 5.1% and sales by 3.3% while reducing labour hours by 1.8% [59], so that worker-centred and firm-centred criteria are at least partly complementary rather than strictly antagonistic.

The burden of non-standard hours is not evenly shared. It falls on employees who must synchronise paid work with care obligations, and those obligations remain heavily gendered. Microdata for 134 countries show that the employment child penalty becomes the dominant driver of gender inequality as labour markets shift from subsistence agriculture towards salaried work [40], and Italian matched employer–employee data for 1985–2018 place the long-run child penalty in annual earnings at 52 log points, driven mainly by reduced weeks worked rather than by lower wage rates [41]. In the Chinese setting that motivates the instances used here, unpaid domestic and care work is divided unequally between partners and the imbalance varies systematically across parenthood stages [108]; motherhood carries a measurable earnings penalty that grandparental care only partly offsets, and the employment response runs through multi-generational household structure [44,91]; and parenthood contributes to the urban gender wage gap, the motherhood wage penalty exceeding the fatherhood one, although it narrowed between 2002 and 2018 [43]. Access to a local childcare centre cuts a mother’s own childcare time by 13.3 hours per week [85], but only if her shift pattern lets her use it, which is precisely the coupling between the two applications set out in Figure 13.

Personnel rostering is, by contrast, a mature operational-research problem. Nurse rostering is NP-hard and is routinely formulated so that employer demand and employees’ own requests are optimised jointly [69]; shift design has been extended to stochastic demand, which makes employer-driven volatility an endogenous modelling choice rather than an exogenous fact [109]; and human-factor constraints such as fatigue and minimum rest are increasingly placed inside the objective rather than applied as post hoc corrections [73]. Fairness and work–life balance have entered this objective set only recently: equity of shift allocation has been encoded as an explicit optimisation criterion [71], fairness has been recognised as intertemporal rather than within-period [72], general guidance now exists for formulating equity terms in allocation models [101], and work–life balance has been built directly into staff-scheduling models, including formulations that carry the worker’s physiological and psychological state [74,75,110]. A review of 52 studies on multi-objective personnel rostering documents alternatives to weighted-sum scalarisation and discusses scalarisation’s prevalence and limitations in the broader literature [111]. The computational studies motivate specific alternatives to the continuous search used here. Co-evolutionary NSGA-II [70] is relevant to multi-objective workforce planning; deep-Q-network-guided search selects neighbourhood moves [112]; shift-pattern learning constructs feasible work patterns [113]; biased random-key genetic search provides a representation for workforce scheduling [114]; and neural-assisted search supports heuristic selection and partial reconstruction [115]. Their objectives and representations differ from FFSS, so they supply design precedents rather than direct performance comparisons. Exact and hybrid methods remain relevant alternatives [111]; their performance on the present FFSS model is not established by this study.

The FFSS problem is therefore formulated below as a multi-objective roster design problem, in which the expected work–family conflict burden imposed by the assigned shift pattern, the inequality of that burden across employees, and the violation of the contract and consecutive-day rules are traded against coverage of demand. Following the prevailing practice documented above, the objectives are scalarised into a single weighted criterion, and the resulting high-dimensional, discontinuous search problem is solved by MSCPO; a representative solved roster is shown in Section 5.2.6.

#### 5.2.2. Model Formulation

A service unit employs E workers and must cover a horizon of D days with three shifts: morning (07:00–15:00), afternoon (15:00–23:00) and night (23:00–07:00). Shift s on day d requires $r_{ds}$ workers. Worker e has a contractual number of working days $H_{e}$, an availability indicator $a_{ed}$, a preference weight $\pi_{es}\in (0,1]$ for each shift, and a care-responsibility intensity $\kappa_{e}\in [0,1]$ that measures the presence and intensity of dependants in the household.

The decision vector is a matrix of random keys $X\in [0,1{]}^{E\times D}$, one priority per worker and day, so the search dimension is D=ED. A roster $A\in \{0,1,2,3{\}}^{E\times D}$, where zero denotes a day off, is produced by the deterministic decoder of Equation (38): for each day the shifts are filled in decreasing order of scarcity, and shift s is assigned to the $r_{ds}$ eligible workers with the highest score.

(38) $$\phi_{eds}=\left\{\begin{array}{c} x_{ed}\;\pi_{es},\;\;a_{ed}=1,e\;\mathrm{not}\;\mathrm{yet}\;\mathrm{assigned}\;\mathrm{on}\;\mathrm{day}\;d,n_{e}<H_{e}+1\\ -1,\;\;\mathrm{otherwise} \end{array}\right.$$

where $n_{e}$ is the number of days already assigned to worker e. Coupling the priority $x_{ed}$ with the preference weight $\pi_{es}$ means that the same key can send a worker to different shifts on different days, so the encoding spans the whole roster space while remaining continuous, which is what allows a real-valued optimiser to be applied to a combinatorial rostering problem. Residual understaffing, which can only arise from availability conflicts, is measured by Equation (39).

(39) $$U(X)=\frac{1}{\sum_{d,s}r_{ds}}\sum_{d=1}^{D}\sum_{s=1}^{3}max\left\{0,r_{ds}-\left|\left\{e:A_{ed}=s\right\}\right|\right\}$$

Five features of a roster are known to generate work–family conflict: night work, weekend work, quick returns, schedule instability and assignment to a disliked shift. They are counted by Equation (40).

(40) $$\begin{array}{c} n_{e}^{ngt}\;=\sum_{d}1\left[A_{ed}=3\right]\\ n_{e}^{wkd}\;=\sum_{d\in W}1\left[A_{ed}>0\right]\\ n_{e}^{qr}\;=\sum_{d}1\left[\Delta_{ed}<\Delta^{min}\right]\\ n_{e}^{ins}\;=\sum_{d}1\left[A_{ed}>0,A_{e,d+1}>0,A_{ed}\neq A_{e,d+1}\right]\\ n_{e}^{mis}\;=\sum_{d}\left(1-\pi_{e,A_{ed}}\right)1\left[A_{ed}>0\right] \end{array}$$

where W is the set of weekend days, $\Delta_{ed}$ is the rest interval between the end of the shift on day d and the start of the shift on day d+1, and $\Delta^{min}=11$ h is the minimum rest period. The individual work–family conflict burden aggregates the five counts, amplifies them by the worker’s care responsibilities and normalises by the contractual workload, as in Equation (41).

(41) $$W_{e}(X)=\frac{\left(\theta_{1}n_{e}^{ngt}+\theta_{2}n_{e}^{wkd}+\theta_{3}n_{e}^{qr}+\theta_{4}n_{e}^{ins}+\theta_{5}n_{e}^{mis}\right)\left(1+\lambda \kappa_{e}\right)}{H_{e}}$$

The multiplier $1+\lambda \kappa_{e}$ is the operational content of the term “family-friendly”: the same night shift is treated as a heavier burden when it falls on a worker with dependants. It is a normative weight, not an estimate, and Section 5.2.6 reports how the solution changes when it is switched off.

Two further quantities complete the objective. Distributional fairness is measured by the Gini coefficient of the burden across the workforce, Equation (42), so that a roster which protects carers by dumping every night shift on one childless worker is penalised. Rule violations aggregate deviations from the contract and runs of more than five consecutive working days, Equation (43).

(42) $$G(X)=\frac{\sum_{e=1}^{E}\left(2e-E-1\right)W_{\left(e\right)}}{E\sum_{e=1}^{E}W_{e}},W_{\left(1\right)}\leq W_{\left(2\right)}\leq \cdots \leq W_{\left(E\right)}$$

(43) $$R(X)=\frac{1}{E}\sum_{e=1}^{E}\left[\left|n_{e}-H_{e}\right|+\frac{3}{2}max\left\{0,l_{e}-5\right\}\right]$$

where $l_{e}$ is the longest run of consecutive working days assigned to worker e.

The scheduling problem solved by the optimiser is the minimisation of Equation (44).

(44) $${min}_{X\in [0,1{]}^{E\times D}}F_{FFSS}(X)=\omega_{1}U(X)+\frac{\omega_{2}}{E}\sum_{e=1}^{E}W_{e}(X)+\omega_{3}G(X)+\omega_{4}R(X)$$

The objective is a step function of the keys: it changes only when a key crosses a rank boundary in the decoder, so it is piecewise constant, has zero gradient almost everywhere and is riddled with plateaux. This is a deliberately unfriendly landscape for an optimiser designed on smooth benchmarks, and it is the reason the problem is included.

The components of the FFSS model are therefore the following. The decision variables are the ED random keys X; the objective is Equation (44). Three hard constraints hold by construction of the decoder of Equation (38): each worker takes at most one shift per day, works only on available days ($a_{ed}=1$) and is assigned at most $H_{e}+1$ working days. Coverage of demand, Equation (39), and the contract and the limit of five consecutive working days, Equation (43), are soft constraints, penalised in Equation (44) through $\omega_{1}$ and $\omega_{4}$. Equity is measured by the Gini coefficient of Equation (42), computed over the individual burdens $W_{e}$ of Equation (41); it is zero when every worker carries the same burden.

#### 5.2.3. Instances

Four instances of increasing size are generated. Demand is heavier on weekdays than at weekends and is split 40:36:24 across the morning, afternoon and night shifts. Sixty-eight per cent of the workforce is female, and care-responsibility intensity is drawn from distributions calibrated so that women carry the larger share of it, in line with the evidence on the gendered division of unpaid care reviewed in Section 5.2.1. Shift preferences fall with care intensity, most steeply for the night shift. Contracted working days are set so that total contracted supply equals total rostering demand, which makes the contract rule attainable and therefore informative. Quantitatively, the daily demand is $0.62E(1-0.12w_{d})$ workers, rounded, where $w_{d}=1$ on the two weekend days of each week and zero otherwise, and every shift receives at least one worker after the 40:36:24 split. Each worker is female with probability 0.68; care intensity is drawn from a Beta(2.4, 2.0) distribution for women and a Beta(1.3, 3.6) distribution for men, with means of 0.55 and 0.27; preference weights are drawn uniformly from [0.55,1] and then reduced by $0.45\kappa_{e}$ for the afternoon and $0.70\kappa_{e}$ for the night shift, with a lower bound of 0.05; each worker is available on each day with probability 0.94; and the contracted days $H_{e}$ divide the total demand as equally as possible. Table 20 summarises the four instances.

#### 5.2.4. Results

**Figure 16:**
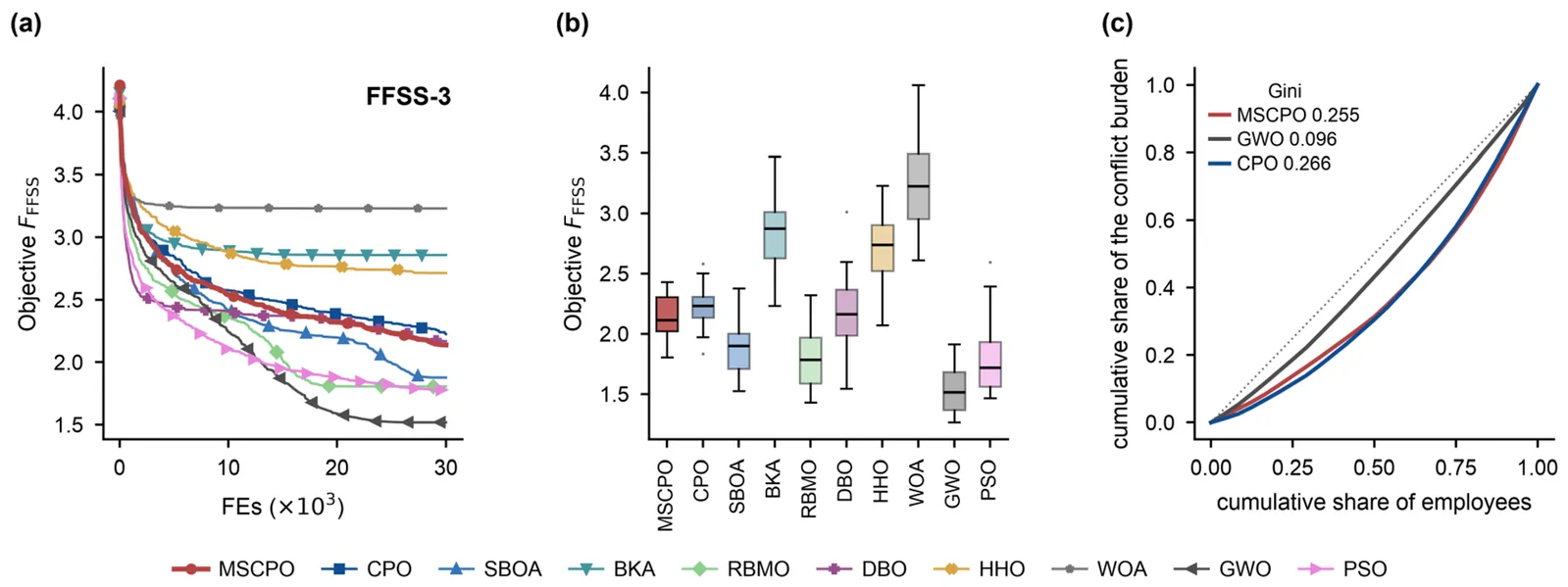
Performance on the FFSS problem (FFSS-3): (**a**) convergence curves; (**b**) objective distribution; (**c**) Lorenz curves of the conflict burden. The grey dotted diagonal indicates equal distribution of the conflict burden.

The scheduling problem is the harder of the two for a continuous optimiser, and the result is the one negative finding of this study: MSCPO does not win it. Table 21 reports the objective values, and Figure 16 reports the convergence curves and their dispersion. Panel (c) of that figure compares how evenly the conflict burden is shared across the workforce under the best rosters found. The full ordering by mean rank over the four instances is GWO 2.00, RBMO 2.50, PSO 3.00, SBOA 4.00, MSCPO 4.75, CPO 5.75, DBO 6.00, HHO 8.00, BKA 9.00, WOA 10.00. MSCPO is 5th, and the Wilcoxon tests do not separate it from its own base algorithm CPO (zero wins, four ties, zero losses). A Friedman test over four instances is reported for completeness ($\chi_{F}^{2}(9)=30.27$, p < 0.001), with the caveat that four blocks cannot separate adjacent algorithms; the per-instance Wilcoxon tests confirm the direction: against GWO MSCPO wins none of the four instances, ties on FFSS-1 and loses on the other three. The ordering is almost the reverse of the CEC2017 ordering of Table 7: the algorithms that led the benchmark trail here, and three algorithms that ranked in the middle of the benchmark—GWO, RBMO and PSO—lead. A result of that shape is informative rather than embarrassing, because it says something specific about the problem class, and the next subsection establishes what.

#### 5.2.5. Why the Benchmark Ordering Does Not Transfer

The obvious explanation—that the piecewise-constant objective traps a greedy optimiser on a plateau—is wrong, and the measurement in Table 22 shows why. Over five independent runs on FFSS-3, each algorithm was instrumented to record how many distinct rosters its decoder ever produced, how large a step an individual takes between consecutive iterations relative to the width of the search box, and how many of the 336 employee-day cells change between a candidate and the previous candidate at the same population size. MSCPO and CPO produce more distinct rosters than any other algorithm tested (29,727 and 29,741 respectively, about 99 per cent of all evaluations), so repeated evaluation of identical decoded rosters is not the main explanation for their lower solution quality. Their problem is the opposite. Its mean step is 0.401 of the box width against 0.124 for GWO, and each of its moves rewrites 163 of the 336 cells against 57 for GWO. In a rank-key encoding, the neighbourhood that matters is not a ball in key space but the set of rosters reachable by flipping a few key comparisons, and the keys of one employee-day are spaced roughly 1/E apart. A step of 0.40 crosses many of those gaps at once, so what is a local move in the search space is a near-global move in the decoded space: MSCPO makes large changes in decoded rosters, which may impede refinement under this representation. The three algorithms that win here all take steps that change between 36 and 57 cells, although DBO, which does the same, ranks lower.

These measurements indicate a limitation of MSCPO under the FFSS rank-key encoding tested here, and it is worth stating plainly because it is the kind of result that a benchmark suite cannot produce. The CEC2017 functions are continuous, so a small step in the decision vector is a small step in the objective, and every strategy in Section 3 is built on that correspondence: a well-spread initial sample covers the box, an elite guide points at a good region of the box, and truncating the population concentrates the budget on a good region of the box. Under a rank decoder, the correspondence breaks. The practical remedy is to match the step scale to the key spacing—by shrinking the operators’ step by a factor of order 1/E, or by hybridising with a permutation neighbourhood—and we leave that to future work rather than retrofitting it here, since an encoding-aware modification would need independent testing before its effectiveness could be established.

#### 5.2.6. Roster Quality

**Figure 17:**
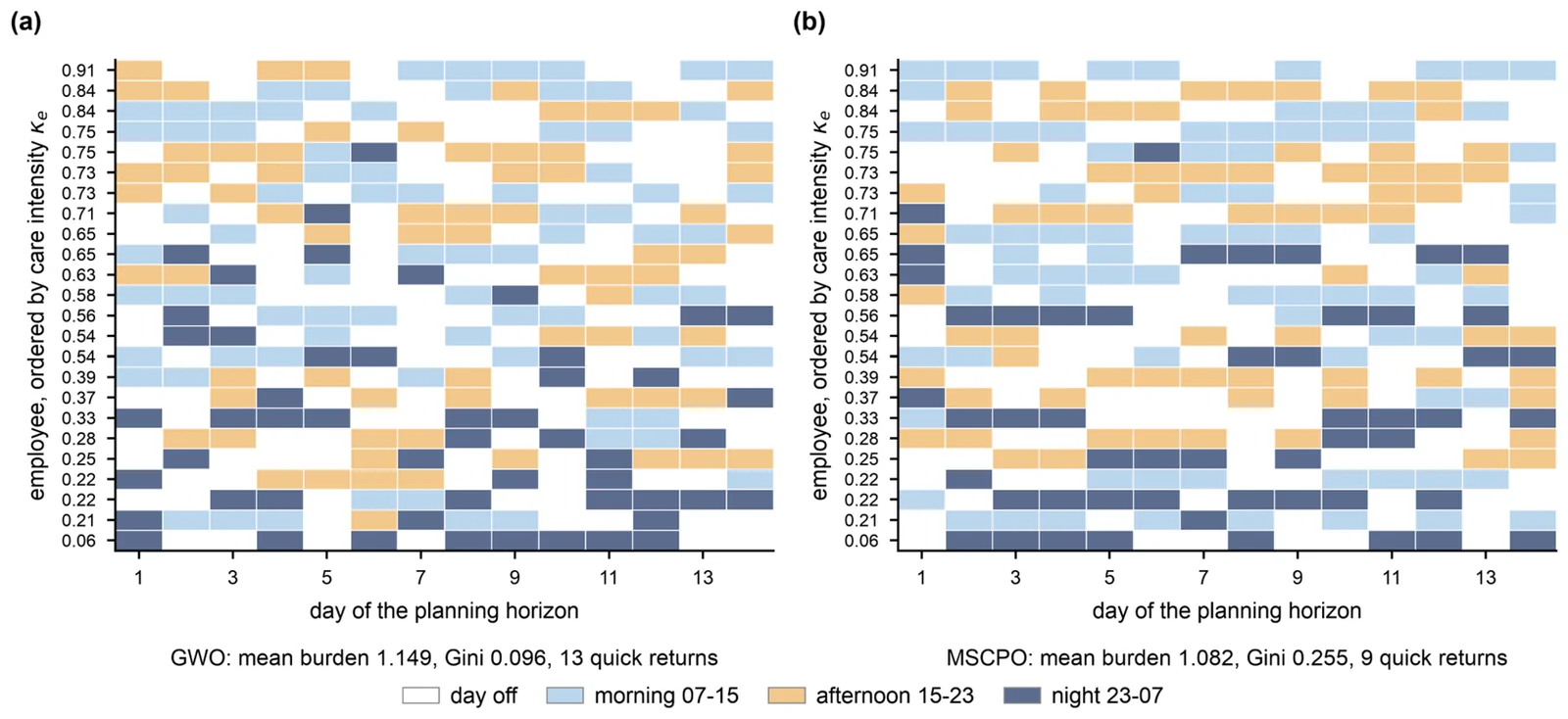
Best rosters found on FFSS-3: (**a**) GWO; (**b**) MSCPO.

What the objective actually does to a roster is nevertheless worth reading, and Table 23 and Figure 17 report it for the best roster found by GWO and for the best found by MSCPO on FFSS-3. The winning roster does not win on the average burden: its mean work–family conflict burden is 1.149 against 1.082, and it has 13 quick returns—rest intervals shorter than the eleven hours of Equation (40)—against nine. What it wins on is how that burden is spread and whether the contract and consecutive-day rules hold: the Gini coefficient of the burden is 0.096 against 0.255, and the winning roster has no violation of the rules of Equation (43), against 0.167 per worker. Nor does it buy that from the employees without dependants: in the GWO roster the burden of employees with $\kappa_{e}\geq 0.5$ is 1.097 against 1.235 for the remainder. The roster diagram makes the mechanism legible: night shifts migrate towards the employees with the lowest care intensity and the blocks of consecutive identical shifts grow longer, which is what holds the dispersion and the violation count down at the same time. That behaviour is a property of the objective of Equation (44), not of the optimiser that found the solution, and it is the part of the result that transfers to practice.

The mean burden and the objective do not have to move together, and Table 23 shows that they do not: in three of the four instances, the MSCPO roster has the lower mean burden even though GWO attains the better objective. The objective of Equation (44) is a weighted sum of four terms, and the mean burden is only one of them, so a roster can be better on the average burden and worse on its dispersion, on the number of quick returns or on the rules of Equation (43). That is a property of the scalarisation, and it is one of the reasons the multi-objective extension discussed in Section 6.4 and Section 7 is the natural next step rather than an optional refinement.

Two qualifications belong with this result. The first is that the amplification factor $1+\lambda \kappa_{e}$ of Equation (41) is a normative choice: the planner has decided that an hour of night work costs more when there is a dependent at home. To check that the redistribution visible in Figure 17 is produced by that choice rather than by the optimiser, FFSS-3 was resolved with λ=0, which turns the problem into a conventional fairness-constrained roster, in 12 independent runs of GWO and of MSCPO at each of the two values of λ, separate from the 30 runs behind Table 21. Employees with $\kappa_{e}\geq 0.5$ make up 62 per cent of this workforce. With λ=1.6 they receive on average 34 per cent of the night shifts under GWO and 35 per cent under MSCPO—far below their share of the workforce. With λ=0 these averages rise to 50 and 40 per cent, and under GWO the gap between the mean burden of carers and of the remainder closes from 1.117 against 1.221 to 0.689 against 0.739. The protection is therefore an output of the objective, and switching it off recovers something close to a proportional allocation of unsocial hours.

The second qualification is that no schedule can create staffing capacity. The gains reported here come from re-allocating a fixed set of shifts and are bounded by the number of employees with low care intensity available to absorb the unsocial hours; even at λ=1.6, carers still take about a third of the night shifts, because there are not enough non-carers to take the rest. Where that margin is exhausted, the only remaining instruments are hiring and the shift structure itself, neither of which this model varies. A third point is worth making for honesty rather than qualification: the burden index of Equation (41) is an index, not a measured quantity. Its five weights are chosen to reflect the ordering that the work–family literature reports—night work worst, then quick returns, then weekend work, then instability and preference mismatch—but the literature does not supply cardinal weights, and a different defensible set would change the rosters the optimiser prefers.

## 6. Discussion

### 6.1. Comparison with Other Enhanced CPO Variants

The factorial ablation clarifies how MSCPO relates to other component designs. Although initialisation is modified in 29 of the 43 CPO variants reviewed in [20], SQI contributes about 5% of the total main effect under the present protocol. The targeted-defence variant [25] likewise reports no improvement from chaotic opposition initialisation alone, while the comparative study [28] finds that initialisation effects depend on the algorithm and problem. These comparisons caution against attributing a variant’s gain to its starting sample; they do not establish that initialisation is generally unimportant for CPO. EGE contributes about 70% of the total main effect among the three tested changes. Its relationship to JADE [30] is that it is guided by a sample from a top-ranked pool. Its relationship to comprehensive-learning PSO [29] is the use of more than one good exemplar, without importing personal-best memory. Recent diversity-guided PSO [81] controls attraction, repulsion and local learning, and diversity-enhanced DE [82] also employs restart mechanisms; MSCPO instead leaves these controls outside its design. Archive-based DE combines nonlinear population reduction, archive adaptation and selective pressure [83], while APR fixes the schedule shape and changes the terminal population and survivor selection. These mechanisms differ from MSCPO, but the present ablation estimates the contributions of SQI, EGE and APR only; it does not compare performance with those algorithms.

Table 1 compares designs and published evaluation protocols, rather than the empirical robustness of enhanced CPO variants. SDHCPO [21] modifies initialisation, parameter control and crossover; CAPCPO [23] adds mutation and adaptive population adjustments; ECPO [24] augments guidance and perturbation; and the targeted-defence variant [25] makes targeted-defence changes. Their multiple interventions make component ablation relevant to attributing gains. Some reports use iteration budgets without stating how extra evaluations are counted [21,23,24]. MSCPO uses a common FE budget including initialisation, a factorial ablation and Holm-corrected post hoc comparisons. The dispersion and worst-run measures in Section 4.8 support robustness comparison with the nine algorithms actually tested; they do not establish superiority over the enhanced variants in Table 1. The need to compare performance across FE budgets [78] is evident here: MSCPO’s advantage over CPO disappears when the budget increases (Section 4.7), so iteration-budget results cannot be treated as a common-budget robustness ranking. Finally, SDHCPO and HCPO motivate repairs through diversity preservation [21,22], whereas MSCPO improves on CPO without greater measured diversity (Section 4.5). This supports productive guidance under the tested conditions, rather than a general diversity-preservation mechanism.

### 6.2. Comparison with the Facility-Location and Family-Policy Literature

On the childcare problem, the benchmark ordering carried over, and the substantive results can be set against two studies. The fairness-covering formulation [65] and service-allocation analysis [66] provide precedents for the access-dispersion term in CFLC. An equity-driven robust location model extends this issue to uncertainty [100], which the present deterministic calibration does not address. Guidance on fairness formulations also makes the comparison baseline important [101]: this study did not compute the loss relative to an optimum without an equity term. Relative to the siting rules, the optimised programme maintains employment while improving access dispersion: the most equal rule on each instance leaves the CV of accessibility 39 to 55 per cent above that of the optimised programme (Table 19), while the optimised programme induces at least 99 per cent of the employment of the best rule. The optimised programmes direct capacity to the least-provided zones (Section 5.1.4), and in these instances doing so costs little employment, so equity and efficiency conflict only weakly until the under-provided periphery has been served, which is also why the inequality of access becomes U-shaped in the budget. In the family-policy literature, the closest counterpart is a childcare-allocation study [60], in which siting rules based on neighbourhood income or excess demand achieve about 60 per cent of the employment gains of the optimal allocation of new centres in São Paulo, whereas the four sensible rules of Table 19 achieve 93 to 101 per cent of the employment of the optimised programme in the calibrated instances. The difference concerns the model rather than the optimiser. Apart from its dependence on baseline participation, the response slope of Equation (33) is uniform across zones, whereas the childcare-allocation study estimates responses that differ across neighbourhoods and families [60], and the evidence summarised in Table 13 shows that responses do vary with baseline provision, participation and household structure. If the response also varied across zones in ways that simple siting rules do not track, an optimiser using zone-level slopes could outperform the rules by more than it does here; if instead the response is largest where under-three provision is low relative to mothers’ attachment to the labour market, as the evidence of Table 13 suggests, the provision-gap rule of Table 19 already captures part of that heterogeneity. Which effect dominates can be settled only with zone-level estimates of the response (Section 6.4). The absolute employment gains are a separate matter: they rest on a slope above the German and Japanese estimates (Section 5.1.2), and they would shrink in general equilibrium (Section 7). The optimiser’s preference for provision gaps over employment gaps also agrees with the cross-country finding that childcare expansion raises maternal labour supply where the gap between maternal participation and coverage is large [50].

### 6.3. Comparison with the Rostering Literature

In integrated operating-room scheduling, several search engines share a random-key decoder and are combined with shaking and neighbourhood descent [116]. The decoder sorts the keys before constructing a schedule, linking the representation to subsequent local search. Its operating-room objective differs from FFSS, and its numerical results are not transferred here. In workforce scheduling, the biased random-key method [114] is the closest representation precedent; the deep-Q-network study [112] guides neighbourhood search, shift-pattern learning [113] changes work patterns, and neural-assisted rostering [115] supports selection or reconstruction of solutions. These studies motivate testing discrete refinement around the present continuous updates. In Table 22, the three winning algorithms change between 36 and 57 roster cells between successive decoded candidates, compared with about half the roster for MSCPO and CPO; DBO makes similarly small changes but ranks lower. The measurements suggest an encoding-related limitation, without establishing that step size alone causes the ordering. On the substantive side, the winning GWO rosters have burden Gini coefficients between 0.06 and 0.14, against 0.14 to 0.27 for MSCPO and CPO (Table 23). Fairness-oriented crew rostering [71] motivates within-period equity; fairness over time [72] points to a multi-period extension beyond the present model. Whether these rosters preserve productivity, as the retail experiment [59] found for more predictable schedules, remains outside this model.

### 6.4. Directions for Further Research

The findings point to six directions for further research, several of which would also remove the limitations summarised in Section 7. First, budget-aware schedules: since the advantage of MSCPO over CPO disappears when evaluations are plentiful, and Section 4.3 traces the contribution of APR to the size of the late population rather than to the shape of the schedule, the terminal population size could be tied to the remaining budget, switching from monotone reduction to restarts or re-expansion once the truncated population can no longer use the budget productively, and tested at several FE budgets, following multi-budget benchmarking guidance [78], to determine whether the short-budget gain can be retained while longer runs improve. Second, encoding-aware step control: for random-key and rank-key decoders, the step of each operator should be scaled to the spacing of the keys, of order 1/E in the scheduling problem, or the continuous operators hybridised with swap or permutation moves; the common-decoder and neighbourhood-search design used in operating-room scheduling [116] motivates testing these changes with the same FFSS decoder and FE accounting, rather than assuming that they restore the benchmark ordering. Third, a multi-objective form: both applications resolve trade-offs between output, cost and equity, or between coverage, conflict and fairness, through fixed weights, and non-dominated selection in multi-objective CPO [79] and reference-point guidance [80] provide alternative archive designs, while the rostering review [111] motivates eliciting planner preferences. A future extension should approximate the attainable trade-offs and test sensitivity to those preferences. Fourth, heterogeneous and uncertain responses: the response slope should be estimated zone by zone from administrative data and treated as uncertain, so that siting is optimised for its expected value or its worst case over the range of estimates in Table 13; the recalibration of Table 18 shows that this uncertainty affects the scale of the optimal programme far more than its shape. Fifth, coupling the two levers: a nursery place is useful only if a parent’s shifts fall within its opening hours, and a joint, possibly bi-level, model in which a planner sites centres and employers roster workers around them would capture the link sketched in Figure 13. Sixth, dynamic instances built from real data: a multi-period version in which capacity is added over several budget years as cohorts of children change, solved on the zone-level data of a Chinese city within the fifteen-minute community life circle [97], would test whether the regularities found in the calibrated instances hold in practice. Two algorithmic tasks also remain: a convergence analysis under a time-varying population, which the systematic review [20] identifies as missing for CPO in general, and a comparison with the strongest published CPO variants under a common, fully budgeted protocol.

## 7. Conclusions

This paper set out to do two things: to repair three identifiable defects of CPO, and to show that a bio-inspired optimiser can be put to work on the allocation problems that govern female labour supply. The proposed MSCPO keeps CPO’s four defence mechanisms, its trade-off constants and its greedy acceptance rule exactly as published, and changes only how the population is started, which individuals guide the exploitation operators and how the population size evolves. Because the operator set is untouched, the full factorial ablation of Section 4.3 attributes the improvement to those three changes and to nothing else.

The benchmark evidence is consistent across three independent lines. On the complete CEC2017 suite MSCPO attains the best Friedman mean rank at all four dimensions and 1.29 overall, and a Holm-corrected post hoc test with MSCPO as control rejects equality against every one of the nine comparison algorithms once the four dimensions are pooled, the largest adjusted p-value being 0.00193; taken one dimension at a time the same procedure separates MSCPO from seven or eight of the nine but not from CPO, and it is the function-level Wilcoxon tests—81 wins, 34 ties and one loss against CPO over the 116 function–dimension pairs, or 57 wins and no loss after a Holm adjustment—that carry that particular comparison. The margin grows with dimension, and the instrumentation of Section 4.5 identifies the mechanism: MSCPO does not hold more diversity than CPO; it converts a comparable amount of diversity into more late-stage improvements. The factorial ablation points at the same component, and it also quantifies the contribution: on the mean Friedman rank, the elite-guided phase accounts for about seventy per cent of the total main effect, the population schedule for a quarter, a share that an additional variant traces to the smaller late population rather than to the monotone shape of the schedule, and the initialisation for the remainder, and the three act additively rather than synergistically. MSCPO is also the most robust of the ten algorithms, with the smallest run-to-run dispersion and the best worst-case error on most function–dimension pairs (Section 4.8). Section 4.7 bounds the claim: at the full CEC2017 budget of $10^{4}D$ evaluations the advantage over SBOA and RBMO persists, although it narrows, while the advantage over CPO disappears and at D=30 reverses. The three strategies buy convergence speed, and convergence speed is worth having exactly when evaluations are scarce.

The two applications answer different questions, and it is worth keeping them apart. On the CFLC problem, MSCPO ranks first, and the gain is economically legible rather than merely numerical: on the largest instance it converts the same annual budget into an allocation whose dispersion of access is a fraction of what the siting rules of a planning department would produce, at a negligible cost in aggregate employment, and the optimised solutions reproduce two policy regularities—capacity is directed towards the least-provided zones rather than the lowest-participation ones, and the spatial inequality of access is U-shaped in the budget—that the model was not told to produce. On the FFSS problem, MSCPO does not rank first; GWO does, and the benchmark ordering is close to reversed. Instrumentation on FFSS-3 suggests that the rank-key representation contributes to the reversed ordering: under a rank-key decoder, MSCPO’s moves rewrite about half of the roster at once, so the algorithm makes large changes in the decoded roster, which may hinder local refinement. This result motivates checking how changes in the decision vector affect the decoded objective before transferring the strategies of Section 3 to a new representation. Step scaling and discrete neighbourhood moves remain hypotheses requiring independent evaluation.

Five limitations qualify these results and point to the work that should follow. First, and most important, the advantage of MSCPO over CPO is specific to the evaluation budget: Section 4.7 shows that it disappears at the full CEC2017 budget and at D=30 reverses, so the three strategies should be understood as buying convergence speed rather than final quality, and recommended only where evaluations are scarce. Second, the scheduling result of Section 5.2 is a limitation of the proposed algorithm on rank-key encodings, and the obvious remedy—matching the operators’ step scale to the spacing of the keys, or hybridising with a permutation neighbourhood—has deliberately not been applied here. Third, both applications are single-objective: the trade-offs between output, cost and equity in Equation (37) and between coverage, conflict and fairness in Equation (44) are resolved by fixed weights chosen ex ante. A planner would rather see the Pareto front, and extending MSCPO to a multi-objective form with an external archive and non-dominated sorting is the natural next step. Fourth, the instances are calibrated simulations rather than administrative data. The geography, the cost structure and the behavioural slope are all taken from published sources, the slope being transferred from West German and Japanese estimates because none in the same unit was found for China, and the generators are available from the corresponding author on request so that every number can be reproduced or replaced, but the step from a calibrated city to a real one requires zone-level counts of children, employment and travel times that are held by municipal authorities. Fifth, the labour-supply response of Equation (33) treats the reduced-form elasticity as a structural parameter and holds it fixed while the programme changes the very quantity that identifies it; a general equilibrium in which wages and the price of private childcare adjust would attenuate the estimated gains; the recalibration reported in Section 5.1.4 (Table 18) shows how strongly the scale of the programme depends on the slope. Relaxing any of the five is a research programme rather than a revision, and each of them is more likely to change the magnitude of the gains than their ordering across algorithms.

Beyond those extensions, the wider point of the paper is methodological. Two studies have been solving halves of the same problem. Labour and family economics has spent two decades estimating, carefully and credibly, how much childcare access and schedule quality matter; swarm intelligence has spent the same two decades learning to allocate scarce resources over combinatorial spaces, and testing itself almost exclusively on mechanical design problems. The elasticities that the first literature produces are exactly the coefficients the second needs inside an objective function, and the allocations that the second produces are exactly the counterfactuals the first can only evaluate after the fact. Bio-inspired optimisation has a great deal to contribute to family policy, and family policy has a great deal to offer as a class of test problems that are large, constrained, discontinuous and genuinely unsolved.

## Figures and Tables

**Table 1:** Design of MSCPO compared with the base algorithm and published enhanced CPO variants.

Variant	Initialisation	Defence Operators	Exploitation Guide	Population Schedule	Evaluation
CPO [14]	Uniform random	Four defences, Equations (3)–(12)	Random individual (odour); incumbent best (attack)	Cyclic, N to 0.8N in this study	-
CAPCPO [23]	Uniform random	Cauchy jumps replace defences 1 and 3 when fitness diversity is high; adaptive factor in defence 4	Incumbent best (defences 3 and 4)	Switch between N and 0.5N by fitness diversity; random reset	500 iterations; $2^{3}$ ablation; Friedman, Wilcoxon
ECPO [24]	Sobol	Defence 2 guided by the best; adaptive Levy step in defence 4; centroid opposition every iteration	Incumbent best (defences 2 and 4)	Cyclic, kept	500 iterations; single-strategy curves; no significance test
SDHCPO [21]	Sobol + opposition of the worse half	DE/rand/1 in defence 1; horizontal-vertical crossover in defence 2; cosine-annealed weight in defence 4	Incumbent best (defence 4)	Cyclic, kept	500 iterations; one-on and one-off ablation; Friedman, Wilcoxon
HCPO [22]	Sobol + Logistic chaos	Cellular-automata neighbourhood, hunting impulse, sinusoidal step reduction, crossover	Neighbourhood regulating elite-information diffusion (guide vector not stated)	Not stated in the abstract	CEC2017, CEC2022 (details not accessible)
Fan et al. [25]	Kent chaos + opposition, pairwise	Defences 1, 2 and 4 replaced; defence 3 unchanged	Random peer (defence 4); best only in defence 2	Cyclic, kept	1000 iterations; $2^{4}$ ablation; Friedman, Wilcoxon, Nemenyi
BTCPO [27]	Uniform random	Four defences kept; extra butterfly-search or triangular-walk step	Incumbent best (extra step and defence 4)	Cyclic, kept	Descriptive statistics only
ICPO [19]	Good point set	Audiovisual defence redesigned (as reported in [20])	Not reported in [20]	Removed	UAV path planning
MSCPO (this study)	Scrambled Sobol + quasi-opposition; N fittest of 2N	Four defences kept; only the guide of Equations (10) and (12) changes	Elite pool of $\left\lceil pN_{t}\right\rceil$ individuals (defences 3 and 4)	Monotone linear N to 0.5N, truncation by fitness	Exact FE budget; $2^{3}$ ablation; Friedman, Holm, Nemenyi, Wilcoxon; full-budget check

Entries summarise the published descriptions; for HCPO [22] only the abstract was accessible, and ICPO [19] is described as reported in the systematic review [20]. Defences 1–4 are sight, sound, odour and physical attack. CAPCPO, ECPO, SDHCPO and the targeted-defence variant [25] use populations of 30 with 500 or 1000 iterations; the targeted-defence study [25] reports that its variant consumes about 27,060 evaluations per run, whereas CAPCPO, ECPO and SDHCPO do not state how extra evaluations are counted; the population and budget of BTCPO, HCPO and ICPO are not stated in the sources read.

**Table 2:** Parameter settings, population sizes, initialisation procedures and stopping criteria of MSCPO and the comparison algorithms.

Algorithm	Year	Population Size	Initialisation	Algorithm-Specific Parameters
MSCPO (proposed)	-	N=30, reduced linearly to $N_{min}=15$ (ρ=0.50)	Scrambled Sobol points and their quasi-opposites; N fittest of 2N kept (N extra FEs)	p=0.20, elite pool of $max(2,\lceil pN_{t}\rceil )$ individuals; α=0.2; $T_{f}=0.8$
CPO	2024	N=30; working size cycles between 24 and 30 ($N_{min}=0.8N$, $T_{c}=2$)	Uniform random, Equation (1)	α=0.2; $T_{f}=0.8$
SBOA	2024	N=30 (two FEs per individual per iteration)	Uniform random, Equation (1)	Levy exponent β=1.5, Levy step scaled by 0.5; stages switch at T/3 and 2T/3; escape threshold r=0.5; K∈{1,2}
BKA	2024	N=30 (two FEs per individual per iteration)	Uniform random, Equation (1)	p=0.9; attack step $n=0.05exp(-2(t/T{)}^{2})$
RBMO	2024	N=30 (two FEs per individual per iteration)	Uniform random, Equation (1)	small groups of 2–5; large group of 10 (the published range 10 to N/5 is empty for N<50); small or large group chosen with probability 0.5; $CF=(1-t/T{)}^{2t/T}$
DBO	2023	N=30 (roles 6:6:7:11)	Uniform random, Equation (1)	k=0.1; b=0.3; S=1 on the mean of the two distances of the thief update, as in the released code; dancing with probability 0.1; α=+1 with probability 0.9
HHO	2019	N=30 (variable FEs per iteration)	Uniform random, Equation (1)	$E_{0}\in [-1,1]$; Levy exponent β=1.5, scale 0.01
WOA	2016	N=30	Uniform random, Equation (1)	a: 2 to 0; $a_{2}$: −1 to −2; spiral b=1
GWO	2014	N=30	Uniform random, Equation (1)	a: 2 to 0
PSO	1995	N=30	Uniform random positions; velocities uniform in $\left[-v_{max},v_{max}\right]$	w: 0.9 to 0.4; $c_{1}=c_{2}=2.0$; $v_{max}=0.2(ub-lb)$

Settings common to all algorithms. Stopping criterion: exhaustion of the FE budget, with no other termination rule. Budget: 30,000 FEs (1000 iterations of N=30) in Section 4.1, Section 4.2, Section 4.3, Section 4.4, Section 4.5 and Section 4.6 and in both applications of Section 5, and $10^{4}D$ FEs (300,000 at D=30 and 999,990 at D=100) in Section 4.7. Independent runs: 30 per algorithm and function–dimension pair or instance (15 in Section 4.5, 10 in Section 4.7; in Section 5, 12 for the slope recalibration of Section 5.1.4 and the λ=0 check of Section 5.2.6, 10 per budget level for the budget sweep of Section 5.1.4 and 5 for the step-size diagnosis of Section 5.2.5), each with its own seed. Search domain $[-100,100{]}^{D}$ on CEC2017 and $[0,1{]}^{D}$ in both applications; coordinates leaving the box are clipped to it; FEs spent on initialisation are charged to the budget. t and T are the consumed and total numbers of FEs, so every time-dependent parameter follows the consumed share t/T of the budget. Uniform random initialisation: x=lb+r⊙(ub−lb) with $r∼U(0,1{)}^{D}$.

**Table 3:** Friedman mean ranks of MSCPO for four values of the elite rate p on the CEC2017 suite (α=0.05).

Variant	D = 30	D = 100	Overall	Rank
***p* = 0.10**	**2.31**	**2.00**	**2.16**	**1**
*p* = 0.15	2.48	2.21	2.34	2
*p* = 0.20	2.48	2.66	2.57	3
*p* = 0.30	2.72	3.14	2.93	4

Friedman test over the 29 functions at both dimensions (n=58 blocks, k=4 settings): $\chi_{F}^{2}(3)=11.61$, p=0.009; Iman–Davenport $F_{ID}(3,171)=4.07$, p=0.008. The row of the best setting is in bold. Post hoc tests with the default p=0.20 as the control: all Holm-adjusted p≥0.25. The Nemenyi critical difference is CD=0.62, and only p=0.10 and p=0.30 differ by more than it.

**Table 4:** Friedman mean ranks of MSCPO for four values of the reduction ratio ρ on the CEC2017 suite (α=0.05).

Variant	D = 30	D = 100	Overall	Rank
**rho = 0.3**	**2.07**	**1.90**	**1.98**	**1**
rho = 0.4	2.66	2.07	2.36	2
rho = 0.5	2.48	2.97	2.72	3
rho = 0.6	2.79	3.07	2.93	4

Friedman test over the 29 functions at both dimensions (n=58 blocks, k=4 settings): $\chi_{F}^{2}(3)=18.19$, p<0.001; Iman–Davenport $F_{ID}(3,171)=6.65$, p<0.001. The row of the best setting is in bold. Post hoc tests with the default ρ=0.5 as the control: Holm-adjusted p=0.006 for ρ=0.3 and p≥0.26 for the other settings. The Nemenyi critical difference is CD=0.62; it separates ρ=0.3 from ρ=0.5 and from ρ=0.6.

**Table 5:** The eight variants of the MSCPO ablation study.

Variant	SQI	EGE	APR	Description
V1	x	x	x	The base algorithm CPO
V2	yes	x	x	SQI only
V3	x	yes	x	EGE only
V4	x	x	yes	APR only
V5	yes	yes	x	SQI + EGE
V6	yes	x	yes	SQI + APR
V7	x	yes	yes	EGE + APR
V8	yes	yes	yes	the proposed MSCPO

SQI, Sobol quasi-opposition initialisation (Section 3.1); EGE, elite-guided exploitation (Section 3.2); APR, adaptive population reduction (Section 3.3); yes, strategy enabled; x, strategy disabled.

**Table 6:** Friedman mean ranks of the eight ablation variants on the CEC2017 suite (α=0.05).

Variant	D = 30	D = 100	Overall	Rank
V1	6.45	7.07	6.76	8
V2	5.86	6.86	6.36	7
V3	3.72	3.41	3.57	4
V4	5.52	5.41	5.47	6
V5	3.66	3.21	3.43	3
V6	5.07	5.62	5.34	5
V7	3.03	2.28	2.66	2
**V8**	**2.69**	**2.14**	**2.41**	**1**

Friedman test over the 29 functions at both dimensions (n=58 blocks, k=8 variants): $\chi_{F}^{2}(7)=193.14$, p<0.001; Iman–Davenport $F_{ID}(7,399)=51.72$, p<0.001. The row of the best variant is in bold. Post hoc tests with V8 as the control: Holm-adjusted p=0.60 for V7, 0.051 for V5, 0.033 for V3 and p<0.001 for V1, V2, V4 and V6. With CD=1.38 the Nemenyi test separates V8 from V1, V2, V4 and V6 but not from V3, V5 or V7.

**Table 10:** Wilcoxon rank-sum test results of MSCPO versus the comparison algorithms on the CEC2017 suite (α=0.05).

MSCPO vs.	D = 10	D = 30	D = 50	D = 100	Total
CPO	16/13/0	20/9/0	22/6/1	23/6/0	**81/34/1**
SBOA	22/1/6	23/5/1	24/4/1	26/3/0	**95/13/8**
BKA	29/0/0	29/0/0	29/0/0	28/1/0	**115/1/0**
RBMO	22/1/6	26/1/2	28/1/0	29/0/0	**105/3/8**
DBO	26/3/0	29/0/0	29/0/0	29/0/0	**113/3/0**
HHO	29/0/0	29/0/0	29/0/0	29/0/0	**116/0/0**
WOA	29/0/0	29/0/0	29/0/0	29/0/0	**116/0/0**
GWO	29/0/0	29/0/0	29/0/0	27/1/1	**114/1/1**
PSO	25/2/2	28/1/0	27/2/0	28/1/0	**108/6/2**
**All**					**963/61/20**

Entries give wins/ties/losses of MSCPO against the algorithm in the row at the unadjusted two-sided 0.05 level, over the 29 functions in each dimension column, over the 116 function–dimension pairs in the Total column and over all nine algorithms in the All row. The aggregate counts in the Total column and the All row label are in bold.

**Table 11:** Friedman mean ranks of the four strongest algorithms at the short and the full CEC2017 budget, with full-budget Wilcoxon outcomes (D=30, D=100).

D	Algorithm	Rank at 30,000 FEs	Rank at $10^{4}D$ FEs	MSCPO w/t/l at $10^{4}D$ FEs	Holm Adjusted p
30	MSCPO	1.31	2.03	-	-
	CPO	2.31	1.66	3/20/6	0.263
	SBOA	2.86	2.93	14/12/3	0.016
	RBMO	3.52	3.38	13/12/4	<0.001
100	MSCPO	1.14	1.76	-	-
	CPO	2.21	2.03	5/23/1	0.416
	SBOA	2.83	3.00	17/10/2	0.001
	RBMO	3.83	3.21	15/10/4	<0.001

The Wilcoxon column gives wins/ties/losses of MSCPO against the algorithm in the row over the 29 functions at the unadjusted two-sided 0.05 level. Holm-adjusted p is for the difference in Friedman mean rank from MSCPO at the full budget. Friedman test at the full budget (n=29 blocks, k=4): $\chi_{F}^{2}(3)=32.88$ at D=30 and $\chi_{F}^{2}(3)=26.38$ at D=100, both p<0.001.

**Table 12:** Robustness indicators of MSCPO and the comparison algorithms on the CEC2017 suite.

Algorithm	Mean Rank of SD	Pairs with Smallest SD	Mean Rank of Worst Error	Pairs with Smallest Worst Error	SD of per-Pair Rank	Worst per-Pair Rank	Pairs in Top Three (%)
MSCPO	**1.76**	**63**	**1.38**	**80**	0.53	**4**	**99.1**
CPO	2.19	38	2.25	25	1.64	8	87.1
SBOA	3.17	9	2.97	8	0.86	6	83.6
BKA	6.42	0	6.36	1	1.57	8	4.3
RBMO	4.29	3	4.15	2	1.09	8	13.8
DBO	6.97	0	6.59	0	1.39	8	1.7
HHO	8.76	1	9.69	0	0.36	10	0.0
WOA	8.79	0	9.28	0	0.32	10	0.0
GWO	6.16	2	6.06	0	1.26	10	3.4
PSO	6.47	0	6.27	0	1.44	8	6.9

Computed over the 116 function–dimension pairs of Section 4.4 (30 runs each). SD, standard deviation of the 30 final errors; worst error, the largest of the 30 final errors; per-pair rank, the rank of the algorithm’s mean error on a pair (1 = best). Friedman tests over the ten algorithms: $\chi_{F}^{2}(9)=730.26$ for the SD and $\chi_{F}^{2}(9)=890.99$ for the worst error, both p<0.001. The best value in each of the columns 2–5, 7 and 8 is in bold.

**Table 13:** Principal empirical estimates of the effect of childcare on maternal labour supply quoted in Section 5.1.1.

Study	Setting	Period	Design	Estimate as Reported
[47]	West Germany, under-3 expansion	2007–2014	County panel, county and year fixed effects	+0.2 pp participation per pp of slots
[48]	Japan, capacity for ages 0–5	2015–2017	Municipality panel	+0.27 pp probability of working per pp of capacity
[49]	Nicaragua, poor urban neighbourhoods	2013–2015	Neighbourhood-randomised roll-out, IV	+12 pp mothers’ work (effect of attendance)
[50]	Seven EU countries	2005–2012	Harmonised eligibility-cut-off IV	Positive in AT, CZ, HU, SK; null in FR, GR, IT
[51]	England, part-time vs. full-time	2000–2013	Birth-date RD; parent fixed effects	Part-time: no RD effect, +2.1 pp participation by the third term in the panel; full-time: +5.7 pp after one year
[88]	Uganda, childcare subsidy	2018–2020	Individual RCT	Single mothers: labour supply and earnings up; partnered mothers: no effect
[84]	United States, interstate movers	2011–2015	Multilevel logit (observational)	18% lower odds of employment where care is dearer
[85]	China (CHNS)	2004–2011	Fixed-effect SUR of time use	Tuition doubled: −1.3 h/week market work; centre access: −13.3 h/week care time
[89]	Austria, nursery and pre-school expansions	1990s–2000s	Event-study DiD	No effect on women’s earnings relative to men’s

pp, percentage points; IV, instrumental variables; RD, regression discontinuity; RCT, randomised controlled trial; SUR, seemingly unrelated regression; DiD, difference-in-differences; CHNS, China Health and Nutrition Survey; AT, Austria; CZ, Czech Republic; HU, Hungary; SK, Slovak Republic; FR, France; GR, Greece; IT, Italy. Periods are those of the data used for the estimate ([50]: sample of the working-paper version; [89]: childcare expansion events within data for 1953–2017).

**Table 14:** Components of the CFLC model.

Component	Specification	Where Defined
Decision variables	$z\in [0,1{]}^{J}$, one continuous key per candidate site; site j opens ($y_{j}=1$) if $z_{j}\geq 0.5$ and then receives $q_{j}$ places between $q_{min}$ and $q_{j}^{max}$, rounded to the nearest ten	Equations (28) and (29)
Objective	Minimise $F_{CFLC}$: minus the net market value of induced maternal employment (V−C), plus the equity penalty μCV, plus the budget penalty	Equations (34)–(37)
Hard constraints (by decoding)	$0\leq z_{j}\leq 1$; $q_{j}=0$ at closed sites; $q_{min}=30\leq q_{j}\leq q_{j}^{max}$ at open sites; places in multiples of 10; employment rate $e_{i}\leq \bar{e}=0.92$	Equations (28), (29) and (33)
Soft constraint	Annual public cost C(z)≤B, with penalty weight P=50 on the relative overrun	Equation (37)
Geography	Monocentric circular city of radius 18 km; zones and candidate sites at random positions; travel time = Euclidean distance × 1.30/22 km/h; catchment radius $d_{0}=30$ min	Section 5.1.3
Demand	$m_{i}$ mothers of a child aged 0–3 in zone i, declining exponentially with distance from the centre; baseline employment $e_{i}^{0}$ and potential earnings $w_{i}$ also declining with distance	Section 5.1.3
Childcare capacity	Ceiling $q_{j}^{max}\in \{90,120,150,180,210\}$ on the new places at each site; existing provision of 40–109 places at a quarter of the sites, which counts towards accessibility whether or not the site is selected and to which the new places are added (the full fixed cost is then incurred); 16,000 CNY per place per year plus a fixed cost that rises towards the centre	Equations (31) and (35); Section 5.1.3, Table 15
Accessibility	G2SFCA: places per child reachable from zone i, weighted by Gaussian distance decay	Equations (30)–(32)
Equity metric	Population-weighted CV of accessibility across zones (0 = equal access)	Equation (36)
Behavioural response	Logit maternal employment with slope $\beta_{A}=1.45$, i.e., 0.35 percentage points per point of coverage at the mean baseline	Equation (33)

CV, coefficient of variation; G2SFCA, Gaussian two-step floating-catchment area; CNY, Chinese yuan; all monetary values are annual.

**Table 16:** Objective values and mean ranks of MSCPO and the comparison algorithms on the four CFLC instances.

Instance	Metric	MSCPO	CPO	SBOA	BKA	RBMO	DBO	HHO	WOA	GWO	PSO
CFLC-1	Mean	**1.1080**	1.1082	1.1471	1.7231	1.2098	1.5180	1.7503	2.0021	1.2341	1.3382
	Std	0.0107	0.0112	0.1000	0.3532	0.1641	0.2618	0.3186	0.3306	0.1435	0.1856
	Best	1.1027	1.1027	1.1027	1.1278	1.1027	1.1053	1.2266	1.3600	1.1027	1.1053
CFLC-2	Mean	0.3738	**0.3685**	0.5209	1.1478	0.6010	0.7943	1.2398	1.3984	0.6304	0.8157
	Std	0.0301	0.0260	0.0842	0.2734	0.0886	0.1384	0.2352	0.2498	0.0952	0.1585
	Best	0.3406	0.3406	0.3642	0.7509	0.4505	0.5909	0.8003	0.9743	0.4448	0.6260
CFLC-3	Mean	**−1.7435**	−1.6602	−1.4412	−0.5487	−1.3961	−0.9694	−0.5742	−0.5047	−1.1013	−0.8552
	Std	0.0309	0.0490	0.1179	0.2156	0.1457	0.1642	0.2838	0.2728	0.1970	0.2558
	Best	−1.7860	−1.7721	−1.6290	−1.0349	−1.6120	−1.2610	−1.0779	−1.0623	−1.4709	−1.2141
CFLC-4	Mean	**−2.7985**	−2.5752	−2.2592	−1.1773	−2.3800	−1.6788	−1.4332	−1.1410	−1.6223	−1.5983
	Std	0.0566	0.0676	0.2128	0.2265	0.1537	0.2411	0.2484	0.3060	0.2060	0.2660
	Best	−2.9223	−2.6791	−2.6907	−1.6156	−2.6405	−2.1136	−1.8820	−1.5910	−2.0236	−2.1418
**Mean rank**		**1.25**	1.75	3.25	8.50	3.75	6.00	8.50	10.00	5.25	6.75

Friedman test over the four instances (n=4 blocks, k=10): $\chi_{F}^{2}(9)=35.07$, p<0.001; Iman–Davenport $F_{ID}(9,27)=113.47$, p<0.001. The best mean on each instance and the best mean rank are in bold. Post hoc tests with MSCPO as the control: Holm-adjusted p<0.01 against BKA, HHO and WOA and p≥0.06 against the other six. With CD=6.77 the Nemenyi test separates MSCPO from BKA, HHO and WOA only.

**Table 17:** Economic read-out of the best MSCPO and best CPO solutions on each childcare instance.

Instance	Algorithm	Centres	Places	Cost (M CNY)	Induced Mothers	Employment (pp)	Output/Subsidy	CV
CFLC-1	MSCPO	9	1230	26.7	426	4.13	1.20	0.410
	CPO	9	1230	26.7	426	4.13	1.20	0.410
CFLC-2	MSCPO	15	2520	53.4	858	4.15	1.25	0.413
	CPO	15	2520	53.4	858	4.15	1.25	0.413
CFLC-3	MSCPO	31	5160	108.9	1745	4.17	1.26	0.275
	CPO	31	5070	107.4	1714	4.09	1.26	0.255
CFLC-4	MSCPO	49	8160	172.5	2744	4.13	1.22	0.232
	CPO	49	8060	171.0	2715	4.09	1.22	0.250

Induced employment is the increase in the number of employed mothers implied by Equation (33); the output-to-subsidy ratio is V/C from Equations (34) and (35) and is not a social benefit–cost ratio (see the text); CV is the population-weighted coefficient of variation in accessibility from Equation (36); pp, percentage points; M CNY, million CNY.

**Table 18:** Sensitivity of the optimised childcare programme to the calibrated response slope (CFLC-4).

Target (pp/pp)	$\beta_{A}$	Centres	Places	Budget Used (%)	Induced Mothers	Employment (pp)	Output/Subsidy	CV of Access	Same Siting Decision as the Baseline (%)
0.20	0.84	7	1060	13	207	0.31	0.71	0.263	57
0.27	1.13	12	2280	27	597	0.90	1.00	0.187	62
0.35	1.46	50	8060	99	2735	4.12	1.23	0.215	100

The target is the marginal effect of local coverage on maternal employment, in percentage points per percentage point of coverage, at the mean baseline participation rate. Each row is a separate re-solution of CFLC-4 with the slope recalibrated to the target, so the 0.35 row differs slightly from the best solution reported in Table 17 ($\beta_{A}=1.45$). Recalibrating to the target on CFLC-4 gives $\beta_{A}=1.46$ for the 0.35 row, against the rounded value 1.45 used in the main runs. Each row reports the best of 12 independent MSCPO runs.

**Table 19:** Induced maternal employment and spatial equity under five siting rules and under the best MSCPO solution.

Instance	Siting Rule	Centres	Places	Budget Used (%)	Induced Mothers	Share of the Optimum (%)	Output/Subsidy	CV of Access
CFLC-1	Largest catchment first	9	1220	100	420	98.7	1.20	0.572
	Largest provision gap first	8	1200	96	409	96.2	1.19	0.711
	Cheapest site first	8	1230	95	399	93.7	1.07	1.621
	Highest earnings first	9	1200	100	411	96.6	1.22	0.613
	Uniform thin spread	20	600	97	209	49.0	0.58	1.142
	MSCPO optimum	9	1230	100	426	100.0	1.20	0.410
CFLC-2	Largest catchment first	16	2370	98	796	92.7	1.19	0.597
	Largest provision gap first	16	2400	99	809	94.3	1.18	0.636
	Cheapest site first	16	2550	99	848	98.9	1.22	0.974
	Highest earnings first	16	2370	98	795	92.6	1.19	0.581
	Uniform thin spread	39	1170	98	406	47.3	0.58	0.692
	MSCPO optimum	15	2520	99	858	100.0	1.25	0.413
CFLC-3	Largest catchment first	33	4980	100	1664	95.3	1.22	0.559
	Largest provision gap first	33	4980	100	1670	95.7	1.21	0.383
	Cheapest site first	33	5160	100	1762	100.9	1.26	0.706
	Highest earnings first	33	4970	100	1661	95.1	1.22	0.525
	Uniform thin spread	60	3600	100	1232	70.6	0.89	0.596
	MSCPO optimum	31	5160	100	1745	100.0	1.26	0.275
CFLC-4	Largest catchment first	54	7770	100	2561	93.3	1.17	0.451
	Largest provision gap first	54	7790	100	2577	93.9	1.17	0.358
	Cheapest site first	55	8040	99	2742	99.9	1.19	0.632
	Highest earnings first	54	7770	100	2559	93.3	1.17	0.495
	Uniform thin spread	100	5000	96	1710	62.3	0.77	0.490
	MSCPO optimum	49	8160	100	2744	100.0	1.22	0.232

The rules open sites in the stated order at their maximum viable capacity until the budget binds; the uniform rule instead spreads the budget over as many minimum-size centres as it will pay for. Share of the optimum is induced employment relative to the MSCPO solution on the same instance.

**Table 20:** The four FFSS instances.

Instance	E	D	D	Shifts to Cover	Contract Days per Worker	Carers ($\kappa_{e}\geq 0.5$)	Mean $\kappa_{e}$
FFSS-1	10	7	70	35	3–4	6 of 10	0.56
FFSS-2	16	14	224	136	8–9	12 of 16	0.59
FFSS-3	24	14	336	202	8–9	15 of 24	0.53
FFSS-4	30	21	630	396	13–14	11 of 30	0.40

E is the workforce, D the planning horizon in days and D=ED the search dimension. Calibration constants, identical across the four instances: burden weights $\theta_{1}=1.00$ (night), $\theta_{2}=0.55$ (weekend), $\theta_{3}=0.85$ (quick return), $\theta_{4}=0.35$ (instability), $\theta_{5}=0.45$ (preference mismatch); care amplification λ=1.60; minimum rest $\Delta^{min}=11$ h; objective weights $\omega_{1}=12.0$, $\omega_{2}=1.0$, $\omega_{3}=1.2$, $\omega_{4}=2.5$. Demand is split 40:36:24 across the morning, afternoon and night shifts.

**Table 21:** Objective values and mean ranks of MSCPO and the comparison algorithms on the four FFSS instances.

Instance	Metric	MSCPO	CPO	SBOA	BKA	RBMO	DBO	HHO	WOA	GWO	PSO
FFSS-1	Mean	1.1050	1.1008	1.1146	1.5269	**1.0945**	1.3180	1.3806	1.9378	1.1194	1.1896
	Std	0.0326	0.0326	0.0387	0.2314	0.0316	0.2424	0.2316	0.2550	0.0946	0.1815
	Best	1.0324	1.0325	1.0580	1.0888	1.0471	1.0712	1.0736	1.6364	1.0469	1.0508
FFSS-2	Mean	1.7220	1.7972	1.6825	2.5417	1.5328	1.7843	2.3928	2.9018	**1.3466**	1.4971
	Std	0.1619	0.1565	0.3285	0.2740	0.2342	0.2427	0.3557	0.3186	0.1842	0.2614
	Best	1.3417	1.4321	1.1819	1.9514	1.1939	1.2524	1.4744	2.0474	1.0982	1.1471
FFSS-3	Mean	2.1375	2.2243	1.8767	2.8571	1.8060	2.1680	2.7128	3.2295	**1.5177**	1.7759
	Std	0.1712	0.1622	0.2148	0.2964	0.2511	0.3225	0.3140	0.3593	0.1700	0.2720
	Best	1.8052	1.8339	1.5237	2.2308	1.4300	1.5451	2.0716	2.6105	1.2640	1.4649
FFSS-4	Mean	2.7503	2.8281	2.2914	3.7276	2.1079	2.6119	3.7060	4.4483	**1.7534**	2.0690
	Std	0.2699	0.2896	0.1969	0.3001	0.2015	0.3698	0.3806	0.4799	0.2719	0.2551
	Best	2.1893	2.2722	2.0269	3.1397	1.7528	1.9710	2.9988	3.2845	1.3680	1.5924
**Mean rank**		4.75	5.75	4.00	9.00	2.50	6.00	8.00	10.00	**2.00**	3.00

Friedman test over the four instances (n=4 blocks, k=10): $\chi_{F}^{2}(9)=30.27$, p<0.001; Iman–Davenport $F_{ID}(9,27)=15.86$, p<0.001. The best mean on each instance and the best mean rank are in bold. Post hoc tests with MSCPO as the control: no Holm-adjusted p is below 0.05 (the smallest is 0.13, against WOA), and with CD=6.77 the Nemenyi test separates MSCPO from none of the nine algorithms.

**Table 22:** Search behaviour of MSCPO and the comparison algorithms on FFSS-3.

Algorithm	Distinct Rosters	Share of Evaluations (%)	Mean Step	Cells Changed	Best $F_{FFSS}$
GWO	25,590	85.3	0.124	57.5	1.593
PSO	28,548	95.2	0.069	36.3	1.793
RBMO	17,009	56.7	0.125	52.1	1.837
SBOA	24,092	80.3	0.171	138.2	1.929
DBO	17,864	59.5	0.086	37.8	2.117
MSCPO	29,727	99.1	0.401	163.4	2.144
CPO	29,741	99.1	0.411	163.1	2.192
BKA	9988	33.3	0.161	59.5	2.640
HHO	6795	22.6	0.052	77.6	2.654
WOA	1391	4.6	0.078	15.6	3.065

Entries are means over five independent runs. The share of evaluations is the number of distinct rosters as a percentage of all function evaluations; rows are ordered by the objective reached.

**Table 23:** Roster quality of the best solution found on each scheduling instance by GWO, MSCPO and CPO.

Instance	Algorithm	Burden	Gini	Nights	Quick Returns	Violations	Burden, Carers	Burden, Others
FFSS-1	GWO	0.885	0.135	7	0	0.000	0.719	1.132
	MSCPO	0.843	0.158	7	0	0.000	0.729	1.014
	CPO	0.869	0.136	7	0	0.000	0.752	1.044
FFSS-2	GWO	1.023	0.063	28	3	0.000	1.013	1.054
	MSCPO	1.078	0.220	28	8	0.000	0.963	1.422
	CPO	1.182	0.208	28	11	0.000	1.127	1.347
FFSS-3	GWO	1.149	0.096	52	13	0.000	1.097	1.235
	MSCPO	1.082	0.255	52	9	0.167	1.104	1.046
	CPO	1.099	0.266	52	13	0.167	1.035	1.205
FFSS-4	GWO	1.109	0.077	99	41	0.067	1.117	1.105
	MSCPO	1.097	0.216	99	51	0.333	1.054	1.122
	CPO	1.051	0.219	99	45	0.383	0.986	1.088

The burden is the mean of $W_{e}$ over the workforce, Equation (41); the Gini coefficient is Equation (42); quick returns are rest intervals shorter than eleven hours; the last two columns give the mean burden of workers with $\kappa_{e}\geq 0.5$ and of the remainder. Violations are R(X) of Equation (43), per worker.

## Data Availability

The instance generators, the complete instance data for the eight application problems, the raw per-run results of every experiment reported in this paper and the analysis and plotting code are available from the corresponding author on request.

## Appendix A

The four tables below report the full error statistics behind the summary of Section 4.4: for every algorithm, every CEC2017 function and every dimension, the mean error over the thirty independent runs and its standard deviation. The best mean on each function is in bold.

Three regularities in these tables qualify the summary of Section 4.4. First, MSCPO has the lowest mean error on 19, 20, 22 and 24 functions at D=10, 30, 50 and 100, and where it is not the best its mean error is usually close to the best: at D=100 the five exceptions (F6, F8, F9, F10 and F19) lie between 1.7 and 12.6 per cent above the best mean, and at D=10 six of the ten exceptions are composition functions, on four of which (F21, F22, F25 and F27) the leading algorithms stall at almost the same local optimum and differ by less than 0.3 per cent. Among the largest relative gaps are F3 at D=10, where SBOA reaches the optimum to machine precision while MSCPO stops at an error of $6.4\times 10^{-8}$, F16 at D=30, where CPO’s mean error is less than half of MSCPO’s, F19 at D=50, where SBOA’s is about half, and F13 at D=30, where SBOA’s is about a third lower. Second, the size of the advantage depends strongly on the function group. The median ratio of CPO’s mean error to MSCPO’s is between 2.2 and 151 on the two unimodal functions, between 1.3 and 1.7 on the simple multimodal functions, between 1.4 and 1.8 on the hybrid functions and only between 1.01 and 1.20 on the composition functions, where the errors of the leading algorithms are compressed into a narrow band by the local optima of the composition. Third, MSCPO has the smallest standard deviation on functions 13, 15, 16 and 19 in the four dimensions, so its low mean errors are not bought with occasional failed runs; Section 4.8 quantifies this.

**Table A1:** Error statistics of MSCPO and the comparison algorithms on the CEC2017 suite (D=10).

Function	Metric	MSCPO	CPO	SBOA	BKA	RBMO	DBO	HHO	WOA	GWO	PSO
F1	Mean	**5.23 × 10^2^**	1.05 × 10^3^	1.73 × 10^3^	1.24 × 10^5^	3.30 × 10^3^	1.79 × 10^6^	6.95 × 10^9^	2.25 × 10^9^	6.83 × 10^7^	2.93 × 10^3^
	Std	7.33 × 10^2^	1.29 × 10^3^	2.05 × 10^3^	2.25 × 10^5^	3.40 × 10^3^	9.50 × 10^6^	3.48 × 10^9^	1.84 × 10^9^	1.90 × 10^8^	2.44 × 10^3^
F3	Mean	6.43 × 10^−8^	1.53 × 10^−7^	**2.27 × 10^−14^**	6.85 × 10^0^	2.45 × 10^−12^	2.01 × 10^−1^	1.50 × 10^4^	8.46 × 10^3^	3.26 × 10^2^	4.58 × 10^1^
	Std	9.54 × 10^−8^	2.30 × 10^−7^	1.25 × 10^−13^	2.02 × 10^1^	8.67 × 10^−12^	6.86 × 10^−1^	5.92 × 10^3^	4.03 × 10^3^	1.01 × 10^3^	1.74 × 10^2^
F4	Mean	**1.91 × 10^0^**	3.26 × 10^0^	5.03 × 10^0^	9.14 × 10^0^	5.84 × 10^0^	9.92 × 10^0^	2.34 × 10^2^	1.07 × 10^2^	2.55 × 10^1^	7.84 × 10^0^
	Std	2.02 × 10^0^	2.42 × 10^0^	1.92 × 10^0^	1.32 × 10^1^	2.77 × 10^0^	1.21 × 10^1^	1.38 × 10^2^	3.62 × 10^1^	3.50 × 10^1^	5.05 × 10^0^
F5	Mean	**5.54 × 10^0^**	1.45 × 10^1^	1.81 × 10^1^	2.13 × 10^2^	8.59 × 10^0^	4.57 × 10^1^	6.24 × 10^3^	2.61 × 10^3^	7.78 × 10^1^	1.80 × 10^2^
	Std	1.65 × 10^0^	3.51 × 10^0^	9.52 × 10^0^	2.22 × 10^2^	3.35 × 10^0^	3.02 × 10^1^	3.04 × 10^3^	2.11 × 10^3^	6.73 × 10^1^	3.87 × 10^2^
F6	Mean	**3.14 × 10^−9^**	7.45 × 10^−6^	6.00 × 10^−7^	6.36 × 10^−7^	6.87 × 10^−6^	1.72 × 10^−4^	8.75 × 10^−4^	9.47 × 10^−4^	5.67 × 10^−5^	4.30 × 10^−7^
	Std	1.06 × 10^−8^	9.73 × 10^−6^	1.46 × 10^−6^	1.98 × 10^−6^	2.91 × 10^−5^	3.51 × 10^−4^	1.56 × 10^−3^	1.27 × 10^−3^	1.69 × 10^−4^	1.07 × 10^−6^
F7	Mean	2.28 × 10^1^	2.91 × 10^1^	2.84 × 10^1^	2.77 × 10^2^	**2.03 × 10^1^**	6.08 × 10^1^	1.41 × 10^5^	5.00 × 10^4^	1.24 × 10^3^	2.84 × 10^3^
	Std	3.99 × 10^0^	3.51 × 10^0^	8.09 × 10^0^	1.02 × 10^3^	6.86 × 10^0^	2.37 × 10^1^	1.00 × 10^5^	4.16 × 10^4^	2.62 × 10^3^	6.38 × 10^3^
F8	Mean	1.66 × 10^1^	**1.60 × 10^1^**	2.23 × 10^1^	4.53 × 10^1^	2.35 × 10^1^	4.82 × 10^1^	8.56 × 10^1^	7.97 × 10^1^	3.14 × 10^1^	2.28 × 10^1^
	Std	5.39 × 10^0^	3.20 × 10^0^	7.84 × 10^0^	1.40 × 10^1^	8.88 × 10^0^	1.74 × 10^1^	2.07 × 10^1^	1.84 × 10^1^	1.05 × 10^1^	8.61 × 10^0^
F9	Mean	**1.10 × 10^−9^**	5.09 × 10^−9^	1.79 × 10^−2^	9.78 × 10^−1^	2.18 × 10^−2^	6.88 × 10^−1^	2.49 × 10^0^	2.52 × 10^0^	1.63 × 10^−1^	1.15 × 10^−1^
	Std	1.17 × 10^−9^	9.45 × 10^−9^	4.34 × 10^−2^	5.90 × 10^−1^	8.56 × 10^−2^	6.07 × 10^−1^	1.04 × 10^0^	1.22 × 10^0^	2.86 × 10^−1^	4.38 × 10^−1^
F10	Mean	**2.01 × 10^2^**	2.59 × 10^2^	3.51 × 10^2^	6.79 × 10^2^	4.26 × 10^2^	9.14 × 10^2^	1.35 × 10^3^	1.24 × 10^3^	4.96 × 10^2^	2.62 × 10^2^
	Std	1.56 × 10^2^	1.81 × 10^2^	2.49 × 10^2^	1.78 × 10^2^	2.41 × 10^2^	3.91 × 10^2^	3.46 × 10^2^	3.49 × 10^2^	3.98 × 10^2^	2.17 × 10^2^
F11	Mean	**7.30 × 10^0^**	1.37 × 10^1^	4.62 × 10^1^	1.07 × 10^3^	4.33 × 10^1^	9.45 × 10^2^	2.29 × 10^4^	2.80 × 10^4^	2.88 × 10^3^	4.69 × 10^2^
	Std	8.79 × 10^0^	8.29 × 10^0^	4.34 × 10^1^	1.41 × 10^3^	6.20 × 10^1^	1.68 × 10^3^	1.17 × 10^4^	3.74 × 10^4^	3.67 × 10^3^	2.33 × 10^3^
F12	Mean	**1.41 × 10^3^**	2.37 × 10^3^	5.45 × 10^3^	1.72 × 10^4^	2.23 × 10^4^	7.40 × 10^6^	1.59 × 10^8^	4.63 × 10^7^	2.18 × 10^6^	1.19 × 10^6^
	Std	1.67 × 10^3^	2.59 × 10^3^	5.79 × 10^3^	1.73 × 10^4^	1.99 × 10^4^	1.49 × 10^7^	3.37 × 10^8^	8.99 × 10^7^	2.28 × 10^6^	3.55 × 10^6^
F13	Mean	**3.02 × 10^1^**	2.54 × 10^2^	2.19 × 10^3^	1.03 × 10^4^	5.44 × 10^3^	3.91 × 10^5^	3.64 × 10^7^	1.16 × 10^6^	3.53 × 10^4^	1.31 × 10^5^
	Std	2.24 × 10^1^	1.10 × 10^3^	2.22 × 10^3^	5.03 × 10^3^	4.77 × 10^3^	1.12 × 10^6^	1.44 × 10^8^	1.55 × 10^6^	4.92 × 10^4^	1.90 × 10^5^
F14	Mean	**3.52 × 10^1^**	3.75 × 10^1^	1.71 × 10^3^	1.65 × 10^3^	8.74 × 10^2^	2.30 × 10^3^	1.78 × 10^4^	2.96 × 10^4^	6.35 × 10^3^	9.15 × 10^2^
	Std	1.40 × 10^1^	9.45 × 10^0^	1.72 × 10^3^	1.51 × 10^3^	1.65 × 10^3^	3.61 × 10^3^	3.48 × 10^3^	1.53 × 10^4^	3.79 × 10^3^	1.06 × 10^3^
F15	Mean	**1.08 × 10^1^**	2.68 × 10^1^	3.82 × 10^3^	1.01 × 10^4^	9.76 × 10^3^	4.77 × 10^4^	5.62 × 10^5^	5.23 × 10^5^	9.16 × 10^4^	1.39 × 10^5^
	Std	8.16 × 10^0^	2.22 × 10^1^	3.55 × 10^3^	6.43 × 10^3^	9.56 × 10^3^	1.08 × 10^5^	6.40 × 10^5^	6.66 × 10^5^	8.77 × 10^4^	4.85 × 10^5^
F16	Mean	1.13 × 10^0^	**9.78 × 10^−1^**	1.18 × 10^2^	7.63 × 10^2^	2.13 × 10^3^	9.91 × 10^2^	1.70 × 10^4^	1.25 × 10^4^	6.88 × 10^2^	6.46 × 10^2^
	Std	3.58 × 10^0^	9.22 × 10^−1^	1.62 × 10^2^	4.64 × 10^2^	5.16 × 10^3^	8.22 × 10^2^	1.85 × 10^4^	1.14 × 10^4^	4.59 × 10^2^	2.42 × 10^3^
F17	Mean	**2.55 × 10^1^**	3.65 × 10^1^	1.69 × 10^2^	3.62 × 10^2^	1.90 × 10^2^	4.96 × 10^2^	5.79 × 10^4^	4.68 × 10^4^	4.80 × 10^3^	1.49 × 10^3^
	Std	2.58 × 10^0^	3.82 × 10^1^	2.12 × 10^2^	2.58 × 10^2^	1.83 × 10^2^	4.11 × 10^2^	3.37 × 10^4^	3.93 × 10^4^	6.93 × 10^3^	6.65 × 10^3^
F18	Mean	**3.59 × 10^0^**	4.96 × 10^0^	2.29 × 10^3^	9.94 × 10^2^	2.16 × 10^3^	7.31 × 10^3^	1.94 × 10^4^	1.75 × 10^4^	9.42 × 10^3^	3.66 × 10^3^
	Std	6.90 × 10^0^	4.78 × 10^0^	2.52 × 10^3^	9.96 × 10^2^	2.98 × 10^3^	6.64 × 10^3^	7.76 × 10^3^	7.31 × 10^3^	5.94 × 10^3^	7.25 × 10^3^
F19	Mean	**2.53 × 10^0^**	3.08 × 10^0^	1.52 × 10^3^	2.51 × 10^3^	2.48 × 10^3^	1.93 × 10^4^	1.67 × 10^9^	9.11 × 10^7^	8.94 × 10^3^	7.44 × 10^3^
	Std	4.64 × 10^−1^	4.21 × 10^−1^	1.87 × 10^3^	3.89 × 10^3^	2.74 × 10^3^	2.23 × 10^4^	3.04 × 10^9^	4.99 × 10^8^	5.72 × 10^3^	9.24 × 10^3^
F20	Mean	**1.66 × 10^1^**	2.55 × 10^1^	2.73 × 10^1^	9.46 × 10^1^	5.59 × 10^1^	1.41 × 10^2^	6.08 × 10^2^	4.81 × 10^2^	6.51 × 10^1^	7.04 × 10^1^
	Std	7.82 × 10^0^	5.22 × 10^0^	5.39 × 10^0^	5.82 × 10^1^	5.42 × 10^1^	1.50 × 10^2^	5.13 × 10^2^	2.39 × 10^2^	4.26 × 10^1^	8.64 × 10^1^
F21	Mean	1.00 × 10^2^	1.04 × 10^2^	**1.00 × 10^2^**	1.88 × 10^2^	1.15 × 10^2^	1.32 × 10^2^	2.13 × 10^3^	1.55 × 10^3^	2.25 × 10^2^	4.21 × 10^2^
	Std	1.07 × 10^−3^	2.28 × 10^1^	5.33 × 10^−6^	1.13 × 10^2^	3.79 × 10^1^	6.55 × 10^1^	1.45 × 10^3^	1.37 × 10^3^	5.62 × 10^1^	2.32 × 10^2^
F22	Mean	1.00 × 10^2^	**1.00 × 10^2^**	1.02 × 10^2^	1.09 × 10^2^	1.03 × 10^2^	1.07 × 10^2^	1.70 × 10^2^	1.33 × 10^2^	1.06 × 10^2^	1.08 × 10^2^
	Std	6.63 × 10^−1^	5.27 × 10^−1^	1.23 × 10^0^	4.70 × 10^0^	1.13 × 10^0^	4.24 × 10^0^	6.33 × 10^1^	1.28 × 10^1^	3.62 × 10^0^	5.21 × 10^0^
F23	Mean	**1.03 × 10^2^**	1.17 × 10^2^	1.13 × 10^2^	1.65 × 10^2^	1.40 × 10^2^	1.27 × 10^2^	1.93 × 10^3^	1.07 × 10^3^	2.21 × 10^2^	3.51 × 10^2^
	Std	1.83 × 10^1^	3.79 × 10^1^	3.46 × 10^1^	5.02 × 10^1^	4.98 × 10^1^	4.52 × 10^1^	9.13 × 10^2^	3.19 × 10^2^	7.26 × 10^1^	3.58 × 10^2^
F24	Mean	1.87 × 10^2^	2.04 × 10^2^	1.98 × 10^2^	1.91 × 10^2^	2.03 × 10^2^	**1.82 × 10^2^**	2.43 × 10^3^	1.66 × 10^3^	3.95 × 10^2^	1.02 × 10^3^
	Std	3.46 × 10^1^	2.02 × 10^1^	4.46 × 10^1^	4.48 × 10^1^	6.84 × 10^1^	5.56 × 10^1^	8.44 × 10^2^	4.65 × 10^2^	1.81 × 10^2^	7.73 × 10^2^
F25	Mean	4.81 × 10^2^	**4.80 × 10^2^**	4.91 × 10^2^	5.14 × 10^2^	4.90 × 10^2^	5.07 × 10^2^	7.80 × 10^2^	6.92 × 10^2^	5.01 × 10^2^	4.85 × 10^2^
	Std	8.91 × 10^0^	5.28 × 10^0^	1.40 × 10^1^	3.60 × 10^1^	1.86 × 10^1^	3.34 × 10^1^	1.47 × 10^2^	9.66 × 10^1^	2.45 × 10^1^	1.04 × 10^1^
F26	Mean	4.32 × 10^2^	4.29 × 10^2^	4.03 × 10^2^	4.60 × 10^2^	4.72 × 10^2^	**3.37 × 10^2^**	5.89 × 10^2^	5.29 × 10^2^	4.70 × 10^2^	4.88 × 10^2^
	Std	1.17 × 10^2^	1.17 × 10^2^	1.61 × 10^2^	8.78 × 10^1^	1.67 × 10^1^	2.23 × 10^2^	7.83 × 10^1^	1.53 × 10^2^	8.35 × 10^0^	3.87 × 10^1^
F27	Mean	3.91 × 10^2^	**3.91 × 10^2^**	3.94 × 10^2^	4.08 × 10^2^	3.96 × 10^2^	4.14 × 10^2^	5.52 × 10^2^	4.79 × 10^2^	4.00 × 10^2^	4.16 × 10^2^
	Std	2.31 × 10^0^	1.82 × 10^0^	3.16 × 10^0^	1.97 × 10^1^	3.81 × 10^0^	2.45 × 10^1^	1.10 × 10^2^	7.43 × 10^1^	1.82 × 10^1^	1.97 × 10^1^
F28	Mean	**2.03 × 10^−3^**	2.09 × 10^−3^	2.39 × 10^0^	8.87 × 10^1^	7.99 × 10^1^	1.90 × 10^2^	5.90 × 10^2^	4.90 × 10^2^	1.54 × 10^2^	3.16 × 10^2^
	Std	1.50 × 10^−3^	2.36 × 10^−3^	6.38 × 10^0^	7.79 × 10^1^	1.71 × 10^2^	1.95 × 10^2^	1.74 × 10^2^	1.30 × 10^2^	1.40 × 10^2^	1.40 × 10^2^
F29	Mean	**1.03 × 10^3^**	1.04 × 10^3^	1.24 × 10^3^	3.96 × 10^3^	3.25 × 10^3^	1.25 × 10^4^	5.03 × 10^7^	2.75 × 10^7^	1.46 × 10^4^	1.15 × 10^4^
	Std	3.92 × 10^1^	4.91 × 10^1^	1.46 × 10^2^	7.03 × 10^3^	5.97 × 10^3^	1.41 × 10^4^	6.94 × 10^7^	8.45 × 10^7^	1.20 × 10^4^	2.40 × 10^4^
F30	Mean	**5.04 × 10^3^**	5.11 × 10^3^	1.21 × 10^4^	1.81 × 10^4^	1.76 × 10^4^	3.18 × 10^6^	9.98 × 10^8^	3.18 × 10^8^	3.21 × 10^4^	1.76 × 10^4^
	Std	2.47 × 10^3^	2.98 × 10^3^	3.90 × 10^3^	1.30 × 10^4^	7.71 × 10^3^	1.30 × 10^7^	2.02 × 10^9^	7.57 × 10^8^	3.29 × 10^4^	8.06 × 10^3^

Values are given to three significant figures in scientific notation. The lowest mean error on each function is in bold.

**Table A2:** Error statistics of MSCPO and the comparison algorithms on the CEC2017 suite (D=30).

Function	Metric	MSCPO	CPO	SBOA	BKA	RBMO	DBO	HHO	WOA	GWO	PSO
F1	Mean	**3.66 × 10^3^**	4.21 × 10^3^	2.00 × 10^4^	7.23 × 10^9^	5.19 × 10^4^	5.58 × 10^7^	4.53 × 10^10^	3.73 × 10^10^	1.99 × 10^9^	2.65 × 10^9^
	Std	3.92 × 10^3^	3.16 × 10^3^	1.84 × 10^4^	4.37 × 10^9^	4.42 × 10^4^	4.97 × 10^7^	8.95 × 10^9^	8.61 × 10^9^	1.40 × 10^9^	4.57 × 10^9^
F3	Mean	**3.50 × 10^−3^**	1.05 × 10^0^	1.14 × 10^1^	1.59 × 10^4^	4.40 × 10^2^	2.06 × 10^3^	9.41 × 10^4^	8.65 × 10^4^	1.59 × 10^4^	2.52 × 10^3^
	Std	1.75 × 10^−3^	2.08 × 10^0^	1.36 × 10^1^	7.04 × 10^3^	5.10 × 10^2^	1.37 × 10^3^	2.05 × 10^4^	1.43 × 10^4^	7.66 × 10^3^	2.90 × 10^3^
F4	Mean	4.31 × 10^1^	**3.84 × 10^1^**	8.94 × 10^1^	6.34 × 10^2^	5.25 × 10^1^	2.07 × 10^2^	1.05 × 10^4^	7.52 × 10^3^	2.38 × 10^2^	2.97 × 10^2^
	Std	2.09 × 10^1^	1.82 × 10^1^	4.37 × 10^1^	4.03 × 10^2^	2.97 × 10^1^	1.28 × 10^2^	3.94 × 10^3^	2.34 × 10^3^	1.41 × 10^2^	4.00 × 10^2^
F5	Mean	**1.24 × 10^2^**	1.85 × 10^2^	1.61 × 10^2^	1.48 × 10^4^	1.40 × 10^2^	6.73 × 10^2^	6.52 × 10^4^	4.95 × 10^4^	3.10 × 10^3^	4.07 × 10^3^
	Std	3.55 × 10^1^	2.38 × 10^1^	6.13 × 10^1^	5.29 × 10^3^	2.32 × 10^2^	2.54 × 10^2^	9.50 × 10^3^	1.26 × 10^4^	2.43 × 10^3^	2.72 × 10^3^
F6	Mean	**7.79 × 10^−5^**	2.83 × 10^−3^	2.90 × 10^−4^	1.08 × 10^−3^	4.03 × 10^−4^	2.67 × 10^−3^	2.21 × 10^−2^	2.79 × 10^−2^	5.81 × 10^−4^	2.83 × 10^−4^
	Std	9.68 × 10^−5^	1.99 × 10^−3^	2.76 × 10^−4^	9.82 × 10^−4^	3.86 × 10^−4^	3.83 × 10^−3^	8.74 × 10^−3^	1.23 × 10^−2^	8.77 × 10^−4^	7.87 × 10^−4^
F7	Mean	2.22 × 10^2^	2.47 × 10^2^	2.82 × 10^2^	3.80 × 10^5^	**1.68 × 10^2^**	6.03 × 10^3^	2.25 × 10^6^	1.69 × 10^6^	1.21 × 10^5^	1.70 × 10^5^
	Std	1.69 × 10^1^	2.10 × 10^1^	8.37 × 10^1^	1.66 × 10^5^	3.47 × 10^1^	3.88 × 10^3^	4.57 × 10^5^	4.71 × 10^5^	1.06 × 10^5^	1.62 × 10^5^
F8	Mean	**1.56 × 10^2^**	2.37 × 10^2^	2.07 × 10^2^	4.21 × 10^2^	2.15 × 10^2^	4.62 × 10^2^	6.49 × 10^2^	6.44 × 10^2^	2.27 × 10^2^	2.05 × 10^2^
	Std	4.43 × 10^1^	4.41 × 10^1^	4.33 × 10^1^	5.97 × 10^1^	6.81 × 10^1^	7.81 × 10^1^	7.56 × 10^1^	7.18 × 10^1^	8.95 × 10^1^	5.58 × 10^1^
F9	Mean	5.57 × 10^−1^	**4.47 × 10^−1^**	3.46 × 10^0^	1.12 × 10^1^	2.36 × 10^0^	1.42 × 10^1^	2.82 × 10^1^	2.79 × 10^1^	4.14 × 10^0^	4.75 × 10^0^
	Std	5.92 × 10^−1^	4.32 × 10^−1^	1.20 × 10^0^	3.91 × 10^0^	1.76 × 10^0^	5.25 × 10^0^	5.70 × 10^0^	6.61 × 10^0^	2.17 × 10^0^	3.97 × 10^0^
F10	Mean	**2.78 × 10^3^**	4.49 × 10^3^	3.23 × 10^3^	3.99 × 10^3^	4.09 × 10^3^	4.43 × 10^3^	6.55 × 10^3^	6.82 × 10^3^	4.06 × 10^3^	3.19 × 10^3^
	Std	6.54 × 10^2^	9.03 × 10^2^	8.47 × 10^2^	5.42 × 10^2^	6.66 × 10^2^	7.63 × 10^2^	5.29 × 10^2^	7.81 × 10^2^	1.87 × 10^3^	6.03 × 10^2^
F11	Mean	**3.21 × 10^3^**	8.36 × 10^3^	1.52 × 10^4^	6.87 × 10^4^	3.92 × 10^4^	2.29 × 10^5^	5.18 × 10^7^	3.38 × 10^6^	1.40 × 10^5^	8.53 × 10^4^
	Std	3.11 × 10^3^	6.64 × 10^3^	1.17 × 10^4^	2.57 × 10^4^	2.49 × 10^4^	9.03 × 10^4^	9.62 × 10^7^	8.02 × 10^6^	8.89 × 10^4^	5.47 × 10^4^
F12	Mean	**6.69 × 10^4^**	1.68 × 10^5^	6.85 × 10^4^	1.46 × 10^7^	3.89 × 10^5^	1.65 × 10^7^	8.27 × 10^9^	3.08 × 10^9^	5.34 × 10^7^	4.82 × 10^7^
	Std	6.11 × 10^4^	1.69 × 10^5^	6.81 × 10^4^	1.24 × 10^7^	4.40 × 10^5^	1.97 × 10^7^	4.67 × 10^9^	1.85 × 10^9^	1.25 × 10^8^	1.05 × 10^8^
F13	Mean	7.95 × 10^3^	1.96 × 10^4^	**5.39 × 10^3^**	2.28 × 10^7^	2.80 × 10^4^	3.29 × 10^6^	1.01 × 10^10^	3.45 × 10^9^	3.33 × 10^7^	4.14 × 10^7^
	Std	7.50 × 10^3^	2.33 × 10^4^	5.74 × 10^3^	3.27 × 10^7^	2.12 × 10^4^	5.85 × 10^6^	5.07 × 10^9^	2.12 × 10^9^	8.67 × 10^7^	6.48 × 10^7^
F14	Mean	**2.04 × 10^4^**	2.87 × 10^4^	1.04 × 10^5^	2.02 × 10^5^	3.01 × 10^5^	6.75 × 10^5^	9.31 × 10^6^	8.57 × 10^6^	1.35 × 10^6^	1.87 × 10^5^
	Std	1.65 × 10^4^	2.27 × 10^4^	8.29 × 10^4^	9.21 × 10^4^	1.63 × 10^5^	6.59 × 10^5^	7.45 × 10^6^	9.60 × 10^6^	1.75 × 10^6^	1.40 × 10^5^
F15	Mean	**1.72 × 10^4^**	2.38 × 10^4^	1.80 × 10^4^	1.16 × 10^6^	4.06 × 10^4^	3.09 × 10^6^	3.60 × 10^9^	1.28 × 10^9^	5.67 × 10^6^	1.05 × 10^7^
	Std	7.25 × 10^3^	1.10 × 10^4^	1.24 × 10^4^	1.95 × 10^6^	2.11 × 10^4^	1.04 × 10^7^	1.91 × 10^9^	9.64 × 10^8^	1.89 × 10^7^	2.20 × 10^7^
F16	Mean	7.71 × 10^1^	**3.41 × 10^1^**	3.93 × 10^2^	2.05 × 10^5^	2.76 × 10^3^	1.41 × 10^6^	4.59 × 10^8^	2.70 × 10^8^	2.22 × 10^6^	2.51 × 10^5^
	Std	9.76 × 10^1^	6.02 × 10^1^	7.21 × 10^2^	6.55 × 10^5^	7.52 × 10^3^	1.88 × 10^6^	7.20 × 10^8^	5.04 × 10^8^	4.14 × 10^6^	1.14 × 10^6^
F17	Mean	**7.04 × 10^2^**	1.68 × 10^3^	1.06 × 10^3^	1.38 × 10^4^	1.92 × 10^3^	1.31 × 10^5^	2.99 × 10^13^	6.42 × 10^10^	4.96 × 10^4^	6.32 × 10^4^
	Std	3.16 × 10^2^	8.57 × 10^2^	4.14 × 10^2^	7.65 × 10^3^	1.69 × 10^3^	2.41 × 10^5^	6.67 × 10^13^	2.34 × 10^11^	1.97 × 10^4^	7.51 × 10^4^
F18	Mean	**2.11 × 10^4^**	2.17 × 10^4^	5.80 × 10^4^	8.39 × 10^4^	1.82 × 10^5^	2.48 × 10^5^	4.39 × 10^5^	5.53 × 10^5^	5.57 × 10^5^	1.16 × 10^5^
	Std	1.03 × 10^4^	7.93 × 10^3^	3.42 × 10^4^	3.44 × 10^4^	1.87 × 10^5^	3.20 × 10^5^	5.15 × 10^5^	6.17 × 10^5^	1.30 × 10^6^	6.18 × 10^4^
F19	Mean	1.21 × 10^4^	**8.77 × 10^3^**	1.23 × 10^4^	1.12 × 10^7^	3.19 × 10^4^	8.71 × 10^6^	2.46 × 10^14^	4.04 × 10^13^	4.48 × 10^9^	3.77 × 10^9^
	Std	1.19 × 10^4^	7.45 × 10^3^	1.17 × 10^4^	5.44 × 10^7^	2.57 × 10^4^	2.34 × 10^7^	8.24 × 10^14^	1.04 × 10^14^	7.60 × 10^9^	9.01 × 10^9^
F20	Mean	**1.82 × 10^2^**	2.49 × 10^2^	3.12 × 10^2^	2.48 × 10^3^	4.18 × 10^2^	1.50 × 10^3^	9.48 × 10^3^	8.56 × 10^3^	9.37 × 10^2^	5.30 × 10^2^
	Std	1.38 × 10^2^	1.44 × 10^2^	1.45 × 10^2^	7.40 × 10^2^	2.14 × 10^2^	8.24 × 10^2^	3.66 × 10^3^	3.56 × 10^3^	4.65 × 10^2^	2.99 × 10^2^
F21	Mean	**1.53 × 10^2^**	2.70 × 10^2^	1.88 × 10^2^	5.05 × 10^3^	3.35 × 10^2^	7.03 × 10^2^	4.40 × 10^4^	2.93 × 10^4^	2.59 × 10^3^	2.52 × 10^3^
	Std	9.97 × 10^1^	1.51 × 10^2^	1.29 × 10^2^	3.89 × 10^3^	2.23 × 10^2^	2.96 × 10^2^	9.79 × 10^3^	9.59 × 10^3^	2.39 × 10^3^	1.78 × 10^3^
F22	Mean	1.71 × 10^2^	**1.70 × 10^2^**	1.85 × 10^2^	9.71 × 10^2^	1.83 × 10^2^	2.33 × 10^2^	2.62 × 10^3^	2.30 × 10^3^	2.06 × 10^2^	2.78 × 10^2^
	Std	5.92 × 10^0^	4.02 × 10^0^	1.15 × 10^1^	7.80 × 10^2^	1.27 × 10^1^	3.06 × 10^1^	1.17 × 10^3^	9.66 × 10^2^	1.57 × 10^1^	6.71 × 10^1^
F23	Mean	**2.09 × 10^2^**	2.54 × 10^2^	2.54 × 10^2^	1.32 × 10^4^	5.53 × 10^2^	1.28 × 10^3^	5.77 × 10^4^	4.19 × 10^4^	5.16 × 10^3^	8.33 × 10^3^
	Std	4.24 × 10^1^	1.17 × 10^2^	1.06 × 10^2^	5.44 × 10^3^	9.25 × 10^2^	2.69 × 10^2^	7.74 × 10^3^	1.14 × 10^4^	2.68 × 10^3^	4.88 × 10^3^
F24	Mean	2.07 × 10^2^	**2.02 × 10^2^**	2.25 × 10^2^	1.00 × 10^4^	2.50 × 10^2^	8.44 × 10^2^	3.07 × 10^4^	2.45 × 10^4^	2.84 × 10^3^	6.39 × 10^3^
	Std	3.58 × 10^1^	3.11 × 10^−1^	6.39 × 10^1^	5.24 × 10^3^	7.56 × 10^1^	2.64 × 10^2^	3.47 × 10^3^	5.21 × 10^3^	1.77 × 10^3^	2.74 × 10^3^
F25	Mean	**4.23 × 10^2^**	4.24 × 10^2^	4.33 × 10^2^	7.35 × 10^2^	4.24 × 10^2^	5.10 × 10^2^	3.65 × 10^3^	2.67 × 10^3^	5.73 × 10^2^	5.19 × 10^2^
	Std	4.20 × 10^0^	4.24 × 10^0^	1.47 × 10^1^	1.60 × 10^2^	5.19 × 10^0^	5.28 × 10^1^	9.41 × 10^2^	7.58 × 10^2^	8.09 × 10^1^	1.21 × 10^2^
F26	Mean	**8.30 × 10^2^**	8.33 × 10^2^	8.41 × 10^2^	9.55 × 10^2^	8.72 × 10^2^	1.15 × 10^3^	5.45 × 10^3^	4.00 × 10^3^	9.40 × 10^2^	1.07 × 10^3^
	Std	3.71 × 10^0^	6.57 × 10^0^	2.32 × 10^1^	9.55 × 10^1^	1.51 × 10^1^	3.68 × 10^2^	3.25 × 10^3^	3.50 × 10^3^	7.99 × 10^1^	2.14 × 10^2^
F27	Mean	5.16 × 10^2^	**5.13 × 10^2^**	5.36 × 10^2^	7.62 × 10^2^	5.33 × 10^2^	6.74 × 10^2^	1.57 × 10^3^	1.12 × 10^3^	5.77 × 10^2^	6.01 × 10^2^
	Std	1.54 × 10^1^	9.74 × 10^0^	1.60 × 10^1^	1.35 × 10^2^	1.89 × 10^1^	7.91 × 10^1^	4.94 × 10^2^	2.51 × 10^2^	4.30 × 10^1^	6.85 × 10^1^
F28	Mean	**1.08 × 10^1^**	3.27 × 10^1^	1.16 × 10^2^	5.64 × 10^2^	4.17 × 10^2^	5.39 × 10^2^	3.34 × 10^3^	1.85 × 10^3^	4.95 × 10^2^	7.28 × 10^2^
	Std	4.40 × 10^1^	8.54 × 10^1^	1.70 × 10^2^	7.52 × 10^1^	1.16 × 10^2^	8.97 × 10^1^	1.01 × 10^3^	5.07 × 10^2^	3.60 × 10^1^	2.16 × 10^2^
F29	Mean	**5.11 × 10^3^**	1.17 × 10^4^	1.68 × 10^4^	1.21 × 10^7^	1.64 × 10^5^	2.00 × 10^8^	8.57 × 10^11^	6.57 × 10^10^	1.48 × 10^8^	1.19 × 10^8^
	Std	1.90 × 10^3^	8.19 × 10^3^	1.42 × 10^4^	2.03 × 10^7^	3.54 × 10^5^	3.55 × 10^8^	2.79 × 10^12^	2.91 × 10^11^	1.10 × 10^8^	4.94 × 10^8^
F30	Mean	**3.71 × 10^4^**	5.85 × 10^4^	9.50 × 10^4^	3.59 × 10^7^	3.95 × 10^5^	1.34 × 10^8^	3.11 × 10^11^	6.80 × 10^10^	2.63 × 10^8^	1.13 × 10^7^
	Std	2.43 × 10^4^	4.08 × 10^4^	3.36 × 10^5^	8.97 × 10^7^	9.27 × 10^5^	4.51 × 10^8^	5.43 × 10^11^	1.72 × 10^11^	3.50 × 10^8^	3.79 × 10^7^

Values are given to three significant figures in scientific notation. The lowest mean error on each function is in bold.

**Table A3:** Error statistics of MSCPO and the comparison algorithms on the CEC2017 suite (D=50).

Function	Metric	MSCPO	CPO	SBOA	BKA	RBMO	DBO	HHO	WOA	GWO	PSO
F1	Mean	**1.68 × 10^4^**	2.06 × 10^5^	3.21 × 10^7^	3.52 × 10^10^	4.60 × 10^8^	2.51 × 10^9^	1.02 × 10^11^	8.59 × 10^10^	1.02 × 10^10^	6.79 × 10^9^
	Std	5.85 × 10^3^	8.95 × 10^4^	9.33 × 10^7^	8.57 × 10^9^	8.35 × 10^8^	8.28 × 10^9^	7.20 × 10^9^	9.95 × 10^9^	4.92 × 10^9^	4.43 × 10^9^
F3	Mean	**3.74 × 10^1^**	1.17 × 10^3^	1.28 × 10^3^	5.09 × 10^4^	1.19 × 10^4^	2.06 × 10^4^	1.47 × 10^5^	1.51 × 10^5^	3.37 × 10^4^	7.64 × 10^3^
	Std	4.40 × 10^1^	7.11 × 10^2^	1.03 × 10^3^	1.09 × 10^4^	4.10 × 10^3^	2.15 × 10^4^	9.13 × 10^3^	1.02 × 10^4^	9.56 × 10^3^	8.87 × 10^3^
F4	Mean	**1.57 × 10^2^**	1.96 × 10^2^	2.30 × 10^2^	5.33 × 10^3^	2.30 × 10^2^	4.35 × 10^2^	3.58 × 10^4^	2.95 × 10^4^	1.06 × 10^3^	1.01 × 10^3^
	Std	5.58 × 10^1^	3.96 × 10^1^	5.30 × 10^1^	1.91 × 10^3^	6.20 × 10^1^	1.02 × 10^2^	5.93 × 10^3^	6.90 × 10^3^	6.61 × 10^2^	7.62 × 10^2^
F5	Mean	**3.77 × 10^2^**	4.88 × 10^2^	5.14 × 10^2^	4.55 × 10^4^	7.04 × 10^2^	2.09 × 10^3^	1.18 × 10^5^	9.72 × 10^4^	1.15 × 10^4^	1.11 × 10^4^
	Std	5.58 × 10^1^	3.76 × 10^1^	1.09 × 10^2^	9.31 × 10^3^	5.42 × 10^2^	9.93 × 10^2^	6.00 × 10^3^	1.33 × 10^4^	5.09 × 10^3^	4.94 × 10^3^
F6	Mean	**3.79 × 10^−4^**	7.59 × 10^−3^	5.33 × 10^−4^	1.83 × 10^−3^	2.22 × 10^−3^	2.46 × 10^−3^	3.32 × 10^−2^	4.19 × 10^−2^	1.94 × 10^−3^	6.93 × 10^−4^
	Std	4.40 × 10^−4^	3.52 × 10^−3^	4.41 × 10^−4^	1.26 × 10^−3^	1.47 × 10^−3^	2.24 × 10^−3^	7.25 × 10^−3^	1.41 × 10^−2^	4.88 × 10^−3^	7.52 × 10^−4^
F7	Mean	**5.62 × 10^2^**	6.13 × 10^2^	1.33 × 10^3^	1.76 × 10^6^	1.17 × 10^4^	7.04 × 10^4^	5.52 × 10^6^	4.57 × 10^6^	4.36 × 10^5^	5.86 × 10^5^
	Std	5.09 × 10^1^	5.26 × 10^1^	5.50 × 10^2^	4.44 × 10^5^	2.03 × 10^4^	2.60 × 10^4^	5.56 × 10^5^	7.02 × 10^5^	2.10 × 10^5^	3.13 × 10^5^
F8	Mean	**3.48 × 10^2^**	6.26 × 10^2^	4.99 × 10^2^	8.90 × 10^2^	5.47 × 10^2^	8.88 × 10^2^	1.32 × 10^3^	1.29 × 10^3^	5.03 × 10^2^	5.68 × 10^2^
	Std	7.48 × 10^1^	6.31 × 10^1^	1.02 × 10^2^	1.15 × 10^2^	7.81 × 10^1^	1.62 × 10^2^	1.09 × 10^2^	9.73 × 10^1^	1.94 × 10^2^	1.53 × 10^2^
F9	Mean	5.11 × 10^0^	**3.94 × 10^0^**	1.37 × 10^1^	2.80 × 10^1^	1.05 × 10^1^	4.26 × 10^1^	7.91 × 10^1^	7.65 × 10^1^	1.24 × 10^1^	1.45 × 10^1^
	Std	2.64 × 10^0^	1.66 × 10^0^	3.18 × 10^0^	6.98 × 10^0^	5.34 × 10^0^	1.68 × 10^1^	1.50 × 10^1^	1.90 × 10^1^	4.46 × 10^0^	5.86 × 10^0^
F10	Mean	**6.47 × 10^3^**	1.19 × 10^4^	7.03 × 10^3^	8.28 × 10^3^	9.47 × 10^3^	9.54 × 10^3^	1.37 × 10^4^	1.40 × 10^4^	9.92 × 10^3^	7.28 × 10^3^
	Std	1.11 × 10^3^	6.49 × 10^2^	9.00 × 10^2^	9.20 × 10^2^	1.28 × 10^3^	2.36 × 10^3^	7.63 × 10^2^	8.04 × 10^2^	3.55 × 10^3^	1.83 × 10^3^
F11	Mean	**4.30 × 10^3^**	8.83 × 10^3^	2.05 × 10^4^	1.53 × 10^5^	1.05 × 10^5^	3.81 × 10^5^	6.63 × 10^8^	4.32 × 10^8^	1.27 × 10^6^	4.12 × 10^6^
	Std	2.68 × 10^3^	4.98 × 10^3^	8.59 × 10^3^	6.62 × 10^4^	4.37 × 10^4^	1.18 × 10^5^	6.73 × 10^8^	4.71 × 10^8^	4.88 × 10^6^	1.35 × 10^7^
F12	Mean	**3.45 × 10^5^**	7.53 × 10^5^	8.18 × 10^5^	1.31 × 10^9^	3.73 × 10^6^	1.27 × 10^8^	1.60 × 10^10^	1.08 × 10^10^	4.08 × 10^8^	3.92 × 10^8^
	Std	2.31 × 10^5^	6.47 × 10^5^	5.53 × 10^5^	1.00 × 10^9^	3.50 × 10^6^	1.15 × 10^8^	4.15 × 10^9^	3.67 × 10^9^	5.53 × 10^8^	6.39 × 10^8^
F13	Mean	**9.41 × 10^4^**	9.96 × 10^4^	1.02 × 10^5^	5.62 × 10^9^	4.06 × 10^5^	8.86 × 10^7^	6.39 × 10^10^	4.17 × 10^10^	9.83 × 10^8^	1.79 × 10^9^
	Std	3.30 × 10^4^	2.94 × 10^4^	5.86 × 10^4^	5.53 × 10^9^	1.74 × 10^5^	9.61 × 10^7^	1.44 × 10^10^	1.48 × 10^10^	1.35 × 10^9^	2.73 × 10^9^
F14	Mean	**1.96 × 10^5^**	2.91 × 10^5^	5.34 × 10^5^	7.31 × 10^5^	1.32 × 10^6^	6.40 × 10^6^	7.10 × 10^7^	3.05 × 10^7^	1.59 × 10^6^	1.28 × 10^6^
	Std	1.42 × 10^5^	1.53 × 10^5^	3.63 × 10^5^	4.17 × 10^5^	8.27 × 10^5^	7.17 × 10^6^	7.71 × 10^7^	2.02 × 10^7^	1.14 × 10^6^	8.83 × 10^5^
F15	Mean	**3.67 × 10^4^**	4.94 × 10^4^	3.95 × 10^4^	1.20 × 10^9^	1.13 × 10^5^	1.10 × 10^7^	2.33 × 10^10^	1.10 × 10^10^	1.84 × 10^8^	3.81 × 10^8^
	Std	9.83 × 10^3^	1.83 × 10^4^	1.92 × 10^4^	1.93 × 10^9^	4.16 × 10^4^	2.34 × 10^7^	6.86 × 10^9^	5.20 × 10^9^	3.41 × 10^8^	8.13 × 10^8^
F16	Mean	1.38 × 10^3^	**1.37 × 10^3^**	2.80 × 10^3^	2.74 × 10^7^	1.43 × 10^4^	8.32 × 10^5^	1.77 × 10^10^	4.31 × 10^9^	6.47 × 10^6^	1.12 × 10^8^
	Std	1.59 × 10^3^	1.48 × 10^3^	3.55 × 10^3^	9.11 × 10^7^	5.29 × 10^4^	7.49 × 10^5^	1.35 × 10^10^	5.26 × 10^9^	1.66 × 10^7^	2.95 × 10^8^
F17	Mean	**2.19 × 10^3^**	6.36 × 10^3^	2.66 × 10^3^	2.77 × 10^4^	1.27 × 10^4^	1.39 × 10^5^	4.24 × 10^12^	4.33 × 10^12^	4.63 × 10^4^	3.90 × 10^4^
	Std	5.88 × 10^2^	5.39 × 10^3^	1.12 × 10^3^	7.00 × 10^3^	1.62 × 10^4^	1.39 × 10^5^	4.66 × 10^12^	1.26 × 10^13^	1.13 × 10^4^	2.86 × 10^4^
F18	Mean	**1.54 × 10^5^**	2.08 × 10^5^	3.16 × 10^5^	3.50 × 10^5^	8.98 × 10^5^	2.02 × 10^6^	7.73 × 10^6^	5.89 × 10^6^	7.05 × 10^5^	8.10 × 10^5^
	Std	3.95 × 10^4^	1.10 × 10^5^	1.22 × 10^5^	1.01 × 10^5^	3.39 × 10^5^	2.78 × 10^6^	1.18 × 10^7^	5.02 × 10^6^	2.32 × 10^5^	6.49 × 10^5^
F19	Mean	7.92 × 10^3^	1.27 × 10^4^	**4.11 × 10^3^**	7.55 × 10^10^	1.23 × 10^5^	5.81 × 10^9^	9.77 × 10^14^	4.47 × 10^14^	1.90 × 10^10^	4.98 × 10^10^
	Std	8.18 × 10^3^	9.44 × 10^3^	3.08 × 10^3^	3.58 × 10^11^	3.68 × 10^5^	1.92 × 10^10^	1.42 × 10^15^	1.06 × 10^15^	1.83 × 10^10^	6.82 × 10^10^
F20	Mean	2.51 × 10^2^	**2.49 × 10^2^**	3.85 × 10^2^	5.52 × 10^3^	8.34 × 10^2^	2.40 × 10^3^	9.66 × 10^3^	9.51 × 10^3^	1.53 × 10^3^	1.09 × 10^3^
	Std	2.28 × 10^2^	1.39 × 10^2^	3.00 × 10^2^	1.53 × 10^3^	5.04 × 10^2^	1.06 × 10^3^	1.36 × 10^3^	2.09 × 10^3^	7.82 × 10^2^	8.39 × 10^2^
F21	Mean	**2.91 × 10^2^**	5.03 × 10^2^	6.66 × 10^2^	3.20 × 10^4^	9.91 × 10^2^	2.32 × 10^3^	8.92 × 10^4^	7.98 × 10^4^	1.04 × 10^4^	7.32 × 10^3^
	Std	2.36 × 10^2^	2.54 × 10^2^	2.92 × 10^2^	1.53 × 10^4^	4.93 × 10^2^	7.88 × 10^2^	7.42 × 10^3^	1.49 × 10^4^	4.97 × 10^3^	6.69 × 10^3^
F22	Mean	2.29 × 10^2^	**2.22 × 10^2^**	2.79 × 10^2^	5.76 × 10^3^	2.97 × 10^2^	1.25 × 10^3^	1.08 × 10^4^	1.04 × 10^4^	3.83 × 10^2^	7.68 × 10^2^
	Std	1.21 × 10^1^	1.05 × 10^1^	3.36 × 10^1^	2.03 × 10^3^	4.43 × 10^1^	2.09 × 10^3^	1.64 × 10^3^	2.38 × 10^3^	8.95 × 10^1^	8.34 × 10^2^
F23	Mean	**3.84 × 10^2^**	4.26 × 10^2^	8.62 × 10^2^	4.76 × 10^4^	4.61 × 10^3^	5.04 × 10^3^	9.79 × 10^4^	9.05 × 10^4^	1.83 × 10^4^	1.46 × 10^4^
	Std	2.51 × 10^2^	2.68 × 10^2^	2.66 × 10^2^	1.05 × 10^4^	3.84 × 10^3^	2.32 × 10^3^	6.80 × 10^3^	6.97 × 10^3^	5.78 × 10^3^	9.62 × 10^3^
F24	Mean	**2.81 × 10^2^**	3.29 × 10^2^	5.44 × 10^2^	3.78 × 10^4^	3.30 × 10^3^	3.92 × 10^3^	6.24 × 10^4^	5.85 × 10^4^	1.03 × 10^4^	1.16 × 10^4^
	Std	1.71 × 10^2^	2.18 × 10^2^	2.13 × 10^2^	7.42 × 10^3^	1.99 × 10^3^	2.15 × 10^3^	2.87 × 10^3^	4.44 × 10^3^	3.41 × 10^3^	4.27 × 10^3^
F25	Mean	**8.33 × 10^2^**	8.45 × 10^2^	9.90 × 10^2^	4.66 × 10^3^	9.65 × 10^2^	1.48 × 10^3^	2.41 × 10^4^	1.59 × 10^4^	1.97 × 10^3^	1.36 × 10^3^
	Std	9.69 × 10^1^	6.06 × 10^1^	1.30 × 10^2^	1.29 × 10^3^	1.87 × 10^2^	3.05 × 10^2^	4.15 × 10^3^	3.56 × 10^3^	5.27 × 10^2^	2.99 × 10^2^
F26	Mean	1.52 × 10^3^	**1.50 × 10^3^**	1.53 × 10^3^	4.42 × 10^3^	1.67 × 10^3^	3.67 × 10^3^	3.18 × 10^4^	1.58 × 10^4^	2.14 × 10^3^	3.48 × 10^3^
	Std	1.17 × 10^2^	1.11 × 10^2^	1.09 × 10^2^	1.58 × 10^3^	5.62 × 10^1^	1.63 × 10^3^	2.23 × 10^4^	8.84 × 10^3^	2.96 × 10^2^	1.07 × 10^3^
F27	Mean	**5.61 × 10^2^**	5.85 × 10^2^	6.74 × 10^2^	1.35 × 10^3^	6.06 × 10^2^	1.07 × 10^3^	4.29 × 10^3^	3.12 × 10^3^	7.68 × 10^2^	8.49 × 10^2^
	Std	2.85 × 10^1^	3.78 × 10^1^	9.59 × 10^1^	4.36 × 10^2^	4.84 × 10^1^	2.74 × 10^2^	9.32 × 10^2^	9.14 × 10^2^	7.34 × 10^1^	1.96 × 10^2^
F28	Mean	**1.82 × 10^2^**	2.40 × 10^2^	3.64 × 10^2^	9.71 × 10^2^	5.07 × 10^2^	6.70 × 10^2^	5.14 × 10^3^	3.54 × 10^3^	5.69 × 10^2^	7.47 × 10^2^
	Std	1.98 × 10^2^	1.65 × 10^2^	1.39 × 10^2^	2.48 × 10^2^	4.97 × 10^1^	4.07 × 10^2^	1.11 × 10^3^	8.72 × 10^2^	5.46 × 10^1^	2.09 × 10^2^
F29	Mean	**3.00 × 10^4^**	1.03 × 10^5^	4.68 × 10^5^	3.06 × 10^8^	2.24 × 10^6^	1.03 × 10^9^	9.90 × 10^13^	2.15 × 10^13^	6.43 × 10^8^	1.37 × 10^8^
	Std	1.83 × 10^4^	1.33 × 10^5^	8.35 × 10^5^	3.97 × 10^8^	2.78 × 10^6^	1.13 × 10^9^	1.38 × 10^14^	3.84 × 10^13^	5.29 × 10^8^	1.90 × 10^8^
F30	Mean	3.11 × 10^5^	4.86 × 10^5^	**2.84 × 10^5^**	2.34 × 10^10^	9.86 × 10^5^	2.11 × 10^9^	2.11 × 10^14^	8.24 × 10^13^	4.26 × 10^9^	8.92 × 10^9^
	Std	1.71 × 10^5^	2.74 × 10^5^	2.00 × 10^5^	1.11 × 10^11^	1.86 × 10^6^	3.83 × 10^9^	3.97 × 10^14^	2.44 × 10^14^	3.70 × 10^9^	1.31 × 10^10^

Values are given to three significant figures in scientific notation. The lowest mean error on each function is in bold.

**Table A4:** Error statistics of MSCPO and the comparison algorithms on the CEC2017 suite (D=100).

Function	Metric	MSCPO	CPO	SBOA	BKA	RBMO	DBO	HHO	WOA	GWO	PSO
F1	Mean	**1.30 × 10^7^**	3.09 × 10^8^	3.59 × 10^9^	1.45 × 10^11^	1.71 × 10^10^	4.23 × 10^10^	2.55 × 10^11^	2.37 × 10^11^	5.02 × 10^10^	4.76 × 10^10^
	Std	4.89 × 10^6^	1.33 × 10^8^	2.61 × 10^9^	1.78 × 10^10^	4.78 × 10^9^	5.23 × 10^10^	1.16 × 10^10^	1.71 × 10^10^	1.12 × 10^10^	1.97 × 10^10^
F3	Mean	**1.82 × 10^4^**	4.74 × 10^4^	3.52 × 10^4^	1.61 × 10^5^	1.34 × 10^5^	1.66 × 10^5^	3.57 × 10^5^	3.65 × 10^5^	1.11 × 10^5^	5.08 × 10^4^
	Std	4.81 × 10^3^	8.78 × 10^3^	8.21 × 10^3^	2.15 × 10^4^	2.94 × 10^4^	6.92 × 10^4^	2.03 × 10^4^	2.78 × 10^4^	1.87 × 10^4^	2.32 × 10^4^
F4	Mean	**5.25 × 10^2^**	6.49 × 10^2^	1.04 × 10^3^	2.08 × 10^4^	1.80 × 10^3^	7.36 × 10^3^	1.08 × 10^5^	8.06 × 10^4^	4.71 × 10^3^	6.81 × 10^3^
	Std	6.09 × 10^1^	9.68 × 10^1^	1.81 × 10^2^	7.45 × 10^3^	4.09 × 10^2^	1.05 × 10^4^	1.48 × 10^4^	1.71 × 10^4^	1.40 × 10^3^	4.24 × 10^3^
F5	Mean	**1.49 × 10^3^**	1.90 × 10^3^	5.99 × 10^3^	1.45 × 10^5^	1.86 × 10^4^	3.80 × 10^4^	3.20 × 10^5^	2.84 × 10^5^	5.03 × 10^4^	5.36 × 10^4^
	Std	1.51 × 10^2^	2.49 × 10^2^	2.99 × 10^3^	1.94 × 10^4^	5.79 × 10^3^	4.96 × 10^4^	1.78 × 10^4^	2.27 × 10^4^	1.12 × 10^4^	1.84 × 10^4^
F6	Mean	1.82 × 10^−3^	2.32 × 10^−2^	**1.62 × 10^−3^**	4.33 × 10^−3^	1.26 × 10^−2^	4.96 × 10^−3^	5.59 × 10^−2^	6.29 × 10^−2^	2.95 × 10^−3^	3.46 × 10^−3^
	Std	1.81 × 10^−3^	6.23 × 10^−3^	7.93 × 10^−4^	2.05 × 10^−3^	3.76 × 10^−3^	4.34 × 10^−3^	8.40 × 10^−3^	1.11 × 10^−2^	1.29 × 10^−3^	1.62 × 10^−3^
F7	Mean	**2.33 × 10^3^**	1.59 × 10^4^	1.60 × 10^5^	5.26 × 10^6^	5.83 × 10^5^	1.99 × 10^6^	1.01 × 10^7^	9.28 × 10^6^	1.71 × 10^6^	1.72 × 10^6^
	Std	3.97 × 10^2^	4.93 × 10^3^	1.05 × 10^5^	7.10 × 10^5^	1.54 × 10^5^	2.13 × 10^6^	4.32 × 10^5^	6.30 × 10^5^	3.71 × 10^5^	5.14 × 10^5^
F8	Mean	1.44 × 10^3^	1.94 × 10^3^	1.60 × 10^3^	2.28 × 10^3^	1.73 × 10^3^	2.45 × 10^3^	3.01 × 10^3^	3.00 × 10^3^	**1.34 × 10^3^**	1.97 × 10^3^
	Std	2.34 × 10^2^	1.28 × 10^2^	1.95 × 10^2^	1.63 × 10^2^	1.21 × 10^2^	3.37 × 10^2^	1.45 × 10^2^	1.62 × 10^2^	3.14 × 10^2^	3.60 × 10^2^
F9	Mean	2.93 × 10^1^	**2.73 × 10^1^**	5.79 × 10^1^	9.11 × 10^1^	5.53 × 10^1^	1.13 × 10^2^	2.63 × 10^2^	2.56 × 10^2^	5.53 × 10^1^	5.72 × 10^1^
	Std	6.02 × 10^0^	7.12 × 10^0^	6.93 × 10^0^	1.52 × 10^1^	1.65 × 10^1^	4.01 × 10^1^	2.41 × 10^1^	3.82 × 10^1^	1.30 × 10^1^	1.70 × 10^1^
F10	Mean	1.96 × 10^4^	2.92 × 10^4^	**1.74 × 10^4^**	1.99 × 10^4^	2.40 × 10^4^	2.62 × 10^4^	3.10 × 10^4^	3.10 × 10^4^	2.34 × 10^4^	2.05 × 10^4^
	Std	4.47 × 10^3^	6.75 × 10^2^	1.71 × 10^3^	1.42 × 10^3^	1.59 × 10^3^	5.19 × 10^3^	9.68 × 10^2^	1.02 × 10^3^	7.31 × 10^3^	3.47 × 10^3^
F11	Mean	**5.59 × 10^4^**	1.14 × 10^5^	1.85 × 10^5^	7.23 × 10^7^	4.94 × 10^5^	1.13 × 10^6^	9.81 × 10^9^	4.72 × 10^9^	2.83 × 10^7^	2.98 × 10^7^
	Std	2.77 × 10^4^	3.57 × 10^4^	4.76 × 10^4^	1.25 × 10^8^	9.69 × 10^4^	2.37 × 10^5^	3.69 × 10^9^	2.75 × 10^9^	1.01 × 10^8^	5.46 × 10^7^
F12	Mean	**1.67 × 10^7^**	3.15 × 10^7^	1.71 × 10^8^	5.13 × 10^10^	4.82 × 10^8^	2.75 × 10^9^	1.83 × 10^11^	1.50 × 10^11^	9.99 × 10^9^	1.65 × 10^10^
	Std	7.38 × 10^6^	1.26 × 10^7^	1.15 × 10^8^	1.58 × 10^10^	3.19 × 10^8^	1.18 × 10^9^	1.84 × 10^10^	2.04 × 10^10^	6.93 × 10^9^	1.31 × 10^10^
F13	Mean	**1.18 × 10^6^**	2.75 × 10^6^	4.02 × 10^7^	7.74 × 10^10^	7.27 × 10^8^	3.36 × 10^9^	3.31 × 10^11^	2.40 × 10^11^	1.70 × 10^10^	2.68 × 10^10^
	Std	5.75 × 10^5^	1.87 × 10^6^	2.01 × 10^7^	2.70 × 10^10^	1.67 × 10^9^	3.10 × 10^9^	4.82 × 10^10^	3.45 × 10^10^	8.98 × 10^9^	1.89 × 10^10^
F14	Mean	**1.42 × 10^6^**	2.51 × 10^6^	1.65 × 10^6^	2.01 × 10^6^	8.13 × 10^6^	1.24 × 10^7^	2.37 × 10^8^	1.38 × 10^8^	4.33 × 10^6^	5.36 × 10^6^
	Std	9.67 × 10^5^	1.70 × 10^6^	6.65 × 10^5^	1.40 × 10^6^	4.10 × 10^6^	1.25 × 10^7^	1.35 × 10^8^	8.98 × 10^7^	3.66 × 10^6^	3.16 × 10^6^
F15	Mean	**2.08 × 10^5^**	2.28 × 10^5^	1.26 × 10^6^	9.26 × 10^9^	2.71 × 10^7^	1.18 × 10^9^	4.95 × 10^10^	3.78 × 10^10^	1.50 × 10^9^	1.32 × 10^9^
	Std	7.74 × 10^4^	4.17 × 10^4^	1.19 × 10^6^	4.19 × 10^9^	1.11 × 10^8^	4.78 × 10^9^	6.13 × 10^9^	7.17 × 10^9^	1.34 × 10^9^	1.66 × 10^9^
F16	Mean	**6.54 × 10^3^**	7.95 × 10^3^	1.77 × 10^4^	5.46 × 10^8^	1.33 × 10^5^	1.94 × 10^6^	1.18 × 10^10^	7.97 × 10^9^	3.26 × 10^7^	3.26 × 10^7^
	Std	4.07 × 10^3^	4.28 × 10^3^	1.18 × 10^4^	4.46 × 10^8^	1.69 × 10^5^	1.93 × 10^6^	3.39 × 10^9^	2.66 × 10^9^	6.30 × 10^7^	4.59 × 10^7^
F17	Mean	**5.86 × 10^3^**	2.00 × 10^4^	2.52 × 10^4^	1.10 × 10^11^	1.59 × 10^5^	7.60 × 10^10^	6.66 × 10^15^	2.30 × 10^15^	4.05 × 10^9^	2.35 × 10^8^
	Std	1.65 × 10^3^	1.11 × 10^4^	2.19 × 10^4^	3.17 × 10^11^	4.79 × 10^4^	3.67 × 10^11^	4.06 × 10^15^	2.74 × 10^15^	2.17 × 10^10^	1.26 × 10^9^
F18	Mean	**1.42 × 10^6^**	1.79 × 10^6^	2.10 × 10^6^	2.05 × 10^6^	8.63 × 10^6^	1.65 × 10^7^	2.56 × 10^8^	2.11 × 10^8^	5.08 × 10^6^	9.10 × 10^6^
	Std	1.03 × 10^6^	1.17 × 10^6^	1.17 × 10^6^	1.13 × 10^6^	6.68 × 10^6^	1.37 × 10^7^	2.40 × 10^8^	2.72 × 10^8^	3.23 × 10^6^	9.74 × 10^6^
F19	Mean	5.80 × 10^4^	**5.70 × 10^4^**	1.40 × 10^6^	6.22 × 10^11^	1.03 × 10^9^	2.04 × 10^10^	3.54 × 10^15^	2.24 × 10^15^	2.92 × 10^11^	6.13 × 10^11^
	Std	1.87 × 10^4^	1.59 × 10^4^	5.28 × 10^6^	1.28 × 10^12^	2.80 × 10^9^	2.44 × 10^10^	2.30 × 10^15^	2.78 × 10^15^	1.10 × 10^12^	2.68 × 10^12^
F20	Mean	**8.61 × 10^2^**	1.55 × 10^3^	2.39 × 10^3^	1.56 × 10^4^	3.37 × 10^3^	7.39 × 10^3^	3.38 × 10^4^	3.26 × 10^4^	3.97 × 10^3^	4.40 × 10^3^
	Std	2.66 × 10^2^	6.58 × 10^2^	9.02 × 10^2^	2.91 × 10^3^	1.19 × 10^3^	2.45 × 10^3^	4.49 × 10^3^	5.98 × 10^3^	1.12 × 10^3^	2.03 × 10^3^
F21	Mean	**1.69 × 10^3^**	2.07 × 10^3^	5.66 × 10^3^	1.52 × 10^5^	1.75 × 10^4^	2.53 × 10^4^	2.45 × 10^5^	2.31 × 10^5^	4.89 × 10^4^	4.95 × 10^4^
	Std	2.96 × 10^2^	2.44 × 10^2^	3.30 × 10^3^	2.08 × 10^4^	4.70 × 10^3^	2.41 × 10^4^	8.47 × 10^3^	1.27 × 10^4^	1.13 × 10^4^	1.87 × 10^4^
F22	Mean	**3.10 × 10^2^**	3.20 × 10^2^	3.83 × 10^2^	1.60 × 10^4^	5.70 × 10^2^	5.74 × 10^3^	2.53 × 10^4^	2.56 × 10^4^	8.50 × 10^2^	1.38 × 10^3^
	Std	2.73 × 10^1^	1.92 × 10^1^	4.56 × 10^1^	2.15 × 10^3^	1.12 × 10^2^	7.39 × 10^3^	1.49 × 10^3^	1.61 × 10^3^	1.71 × 10^2^	3.27 × 10^2^
F23	Mean	**7.74 × 10^2^**	2.60 × 10^3^	9.72 × 10^3^	1.03 × 10^5^	1.79 × 10^4^	2.47 × 10^4^	1.26 × 10^5^	1.24 × 10^5^	4.32 × 10^4^	3.22 × 10^4^
	Std	4.28 × 10^2^	9.99 × 10^2^	4.04 × 10^3^	3.29 × 10^3^	5.97 × 10^3^	1.37 × 10^4^	2.15 × 10^3^	2.78 × 10^3^	1.33 × 10^4^	7.31 × 10^3^
F24	Mean	**1.24 × 10^3^**	5.48 × 10^3^	2.02 × 10^4^	1.31 × 10^5^	3.17 × 10^4^	2.97 × 10^4^	1.77 × 10^5^	1.70 × 10^5^	5.32 × 10^4^	4.48 × 10^4^
	Std	6.22 × 10^2^	2.17 × 10^3^	1.45 × 10^4^	8.44 × 10^3^	4.24 × 10^3^	1.27 × 10^4^	4.95 × 10^3^	6.73 × 10^3^	8.15 × 10^3^	1.07 × 10^4^
F25	Mean	**1.32 × 10^3^**	1.54 × 10^3^	1.87 × 10^3^	9.13 × 10^3^	1.98 × 10^3^	6.07 × 10^3^	3.54 × 10^4^	2.74 × 10^4^	3.94 × 10^3^	3.12 × 10^3^
	Std	8.44 × 10^1^	1.22 × 10^2^	1.91 × 10^2^	1.56 × 10^3^	2.74 × 10^2^	5.08 × 10^3^	5.82 × 10^3^	4.76 × 10^3^	7.81 × 10^2^	1.44 × 10^3^
F26	Mean	**3.47 × 10^3^**	3.48 × 10^3^	3.70 × 10^3^	6.86 × 10^4^	4.06 × 10^3^	2.11 × 10^4^	1.39 × 10^5^	1.12 × 10^5^	9.67 × 10^3^	1.77 × 10^4^
	Std	5.36 × 10^1^	5.58 × 10^1^	1.62 × 10^2^	3.40 × 10^4^	4.15 × 10^2^	8.46 × 10^3^	5.96 × 10^4^	4.69 × 10^4^	1.56 × 10^3^	7.68 × 10^3^
F27	Mean	**7.68 × 10^2^**	8.12 × 10^2^	1.06 × 10^3^	3.14 × 10^3^	8.30 × 10^2^	1.67 × 10^3^	7.90 × 10^3^	6.72 × 10^3^	1.35 × 10^3^	1.33 × 10^3^
	Std	6.33 × 10^1^	5.93 × 10^1^	2.27 × 10^2^	9.18 × 10^2^	9.23 × 10^1^	4.18 × 10^2^	1.48 × 10^3^	1.36 × 10^3^	1.35 × 10^2^	4.27 × 10^2^
F28	Mean	**4.59 × 10^2^**	5.41 × 10^2^	6.70 × 10^2^	5.69 × 10^3^	1.43 × 10^3^	4.62 × 10^3^	2.30 × 10^4^	1.78 × 10^4^	1.73 × 10^3^	2.78 × 10^3^
	Std	9.23 × 10^1^	6.90 × 10^1^	4.96 × 10^1^	1.07 × 10^3^	4.01 × 10^2^	3.14 × 10^3^	3.28 × 10^3^	2.81 × 10^3^	4.05 × 10^2^	1.08 × 10^3^
F29	Mean	**3.66 × 10^5^**	1.48 × 10^6^	1.02 × 10^7^	9.54 × 10^12^	1.85 × 10^8^	3.18 × 10^10^	5.54 × 10^15^	1.58 × 10^15^	3.43 × 10^11^	2.06 × 10^11^
	Std	6.71 × 10^5^	1.71 × 10^6^	1.62 × 10^7^	4.82 × 10^13^	1.77 × 10^8^	7.30 × 10^10^	5.76 × 10^15^	1.91 × 10^15^	1.23 × 10^12^	8.44 × 10^11^
F30	Mean	**1.39 × 10^6^**	2.51 × 10^6^	2.35 × 10^7^	1.45 × 10^12^	4.36 × 10^9^	2.95 × 10^10^	2.85 × 10^15^	1.68 × 10^15^	2.06 × 10^11^	3.59 × 10^11^
	Std	1.11 × 10^6^	1.75 × 10^6^	3.47 × 10^7^	4.10 × 10^12^	8.37 × 10^9^	1.61 × 10^10^	2.57 × 10^15^	1.81 × 10^15^	8.04 × 10^11^	1.66 × 10^12^

Values are given to three significant figures in scientific notation. The lowest mean error on each function is in bold.
